# New Thienobenzo/Naphtho-Triazoles as Butyrylcholinesterase Inhibitors: Design, Synthesis and Computational Study

**DOI:** 10.3390/ijms24065879

**Published:** 2023-03-20

**Authors:** Milena Mlakić, Ida Selec, Irena Ćaleta, Ilijana Odak, Danijela Barić, Ana Ratković, Krešimir Molčanov, Irena Škorić

**Affiliations:** 1 Department of Organic Chemistry, Faculty of Chemical Engineering and Technology, University of Zagreb, Marulićev Trg 19, HR-10000 Zagreb, Croatia; 2 Chemistry, Selvita Ltd., Prilaz Baruna Filipovića 29, HR-10000 Zagreb, Croatia; 3 Department of Chemistry, Faculty of Science and Education, University of Mostar, Matice hrvatske bb, 88000 Mostar, Bosnia and Herzegovina; 4 Group for Computational Life Sciences, Division of Physical Chemistry, Ruđer Bošković Institute, Bijenička Cesta 54, HR-10000 Zagreb, Croatia; 5 Division of Physical Chemistry, Ruđer Bošković Institute, Bijenička Cesta 54, HR-10000 Zagreb, Croatia

**Keywords:** cholinesterases, photochemistry, synthesis, 1,2,3-triazoles, inhibition

## Abstract

This study aims to test the inhibition potency of new thienobenzo/naphtho-triazoles toward cholinesterases, evaluate their inhibition selectivity, and interpret the obtained results by molecular modeling. The synthesis of 19 new thienobenzo/naphtho-triazoles by two different approaches resulted in a large group of molecules with different functionalities in the structure. As predicted, most prepared molecules show better inhibition of the enzyme butyrylcholinesterase (BChE), considering that the new molecules were designed according to the previous results. Interestingly, the binding affinity of BChE for even seven new compounds (**1**, **3**, **4**, **5**, **6**, **9**, and **13**) was similar to that reported for common cholinesterase inhibitors. According to computational study, the active thienobenzo- and naphtho-triazoles are accommodated by cholinesterases through H-bonds involving one of the triazole’s nitrogens, π-π stacking between the aromatic moieties of the ligand and aromatic residues of the active sites of cholinesterases, as well as π-alkyl interactions. For the future design of cholinesterase inhibitors and search for therapeutics for neurological disorders, compounds with a thienobenzo/naphtho-triazole skeleton should be considered.

## 1. Introduction

Acetylcholinesterase (AChE) and butyrylcholinesterase (BChE) are two related enzymes existing in plants and vertebrates. In humans, the enzymes share about half of their amino acid sequence identity [1]. The crucial divergence in their active site is the presence of 14 aromatic amino acid residues in AChE, which is in tune with eight aromatic and six aliphatic residues in BChE [2], enabling BChE to hydrolyze substantially more molecules than AChE [3,4,5,6]. Enzyme AChE plays an indispensable physiological role in the body controlling the channeling of nerve impulses in the cholinergic synapses of the central and peripheral nervous system by hydrolysis of the neurotransmitter acetylcholine. Moreover, AChE takes part in many other processes, for instance, dopamine neuronal activation and the formation of amyloid fibers characteristic of Alzheimer’s disease [7,8,9]. The role of BChE is appointed to the catalytic and stoichiometric detoxification of xenobiotics (for example, organophosphates, cocaine, aspirin, etc.) and the bioactivation of drugs (heroin, etc.) [10,11]. It is important to emphasize that BChE can co-regulate cholinergic neurotransmission, effectively catalyzing acetylcholine hydrolysis [12]. It was established that high BChE levels are interconnected with the distinctive neuropathologic characteristic of Alzheimer’s disease (AD) [13,14]. Both enzymes BChE and AChE are pharmacologically appropriate targets in neurodegenerative disorders. The treatment of these disorders currently comprises cholinesterase inhibitors such as galantamine, donepezil, or rivastigmine [15]. Therefore, many other compounds acting as inhibitors of cholinesterases are contemplated as potentially being AD beneficial [16,17,18,19,20].

**Figure 1:**
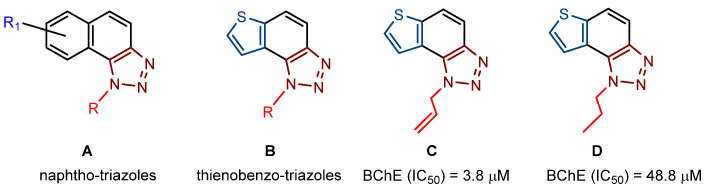
Previously investigated thienobenzo/naphtho-triazoles (**A**–**D**) with cholinesterase inhibitory activity.

In our previous research [21,22], we were able to design and synthesize new potential cholinesterase enzyme inhibitors, which structurally belonged to naphtho/thienobenzo-triazoles (Figure 1, structures A and B). In doing so, we also showed some selective interconnection of the cholinesterase inhibitory and anti-inflammatory activity with the inhibition of TNF*α* cytokine production. The most potent BChE inhibitor was the allyl-thienobenzotriazole (Figure 1, structure C, IC_50_ 3.8 μM; IC_50_ (galantamine) = 7.9 μM), showing good TNF*α* production inhibition in lipopolysaccharide (LPS)-stimulated human peripheral blood mononuclear cells (PBMCs) simultaneously. Very good inhibitory activity against BChE was also shown by the *n*-propyl derivative (Figure 1, structure D, IC_50_ 48.8 μM). Additionally, experimental results showed a much more potent anti-inflammatory effect of naphtho-triazoles (Figure 1, structure A) compared to thienobenzo-triazoles (Figure 1, structure B).

Contrary to that fact, the testing of inhibitory activity confirmed that thienobenzo-triazoles (Figure 1, structure B) were more potent and selective BChE inhibitors than the naphtho-triazoles (Figure 1, structure A). Regarding the presented findings, in the design of new cholinesterase inhibitors (especially for BChE [23]), it is concluded that the focus should be on thienobenzo-triazoles. As they were previously obtained by the photochemical methodology [21,22], the same electrocyclization reaction was applied in this research as well. However, an alternative approach to obtain target structures is introduced for cases where photocyclization cannot be successfully carried out or applied. The alternative reaction pathway gives a wide range of molecules with either dihydro-thienobenzo-triazole moiety or the final aromatized thienobenzo-triazole core to evaluate their impact on the cholinesterase inhibitory activity. The molecular docking of the selected new thienobenzo/naphtho-triazoles into the active site of AChE and BChE provides insight into the formed complexes’ structure and enables the identification of stabilizing interactions between the potential inhibitor and the enzyme. In this research, 19 thienobenzo/naphtho-triazoles are synthesized, differing in the substituent attached to the triazole ring or in the aromatic character of the central ring. This study aims to test their inhibition potency toward cholinesterases (especially BChE), evaluate their inhibition selectivity, and identify the most significant interactions responsible for experimentally obtained inhibitory potential. 

## 2. Results and Discussion

### 2.1. Synthesis of New Thienobenzo/Naphtho-Triazoles ***1–19***

**Scheme 1:**
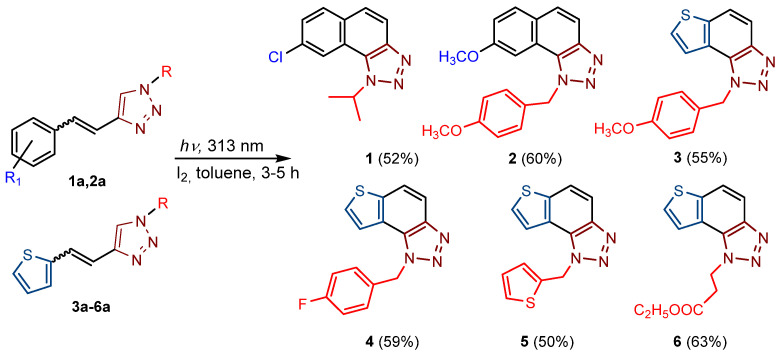
Reaction pathway for the synthesis of naphtho-triazoles **1** and **2** [21] and thienobenzo-triazoles **3**–**6**. Numbers given in parentheses represent isolated yields.

New naphtho-triazoles **1** and **2** and thienobenzo-triazoles **3**–**6** were prepared by photocyclization from the corresponding triazolo-stilbenes **1a** and **2a** and triazolo-thienostilbenes **3a**–**6a** (Scheme 1). Triazolo-stilbenes **1a** and **2a** and triazolo-thienostilbenes **3a**–**6a**, as mixtures of *cis*- and *trans*-isomers, were prepared by the Wittig reaction according to the described procedure in moderate to good yields (45–60%) [21,24] and transformed to the new naphtho-triazoles **1** and **2** and thienobenzo-triazoles **3**–**6** as new biological targets (Scheme 1). The Wittig reactions were performed with aryl- and 2-thienyl-phosphonium salts in absolute EtOH, sodium ethoxide, and different triazole aldehydes. The reaction mixtures were left to stir for 24 h at room temperature with a nitrogen balloon. Although compounds **1** and **2**, the only naphtho-triazoles in this research, are known from a previous study [21], they were prepared again because their inhibitory activity was not tested due to insufficient samples in the previous analysis. In aerobic conditions, a mixture of isomers of compounds **1a–**-**6a** was dissolved in toluene (~2.5 × 10^−3^ M) and irradiated with 10 UV lamps at 313 nm in a quartz vessel with the addition of a catalytic amount of iodine in a photochemical reactor Rayonet for 3–5 h to achieve almost complete conversion. The obtained photoproducts **1**–**6** were isolated in high yields (Scheme 1, 50–63%) and completely characterized by NMR spectroscopy (see Section 3 and Figures S1–S79 in ESI). The isolated yields of **1**–**6** from the photochemical reaction are quite similar regardless of the substituent on the triazole ring, and there are no by-products, while the conversions are over 90%. The formation of the electrocyclization photoproducts **1**–**6** was generally accompanied by the appearance of some high-molecular-weight products, which were not investigated. In their ^1^H NMR spectra, the disappearance of the ethylenic protons’ signals and the protons’ singlets on the 1,2,3-triazole rings can be detected compared with the starting triazolo(thieno) stilbenes **1a**–**6a**. 

**Scheme 2:**
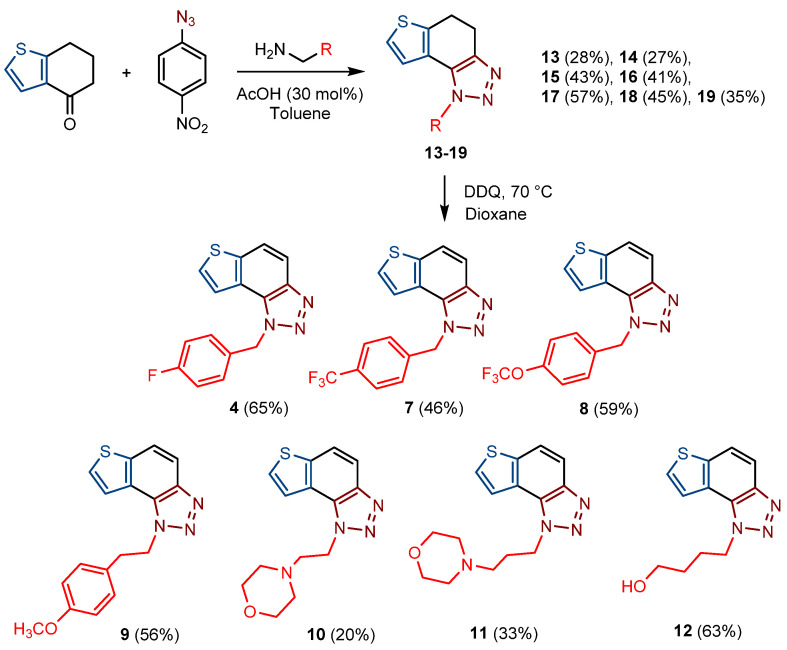
Reaction pathway for the synthesis of thienobenzo-triazoles **4**, **7**–**12** and their non-aromatized precursors **13**–**19** (Numbers given in parentheses represent isolated yields.).

For the synthesis of other new thienobenzo-triazoles **7**–**12**, the alternative synthetic route was used (Scheme 2) and compared with the photochemical synthesis on the example of the fluorine derivative **4**. The reactions started from 4-keto-4,5,6,7-tetrahydrothianaphthene dissolved in toluene, followed by the addition of 1-azido-4-nitro-benzene, corresponding amines, acetic acid, and 4 Å molecular sieves. The reaction mixtures were stirred at 100 °C overnight, cooled to room temperature and worked up. The obtained crude dihydro-thienobenzo-triazoles **13**–**19** were dissolved in dioxane, and the 2,3-dichloro-5,6-dicyano-1,4-benzoquinone was added to the solution. Reaction mixtures were stirred at 70 °C. After leaving them overnight, reaction mixtures were cooled to room temperature and worked up. Previous research [21] showed that photocyclization is not always successful depending on the substituent on the triazole ring. Hence, developing a different synthetic route is very profitable in the long run. According to the literature [25,26,27], in the first step, a solution of tetrahydrothianaphthene in toluene, with 1-azido-4-nitro-benzene, the corresponding amine, acetic acid, and 4 Å molecular sieves as a reaction mixture was stirred at 100 °C overnight, then worked up and purified to obtain final products, non-aromatized dihydro-thienobenzo-triazoles **13**–**19** (Scheme 2, 27–57%). In the second step, the dihydro derivatives **13**–**19** were aromatized using DDQ (2,3-dichloro-5,6-dicyano-1,4-benzoquinone) in dioxane by stirring the reaction mixture at 70 °C overnight, then working it up and purifying it to obtain the final product, aromatized thienobenzo-triazoles **4**, **7**–**12** (Scheme 2, see also Section 3).

**Figure 2:**
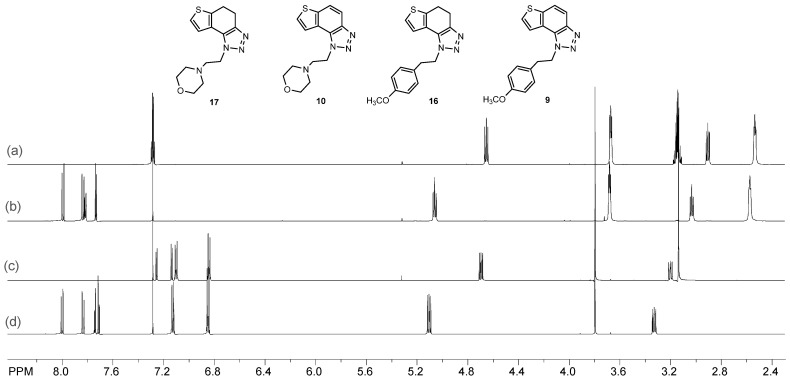
Comparison of the ^1^H NMR spectra (CDCl_3_) of investigated compounds (**a**) **17**; (**b**) **10**; (**c**) **16**; (**d**) **9**.

The obtained products **4**, **7**–**12** were isolated in moderate to good isolated yields (20–65%) and completely characterized by NMR spectroscopy (see Section 3 and Figures S1–S79 in ESI). In their ^1^H NMR spectra, the disappearance of the aliphatic protons in the central ring and two new doublets for the new aromatic protons of the same ring in comparison with the starting non-aromatized analogs **13**–**19** can be detected (See Section 3, and Figures S1–S79 in ESI and Figure 2 for the transformation of **17** to **10**, and **16** into **9**). For most derivatives, the isolated yields in the two reaction stages (Scheme 2) are within similar limits to the path via photocyclization (Scheme 1). Only the synthesis of derivatives via an alternative route is somewhat less efficient for compounds **13** and **14** for the first steps, and compounds **10** and **11** for the second stage of synthesis (Scheme 2). Specifically for the synthesis of derivative **4**, the overall yield is slightly better for the first synthetic route. However, this new approach via condensation and aromatization applies to all derivatives, and remains an important choice in further research.

### 2.2. Inhibitory Activity of Thienobenzo/Naphtho-Triazoles ***1****–****19*** toward Enzymes Cholinesterases

Given the promising results of the previous study [21], we conducted this research to test the inhibitory activity of newly synthesized thienobenzo/naphtho-triazoles **1**–**19** toward cholinesterase enzymes, primarily BChE. Introducing new substituents on the triazole ring can show whether there is an additional improvement in biological activity; it can identify the relationship between structure and inhibitory activity in the new group of prepared analogs. 

While compound **2** had the best anti-inflammatory activity of all the tested compounds in the previous series [21], it shows no inhibitory activity towards cholinesterases. From the aforementioned experimental data, it can be concluded that the anti-inflammatory effect of compound **2** is not caused by the inhibition of cholinesterase but by some other target, such as the TNFα receptor, which remains to be further investigated. However, compound **1**, with ten times weaker anti-inflammatory activity than **2**, is one of the best potential cholinesterase inhibitors among all synthesized naphtho-triazoles so far (Table 1 and Table S3). Although it has excellent enzymatic activity, additional work on SAR (structure-activity relationship) should be done to see the effect in experiments related to a particular pathology, for instance, inflammation. For naphtho-triazoles, the suitable combination of substituents at the aryl and triazole ring can lead to promising inhibitory activity. The best inhibition of both enzymes among newly prepared thienobenzo-triazoles **3**–**19** was shown by compound **4**, possessing the *para*-F-benzyl substituent on the triazole ring (Table 1and Table S3). 

The comparison of molecules **4**, **7**–**12** with analogues **13**–**19** possessing a nonaromatic central ring indicates that the substituent on the triazole ring has more influence on the inhibitory activity than the structural feature related to whether the molecule’s central ring is aromatized. Notably, the IC_50_ values for BChE of compounds **1**, **3**, **4**, **5**, **6**, **9**, and **13** were similar to the common reversible cholinesterase inhibitor huperzine (IC_50_ 53.6 µM) [28] and just somewhat weaker than galantamine (IC_50_ 7.9 μM). In previous research, the *n*-propyl derivative of the thienobenzo-triazole (Figure 1, structure D) also showed very good inhibitory activity toward BChE (IC_50_ 48.8 μM), which is in the range of the commons mentioned above. Analogs **3**, **5**, and **9** stand out as the most effective BChE inhibitors: compound **9** is a selective compound toward BChE, showing the most intense inhibition among all new derivatives **1**–**19**. Of these three thienobenzotriazole analogs, two have a *para*-OCH_3_ group on the triazole ring (analogs **3** and **9**), and the third most prominent compound (**5**) has an additional thiophene nucleus. Interestingly, regarding the selective inhibitory activity towards AChE, dihydro-thienobenzo-triazole **17** (but not its aromatized analog) showed the best biological activity.

### 2.3. Computational Study of Thienobenzo/Naphtho-Triazoles ***1****–****19*** as Cholinesterase Inhibitors

**Figure 3:**
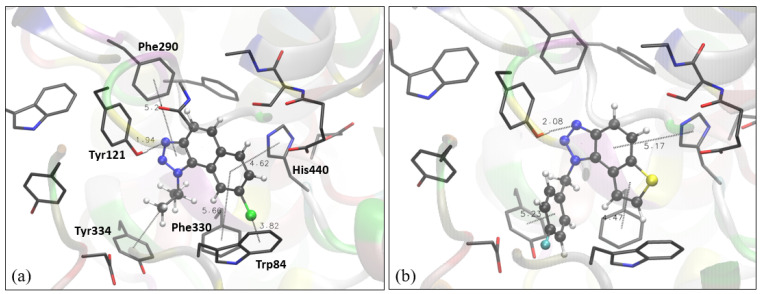
Structure of the active site of AChE docked with naphtho-triazole **1** (**a**) and thienobenzo-triazole **4** (**b**). Molecules of the potential inhibitors are presented using a ball-and-stick model.

Among experimentally evaluated compounds showing inhibitory activity toward both cholinesterases, the best results were achieved with naphtho-triazole **1** and thienobenzo-triazole **4**. The most promising candidate within the set of active molecules was compound **17**, while thienobenzo-triazole **9** showed excellent inhibitory potential toward BChE, as shown in Table 1. We performed a molecular docking study to obtain the structures and identify stabilizing interactions in the complexes between the best-performing compounds and the cholinesterases’ active site. The structures of the most stable complexes of **1** and **4** with the active site of AChE, obtained by docking, are presented in Figure 3. We used the crystal structure obtained from an electric eel (Electrophorus electricus, 1EEA.pdb), but it should be kept in mind that this structure does not completely reproduce the exact enzyme sequence as in the electric eel. Due to this structure’s low resolution, it includes an amino acid sequence taken from the tertiary structure of *Torpedo californica* AChE (see details in Section 3.6). 

The structure of the complex between the naphtho-triazole **1** and the active site of AChE reveals several stabilizing interactions. The orientation of the triazole toward the peripheral anionic site enables the formation of the hydrogen bond between one of the two sp^2^ nitrogens in the triazole ring and the -OH group of Tyr121. The strong interactions between triazole and Tyr121 have been reported in earlier studies on AChE inhibitors containing a triazole ring [29,30,31,32]. Furthermore, the isopropyl at triazole is involved in the π-alkyl interaction with Tyr334, another tyrosine from the peripheral anionic site. The “face-on” dispersive attraction between the chlorine of the ligand and Trp84 is identified: the distance between Cl and the center of the tryptophan aromatic ring is 3.8 Å, with the carbon atom (of the same ring) closest to the chlorine at 3.5 Å from the Cl. The difference between these two values up to 0.3 Å defines this Cl-π interaction as a “face-on”, while the difference larger than 0.3 Å classifies it as an “edge on” [33]. Finally, π-π stacking between the aromatic core of the ligand and some residues is also observed: the residues engaged in it are His440, Phe330, and Phe290. Figure 3b shows the complex between thienobenzo-triazole **4** and the active site of AChE. Similar to the structure of **1** docked into AChE, the hydrogen bond between triazole nitrogen and the -OH group of Tyr 121 is formed. The phenyl core of the ligand is involved in perpendicular π-π stacking with His330, while the thiophene ring achieves a similar interaction with Phe330. The fluorinated phenyl at triazole is also engaged in π-π stacking with Tyr334. 

**Figure 4:**
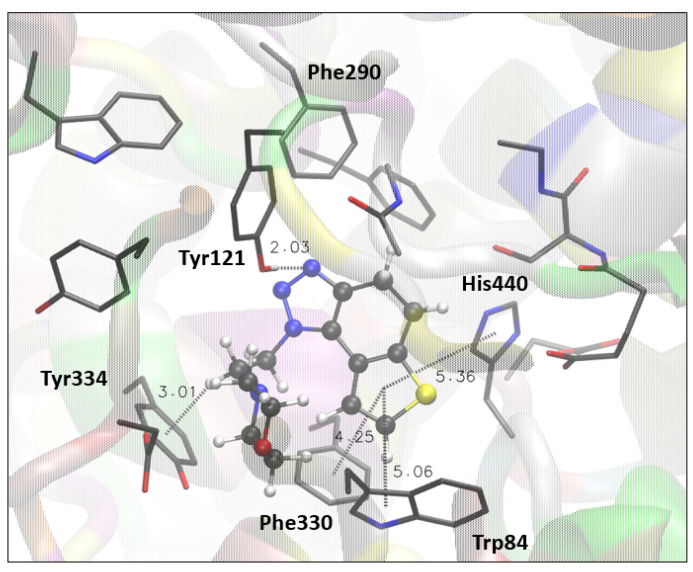
The structure of the complex between compound **17** (presented with a ball-and-stick model) and the active site of AChE. Hydrogens of the enzyme residues are omitted for clarity.

Compound **17**, the best-performing potential inhibitor of AChE, has a nonaromatic middle core. The structure of **17** docked in the active site of AChE (Figure 4) shows that **17** takes a pose similar to that of **1** and **4**, with a triazole ring oriented toward the peripheral anionic site. Thus, the H-bond between triazole nitrogen and the -OH group of Tyr121 occurs again.

The π-alkyl attraction is observed between the ethyl chain at triazole and Tyr334, while thiophene engages in π-π stacking with His440, Phe330, and Trp84. 

**Figure 5:**
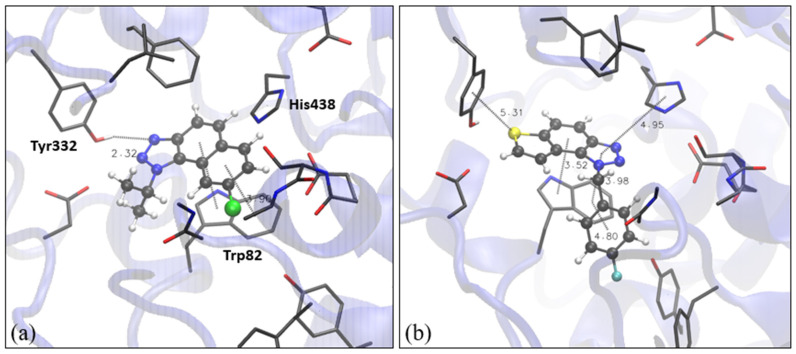
Structure of the active site of BChE docked with naphtho-triazole **1** (**a**) and thienobenzo-triazole **4** (**b**). Molecules of the potential inhibitors are presented using a ball-and-stick model.

As already mentioned, naphtho -triazole **1** and thienobenzo-triazole **4** were the most active among compounds that showed inhibitory potential toward both cholinesterases; therefore, we performed molecular docking of **1** and thienobenzo-triazole **4** into the active site of BChE too. Here, we used the structure of the human enzyme (see Section 3.6). Structures of obtained complexes are presented in Figure 5. The most stable pose obtained by the docking of compound 1 in the active site of BChE shows that, again, the H-bond with one of the tyrosines (Tyr332) in the peripheric anionic site is formed. Additional stabilizing interactions include π-π stacking between the naphthalene core of **1** and Trp82. For molecule **4** in BChE, the H-bond(s) between one of sp^2^ triazole nitrogens and the proton-donating residue was not observed; however, the sulfur of the thiophene is engaged in the interaction with Tyr332. The remaining aromatic rings of the ligand are involved in π-π stacking with Trp82 and His440, as presented in Figure 5b. 

**Figure 6:**
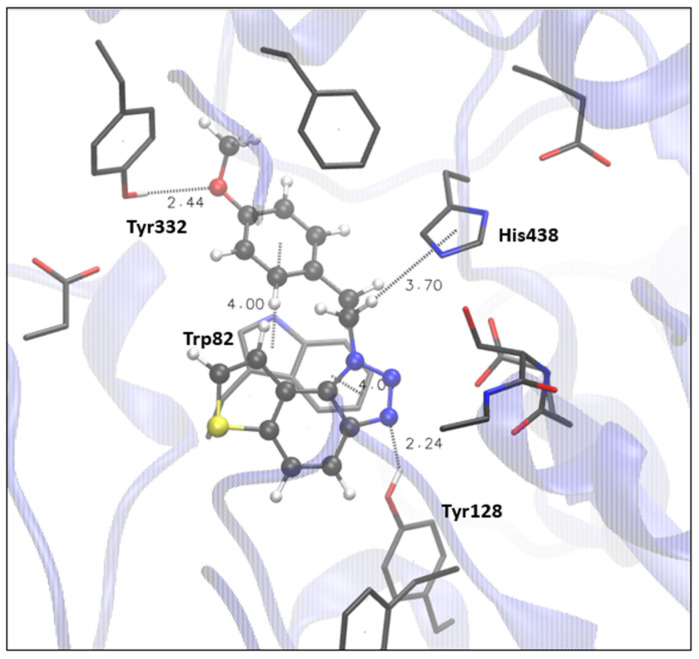
The structure of the complex between compound **9** (presented with a ball-and-stick model) and the active site of BChE. Hydrogens of the enzyme residues omitted for clarity.

Finally, the most promising candidate for inhibition of BChE is compound **9**, with an excellent experimental value of IC_50_ (Table 1). The structure of the BChE active site docked with **9**, shown in Figure 6, reveals that there are two possible H-bonds: one is formed between the triazole nitrogen and hydroxyl of the Tyr128 (analogously to the occurrence of hydrogen bond between triazole nitrogen and Tyr121 in AChE), and the other is possible due to the proximity of the oxygen of the ligand’s methoxy group and proton of the -OH group of the Tyr332. π-π stacking between the triazole ring of the ligand and the residue Trp82 is present, as well as π-alkyl interaction between the ethyl (that connects triazole and methoxy phenyl) and His438.

According to experimental data in Table 1, the most promising inhibitory potential toward BChE was shown by compounds **1**, **3**, **4**, **5**, **6**, **9**, and **13**. The inspection of their free energies of binding (Δ*G*_bind_) estimated by molecular docking shows that they are comparable with galantamine, whose Δ*G*_bind_ is −6.26 kcal mol^−1^, obtained by the same docking procedure. Free energies of binding for these compounds vary from −5.85 kcal mol^−1^ for compound **6** to −6.37 kcal mol^−1^ for molecule **9**, the latter being even slightly lower than the value for galantamine (Table S1). However, it should be considered that the docking study gives only a crude estimation of Δ*G*_bind_, and does not intend to offer an exact thermodynamical description of our systems. 

The results of the docking study can show the possibilities of the ligands’ placement into the active site. They can identify the main interactions between the new ligands and the enzymes, thus helping to rationalize inhibitor activities observed by the experiment. In summary, the thienobenzo- and naphtho-triazoles tested here are accommodated by cholinesterases through H-bonds involving one of the triazole’s nitrogens, π-π stacking between the aromatic moieties of the ligand, and aromatic residues of the active sites of cholinesterases, as well as π-alkyl interactions.

### 2.4. Crystal Structure of Compound ***5***

**Figure 7:**
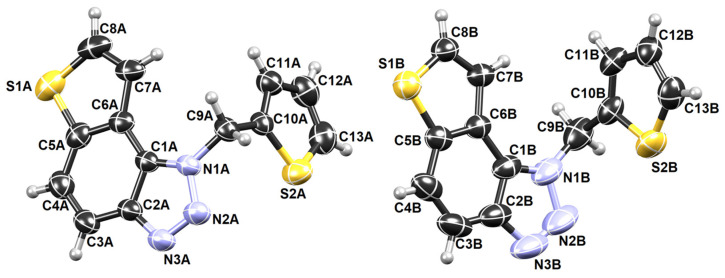
ORTEP-3 diagrams of two symmetry-independent molecules of **5**.

The crystal structure of compound **5** is also successfully determined. It is one of the most active potential BChE inhibitors in this research, which is why information about it is gaining attention. The asymmetric unit of **5** contains two symmetry-independent molecules labeled A and B (Figure 7). 

**Figure 8:**
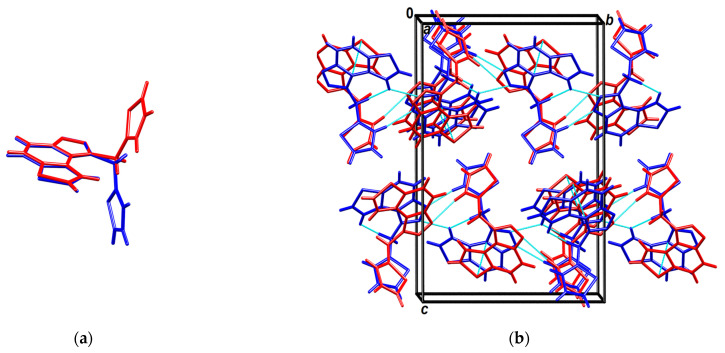
(**a**) Overlay of molecules A (red) and B (blue); (**b**) Crystal packing viewed approximately in the direction of the axis *a*. Molecules A are red, B are blue, and short intermolecular contacts (weak hydrogen bonds and π-stacking) are shown as cyan-dotted lines.

Their conformations are enantiomeric-like (Figure 8a). The molecules stack in columns parallel to the axis *a* in an alternating fashion (…ABAB…) (Figure 8b). The stacks are laterally connected by weak C-H∙∙∙N hydrogen bonds.

## 3. Materials and Methods

### 3.1. General Remarks

Nuclear magnetic resonance (NMR) spectroscopic data for ^1^H and ^13^C nuclei were recorded at room temperature on Bruker Avance 300 and 600 MHz spectrometers. Deuterated chloroform, CDCl_3,_ with tetramethylsilane as standard, was used for recording NMR spectra. Chemical shifts were reported in parts per million. All used solvents were commercially available and were purified by distillation. Anhydrous magnesium sulfate, MgSO_4,_ was used for drying organic layers after extractions. Column chromatography was performed on columns with silica gel (60 Å, technical grade) and by a Biotage Isolera system utilizing a silica column (Interchimthe Si-HC puriFlash, 50 µ) with the appropriate solvent system. The abbreviations used in this experimental procedure were NMR—nuclear magnetic resonance, Cy-hex—cyclohexane, EtOAc—ethyl acetate, PE—petroleum ether, E—diethylether, EtOH—ethanol, MeOH—methanol, DCM—dichloromethane. Preparative photochemical reactions were performed in a closed vessel in a photochemical reactor, Rayonet, equipped with UV lamps of 313 nm. High-resolution mass spectrometry (HRMS) analyses were carried out on a mass spectrometer (MALDI TOF/TOF analyzer) equipped with an Nd:YAG laser operating at 355 nm with a fitting rate of 200 Hz in the positive (H+) or negative (-H) ion reflector mode. All solvents were removed from the solutions by a rotary evaporator under reduced pressure.

### 3.2. General Procedure for the Synthesis of Starting Compounds ***1a**–**6a***

Starting compounds **1a**–**6a** were mixtures of *cis*- and *trans*-isomers of heterostilbenes synthesized by the Wittig reaction. The reaction apparatus was purged with nitrogen for 15 min before adding the reagents. In three-necked round-bottom flasks (100 mL), solutions of the 2-thienyl-phosphonium salt (11 mmol) were dissolved in 50 mL of absolute EtOH (dried on 3 Å sieves). Solutions of sodium ethoxide (11 mmol, 1.1 eq of Na dissolved in 10 mL of absolute ethanol) were added in strictly anhydrous conditions under nitrogen dropwise. Different triazole aldehydes (11 mmol) were added directly to stirred solutions. The reaction mixtures were left to stir for 24 h at room temperature with a nitrogen balloon. After removing the solvent by a rotary evaporator under reduced pressure, the solid reaction mixtures were extracted with toluene p.a. (3 × 25 mL). The organic layers were dried under anhydrous MgSO_4_. Final products as mixtures of *cis*- and *trans*-isomers of **1a**–**6a** were isolated by column chromatography on silica gel using PE/E as an eluent and confirmed by ^1^H NMR spectroscopy and HRMS analyses.

### 3.3. General Procedure for the Synthesis of the Electrocyclization Photoproducts ***1–6***

Mixtures of previously synthesized compounds **1a**–**6a** were dissolved in toluene p.a. (~2.5 × 10^−3^ M) and transferred to a quartz vessel (50 mL) with the addition of a catalytic amount of iodine and irradiated with 10 UV lamps at 313 nm in a Rayonet photochemical reactor for 3–5 h to achieve almost complete conversions. After removing the solvent by a rotary evaporator under reduced pressure, the photoproducts **3**–**6** (for the spectroscopic data of compounds **1** and **2**, see [21]) were purified by column chromatography using PE/E (10%) as eluent from the traces of the starting substrates (in the first fractions) and were completely spectroscopically characterized by NMR and HRMS measurements. 


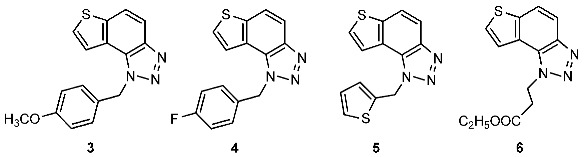


**1**-**(4**-**methoxybenzyl)**-**1*H***-**thieno[3′**,**2′:3**,**4]benzo[1**,**2**-***d*][1,2,3]triazole (3)**: 7 mg (isolated yield 55%); R*_f_* (PE/E (10%)) = 0.53; ^1^H NMR (CDCl_3_, 300 MHz) *δ*/ppm: 7.99 (d, *J* = 9.0 Hz, 1H), 7.80 (d, *J* = 8.8 Hz, 1H), 7.57–7.52 (m, 2H), 7.15 (d, *J* = 8.7 Hz, 2H), 6.83 (d, *J* = 8.7 Hz, 2H), 6.06 (s, 2H), 3.75 (s, 3H); ^13^C NMR (CDCl_3_, 75 MHz) *δ*/ppm: 159.5, 144.7, 139.9, 128.6, 127.9, 127.7, 127.1, 122.7, 120.3, 119.2, 116.1, 114.5, 55.3, 52.6; HRMS (*m*/*z*) for C_16_H_13_N_3_OS (obtained for the pure product): [M + H]^+^_calcd_ = 295.0779, [M + H]^+^_measured_ = 295.0774.

**1**-**(4**-**fluorobenzyl)**-**1*H***-**thieno[3′**,**2′:3**,**4]benzo[1**,**2**-***d*][1,2,3]triazole (4)**: 30 mg (isolated yield 59%); R*_f_* (PE/E (10%)) = 0.27; ^1^H NMR (CDCl_3_, 300 MHz) *δ*/ppm: 8.00 (d, *J* = 8.9 Hz, 1H), 7.82 (d, *J* = 8.8 Hz, 1H), 7.58 (d, *J* = 5.5 Hz, 1H), 7.49 (d, *J* = 5.5 Hz, 1H), 7.21–7.16 (m, 2H), 7.01 (t, *J* = 8.9 Hz, 2H), 6.09 (s, 2H); ^13^C NMR (CDCl_3_, 150 MHz) *δ*/ppm: 162.6 (d, *J*_C-F_ = 250 Hz), 144.7, 140.2, 130.9, 128.6, 128.4, 127.9, 122.6, 119.9, 119.3, 116.2, 116.1, 52.4; HRMS (*m*/*z*) for C_15_H_10_FN_3_S (obtained for the pure product): [M + H]^+^_calcd_ = 283.0580, [M + H]^+^_measured_ = 283.0575.

**1**-**(thiophen**-**2**-**ylmethyl)**-**1*H***-**thieno[3′**,**2′:3**,**4]benzo[1**,**2**-***d*][1,2,3]triazole (5)**: 23 mg (isolated yield 50%); R*_f_* (PE/E (10%)) = 0.32; ^1^H NMR (CDCl_3_, 300 MHz) *δ*/ppm: 7.99 (d, *J* = 8.8 Hz, 1H), 7.82 (d, *J* = 9.2 Hz, 1H), 7.69 (d, *J* = 5.6 Hz, 1H), 7.63 (d, *J* = 5.58 Hz, 1H), 7.24 (dd, *J* = 4.9, 1.2 Hz, 1H), 7.02–7.01 (m, 1H), 6.93 (dd, *J* = 5.0, 3.6 Hz, 1H), 6.27 (s, 2H); ^13^C NMR (CDCl_3_, 150 MHz) *δ*/ppm: 144.6, 140.2, 137.3, 128.3, 127.8, 127.3, 126.5, 126.1, 122.6, 120.1, 119.3, 116.1, 48.4; HRMS (*m*/*z*) for C_13_H_9_N_3_S_2_: [M + H]^+^_calcd_ = 271.0238, [M + H]^+^_measured_ = 271.0236.

**Ethyl 3**-**(1*H***-**thieno[3′**,**2′:3**,**4]benzo[1**,**2**-***d*][1,2,3]triazol**-**1**-**yl)propanoate (6)**: 57 mg (isolated yield 63%); R*_f_* (DCM/EtOAc (20%)) = 0.65; ^1^H NMR (CDCl_3_, 600 MHz) *δ*/ppm: 7.95 (d, *J* = 8.9 Hz, 1H), 7.89 (d, *J* = 5.5 Hz, 1H), 7.83 (d, *J* = 9.1 Hz, 1H), 7.74 (d, *J* = 5.6 Hz, 1H), 5.21 (t, *J* = 7.5 Hz, 2H), 4.18 (q, *J* = 7.2 Hz, 2H), 3.19 (t, *J* = 8.2 Hz, 2H), 1.23 (t, *J* = 7.1 Hz, 3H); ^13^C NMR (CDCl_3_, 150 MHz) *δ*/ppm: 150.3, 128.1, 119.7, 119.2, 116.1, 61.2, 45.1, 34.5, 14.0 (3 singlets are missing); HRMS (*m*/*z*) for C_13_H_13_N_3_S: [M + H]^+^_calcd_ = 275.0728, [M + H]^+^_measured_ = 275.0727.

### 3.4. General Procedure for the Synthesis of the Thienobenzo-Triazoles ***7–12***

4-keto-4,5,6,7-tetrahydrothianaphthene (1 eq) was dissolved in toluene. 1-azido-4-nitro-benzene (1 eq), corresponding amines (1.4 eq), acetic acid (0.3 eq), and 4 Å molecular sieves were added, and reaction mixtures were stirred at 100 °C overnight. Reaction mixtures were cooled to room temperature, and solvents were removed by a rotary evaporator under reduced pressure. Oily products were extracted with EtOAc and water. Organic layers were dried over sodium sulfate, Na_2_SO_4_ filtered, and evaporated until dryness to obtain crude products **13**–**19**. 2,3-dichloro-5,6-dicyano-1,4-benzoquinone (1.2 eq) was added to solutions of thienobenzo-triazoles **13**–**19** (1 eq) in dioxane. Reaction mixtures were stirred at 70 °C. After leaving them overnight, the reaction mixtures were cooled to room temperature and extracted with EtOAc and 1M KOH. Organic layers were dried over Na_2_SO_4_, filtered, and evaporated till dryness to obtain crude products **7**–**12**. Crude products were purified by a system utilizing silica columns (Interchim Si-HC puriFlash, 50 µ) using an appropriate solvent system. Appropriate fractions were combined and evaporated under reduced pressure to obtain the final pure products **7**–**12**.


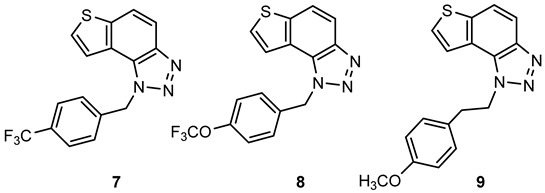


**1**-**(4**-**(trifluoromethyl)benzyl)**-**1*H***-**thieno[3′**,**2′:3**,**4]benzo[1**,**2**-***d*][1,2,3]triazole (7)**: 71 mg (isolated yield 46%); R*_f_* (Cy-hex/EtOAc (50%)) = 0.47; ^1^H NMR (CDCl_3_, 600 MHz) *δ*/ppm: 8.04 (d, *J* = 9.0 Hz, 1H), 7.86 (dd, *J* = 8.9, 0.8 Hz, 1H), 7.61–7.60 (m, 3H), 7.46 (dd, *J* = 5.6, 0.8 Hz, 1H), 7.32 (d, *J* = 8.0 Hz, 2H), 6.21 (s, 2H); ^13^C NMR (CDCl_3_, 150 MHz) *δ*/ppm: 144.9, 140.5, 139.3, 130.8, 128.8, 128.4, 127.1, 126.4, 122.6, 119.8, 119.7, 116.4, 52.7 (the characteristic CF_3_ coupling is not detected due to an insufficient number of scans); HRMS (*m*/*z*) for C_16_H_10_F_3_N_3_S: [M+H]^+^_calcd_ = 333.0548, [M + H]^+^_measured_ = 333.0542.

**1**-**(4**-**(trifluoromethoxy)benzyl)**-**1*H***-**thieno[3′**,**2′:3**,**4]benzo[1**,**2**-***d*][1,2,3]triazole (8)**: 98 mg (isolated yield 59%); R*_f_* (Cy-hex/EtOAc (50%)) = 0.55; ^1^H NMR (CDCl_3_, 600 MHz) *δ*/ppm: 8.03 (d, *J* = 8.9 Hz, 1H), 7.85 (d, *J* = 9.0 Hz, 1H), 7.61 (d, *J* = 5.8 Hz, 1H), 7.51 (d, *J* = 5.5 Hz, 1H), 7.26 (d, *J* = 9.2 Hz, 2H), 7.19 (d, *J* = 8.5 Hz, 2H), 6.15 (s, 2H); ^13^C NMR (CDCl_3_, 150 MHz) *δ*/ppm: 149.3, 144.8, 140.4, 134.0, 128.8, 128.3, 128.3, 127.7, 119.9. 119.6, 116.3, 52.4 (the characteristic CF_3_ coupling is not detected due to an insufficient number of scans); HRMS (*m*/*z*) for C_16_H_10_F_3_N_3_OS: [M + H]^+^_calcd_ = 349.0497, [M + H]^+^_measured_ = 349.0489.

**1**-**(4**-**methoxyphenethyl)**-**1*H***-**thieno[3′**,**2′:3**,**4]benzo[1**,**2**-***d*][1,2,3]triazole (9)**: 106 mg (isolated yield 56%); R*_f_* (Cy-hex/EtOAc (50%)) = 0.43; ^1^H NMR (CDCl_3_, 600 MHz) *δ*/ppm: 7.99 (d, *J* = 9.1 Hz, 1H), 7.84 (d, *J* = 8.7 Hz, 1H), 7.74 (d, *J* = 5.6 Hz, 1H), 7.71 (d, *J* = 5.6 Hz, 1H), 7.13 (d, *J* = 8.2 Hz, 2H), 6.85 (d, *J* = 8.2 Hz, 2H), 5.11 (t, *J* = 7.9 Hz, 2H), 3.79 (s, 3H), 3.33 (t, *J* = 8.0 Hz, 2H); ^13^C NMR (CDCl_3_, 150 MHz) *δ*/ppm: 158.7, 144.3, 139.9, 129.7, 128.9, 127.8, 122.5, 119.4, 118.9, 116.2, 114.2, 101.7, 55.3, 51.1, 35.7; HRMS (*m*/*z*) for C_17_H_15_N_3_OS: [M + H]^+^_calcd_ = 309.0936, [M + H]^+^_measured_ = 309.0928.


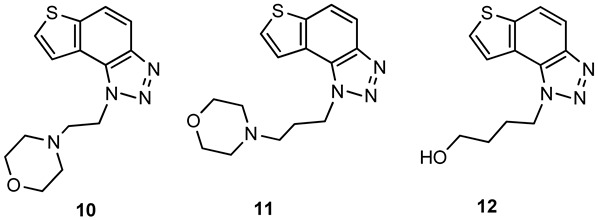


**4**-**(2**-**(1*H***-**thieno[3′**,**2′:3**,**4]benzo[1**,**2**-***d*][1,2,3]triazol**-**1**-**yl)ethyl)morpholine (10)**: 54 mg (isolated yield 20%); R*_f_* (DCM/MeOH (10%)) = 0.64; ^1^H NMR (CDCl_3_, 600 MHz) *δ*/ppm: 7.99 (d, *J* = 8.7 Hz, 1H), 7.84 (d, *J* = 9.1 Hz, 1H), 7.82 (d, *J* = 5.6 Hz, 1H), 7.73 (d, *J* = 5.5 Hz, 1H), 5.06 (t, *J* = 7.1 Hz, 2H), 3.68 (t, *J* = 4.4 Hz, 4H), 3.03 (t, *J* = 7.7 Hz, 2H), 2.58 (t, *J* = 4.6 Hz, 4H); ^13^C NMR (CDCl_3_, 150 MHz) *δ*/ppm: 144.5, 140.2, 128.8, 128.2, 122.8, 119.8, 119.2, 116.4, 66.9, 57.8, 53.9, 47.7; HRMS (*m*/*z*) for C_14_H_16_N_4_OS: [M + H]^+^_calcd_ = 288.1045, [M + H]^+^_measured_ = 288.1040.

**4**-**(3**-**(1*H***-**thieno[3′**,**2′:3**,**4]benzo[1**,**2**-***d*][1,2,3]triazol**-**1**-**yl)propyl)morpholine (11)**: 50 mg (isolated yield 33%); R*_f_* (DCM/MeOH (10%)) = 0.68; ^1^H NMR (CDCl_3_, 600 MHz) *δ*/ppm: 7.99 (d, *J* = 8.9 Hz, 1H), 7.90 (d, *J* = 5.7 Hz, 1H), 7.83 (d, *J* = 9.0 Hz, 1H), 7.71 (d, *J* = 5.7 Hz, 1H), 5.03 (t, *J* = 6.5 Hz, 2H), 3.66 (t, *J* = 4.1 Hz, 4H), 2.44 (t, *J* = 6.5 Hz, 2H), 2.40 (s, 4H), 2.28–2.24 (m, 2H); ^13^C NMR (CDCl_3_, 150 MHz) *δ*/ppm: 144.4, 140.1, 128.9, 127.9, 122.9, 120.1, 119.2, 116.3, 67.1, 55.6, 53.8, 47.8, 27.2; HRMS (*m*/*z*) for C_15_H_18_N_4_OS: [M + H]^+^_calcd_ = 302.1201, [M + H]^+^_measured_ = 302.1199.

**4**-**(1*H***-**thieno[3′**,**2′:3**,**4]benzo[1**,**2**-***d*][1,2,3]triazol**-**1**-**yl)butan**-**1**-**ol (12)**: 34 mg (isolated yield 63%); R*_f_* (DCM/MeOH (10%)) = 0.51; ^1^H NMR (CDCl_3_, 600 MHz) *δ*/ppm: 7.98 (d, *J* = 8.9 Hz, 1H), 7.83 (d, *J* = 8.8 Hz, 1H), 7.81 (d, *J* = 5.6 Hz, 1H), 7.72 (d, *J* = 5.6 Hz, 1H), 4.99 (t, *J* = 7.1 Hz, 2H), 3.75 (t, *J* = 5.7 Hz, 2H), 2.23–2.19 (m, 2H), 1.73–1.69 (m, 2H), 1.67 (s, 1H); ^13^C NMR (CDCl_3_, 150 MHz) *δ*/ppm: 144.6, 140.0, 128.7, 128.2, 122.9, 119.9, 119.2, 116.2, 62.2, 49.6, 29.5, 26.8; HRMS (*m*/*z*) for C_12_H_13_N_3_OS: [M + H]^+^_calcd_ = 247.0779, [M + H]^+^_measured_ = 247.0776.


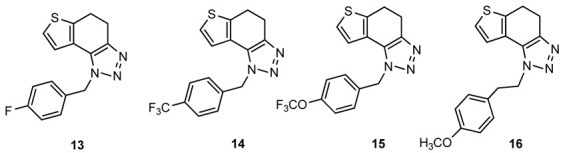


**1**-**(4**-**fluorobenzyl)**-**4**,**5**-**dihydro**-**1*H***-**thieno[3′**,**2′:3**,**4]benzo[1**,**2**-***d*][1,2,3]triazole (13)**: 159 mg (isolated yield 28%); R*_f_* (DCM/EtOAc (20%)) = 0.50; ^1^H NMR (CDCl_3_, 600 MHz) *δ*/ppm: 7.21–7.19 (m, 2H), 7.17 (d, *J* = 5.2 Hz, 1H), 7.06–7.03 (m, 2H), 6.96 (d, *J* = 5.1 Hz, 1H), 5.72 (s, 2H), 3.17 (t, *J* = 2.6 Hz, 4H); ^13^C NMR (CDCl_3_, 150 MHz) *δ*/ppm: 168.8, 166.2, 163.4, 161.8, 141.7, 128.5, 124.4, 121.3, 116.1, 104.9, 51.8, 24.5, 21.3; HRMS (*m*/*z*) for C_15_H_12_FN_3_S: [M + H]^+^_calcd_ = 285.0736, [M + H]^+^_measured_ = 285.0731.

**1**-**(4**-**(trifluoromethyl)benzyl)**-**4**,**5**-**dihydro**-**1*H***-**thieno[3′**,**2′:3**,**4]benzo[1**,**2**-***d*][1,2,3]triazole (14)**: 181 mg (isolated yield 27%); R*_f_* (DCM/EtOAc (20%)) = 0.54; ^1^H NMR (CDCl_3_, 600 MHz) *δ*/ppm: 7.63 (d, *J* = 8.6 Hz, 2H), 7.32 (d, *J* = 8.6 Hz, 2H), 7.18 (d, *J* = 5.2 Hz, 1H), 6.92 (d, *J* = 5.3 Hz, 1H), 5.81 (s, 2H), 3.21–3.15 (m, 4H); ^13^C NMR (CDCl_3_, 150 MHz) *δ*/ppm: 162.2, 157.9, 152.2, 127.1, 126.3, 124.8, 124.6, 121.6, 121.2, 100.6, 52.2, 24.7, 21.5; HRMS (*m*/*z*) for C_16_H_12_F_3_N_3_S: [M + H]^+^_calcd_ = 335.0704, [M + H]^+^_measured_ = 335.0695.

**1**-**(4**-**(trifluoromethoxy)benzyl)**-**4**,**5**-**dihydro**-**1*H***-**thieno[3′**,**2′:3**,**4]benzo[1**,**2**-***d*][1,2,3]triazole (15)**: 100 mg (isolated yield 43%); R*_f_* (DCM/EtOAc (20%)) = 0.53; ^1^H NMR (CDCl_3_, 600 MHz) *δ*/ppm: 7.26 (d, *J* = 9.4 Hz, 2H), 7.22 (d, *J* = 8.4 Hz, 2H), 7.19 (d, *J* = 5.3 Hz, 1H), 6.96 (d, *J* = 5.3 Hz, 1H), 5.75 (s, 2H), 3.18 (t, *J* = 1.9 Hz, 4H); ^13^C NMR (CDCl_3_, 150 MHz) *δ*/ppm: 165.7, 143.1, 133.7, 128.2, 124.5, 124.4, 123.1,121.5, 121.3, 121.1, 103.2, 51.8, 24.6, 21.3; HRMS (*m*/*z*) for C_16_H_12_F_3_N_3_OS: [M + H]^+^_calcd_ = 351.0653, [M + H]^+^_measured_ = 351.0647.

**1**-**(4**-**(trifluoromethoxy)benzyl)**-**4**,**5**-**dihydro**-**1*H***-**thieno[3′**,**2′:3**,**4]benzo[1**,**2**-***d*][1,2,3]triazole (16)**: 252 mg (isolated yield 41%); R*_f_* (DCM/EtOAc (20%)) = 0.43; ^1^H NMR (CDCl_3_, 600 MHz) *δ*/ppm: 7.26 (d, *J* = 4.9 Hz, 1H), 7.13 (d, *J* = 5.1 Hz, 1H), 7.10 (d, *J* = 8.3 Hz, 2H), 6.84 (d, *J* = 8.5 Hz, 2H), 4.69 (t, *J* = 7.8 Hz, 2H), 3.79 (s, 3H), 3.21 (t, *J* = 7.8 Hz, 2H), 3.14 (s, 4H); ^13^C NMR (CDCl_3_, 150 MHz) *δ*/ppm: 158.8, 142.7, 139.4, 129.9, 129.1, 125.1, 124.5, 121.0, 117.7, 114.4, 55,4, 50.9, 36.1, 24.8, 21.4; HRMS (*m*/*z*) for C_17_H_17_N_3_OS: [M + H]^+^_calcd_ = 311.1092, [M + H]^+^_measured_ = 311.1090.


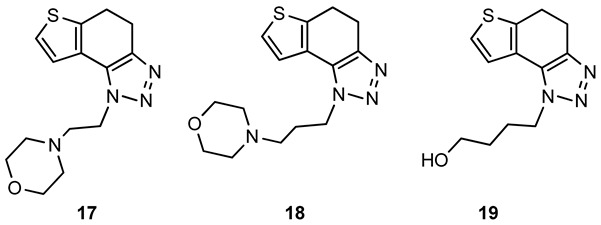


**4**-**(2**-**(4**,**5**-**dihydro**-**1*H***-**thieno[3′**,**2′:3**,**4]benzo[1**,**2**-***d*][1,2,3]triazol**-**1**-**yl)ethyl)morpholine (17)**: 328 mg (isolated yield 57%); R*_f_* (DCM/MeOH (10%)) = 0.64; ^1^H NMR (CDCl_3_, 600 MHz) *δ*/ppm: 7.29–7.27 (m, 2H), 4.65 (t, *J* = 7.4 Hz, 2H), 3.67 (t, *J* = 4.5 Hz, 4H), 3.18–3.12 (m, 4H), 2.91 (t, *J* = 7.4 Hz, 2H), 2.53 (t, *J* = 4.7 Hz, 4H); ^13^C NMR (CDCl_3_, 150 MHz) *δ*/ppm: 142.6, 139.6, 125.2, 124.6, 121.2, 113.9, 67.0, 58.0, 53.9, 47.2, 24.8, 21.5; HRMS (*m*/*z*) for C_14_H_18_N_4_OS: [M + H]^+^_calcd_ = 290.1201, [M + H]^+^_measured_ = 290.1197.

**4**-**(3**-**(4**,**5**-**dihydro**-**1*H***-**thieno[3′**,**2′:3**,**4]benzo[1**,**2**-***d*][1,2,3]triazol**-**1**-**yl)propyl)morpholine (18)**: 224 mg (isolated yield 45%); R*_f_* (DCM/MeOH (10%)) = 0.63; ^1^H NMR (CDCl_3_, 600 MHz) *δ*/ppm: 7.36 (d, *J* = 5.7 Hz, 1H), 7.27 (d, *J* = 5.2 Hz, 1H), 4.62 (t, *J* = 7.6 Hz, 2H), 3.69 (t, *J* = 4.9 Hz, 4H), 3.18–3.12 (m, 4H), 2.42–2.40 (m, 6H), 2.16–2.11 (m, 2H); ^13^C NMR (CDCl_3_, 150 MHz) *δ*/ppm: 139.3, 128.6, 125.0, 124.2, 121.3, 112.3, 66.9, 55.2, 53.6, 46.9, 26.9, 24.6, 21.2; HRMS (*m*/*z*) for C_15_H_20_N_4_OS: [M + H]^+^_calcd_ = 304.1358, [M + H]^+^_measured_ = 304.1356.

**4**-**(4**,**5**-**dihydro**-**1*H***-**thieno[3′**,**2′:3**,**4]benzo[1**,**2**-***d*][1,2,3]triazol**-**1**-**yl)butan**-**1**-**ol (19)**: 172 mg (isolated yield 35%); R*_f_* (DCM/MeOH (10%)) = 0.54; ^1^H NMR (CDCl_3_, 600 MHz) *δ*/ppm: 7.29 (d, *J* = 5.5 Hz, 1H), 7.25 (d, *J* = 5.4 Hz, 1H), 4.60 (t, *J* = 6.6 Hz, 2H), 3.73 (t, *J* = 6.6 Hz, 2H), 3.18–3.12 (m, 4H), 2.11–2.06 (m, 2H), 1.71–1.66 (m, 2H), 1.61 (s, 1H); ^13^C NMR (CDCl_3_, 150 MHz) *δ*/ppm: 142.6, 139.5, 125.1, 124.7, 121.3, 116.6, 62.1, 49.1, 29.5, 26.8, 24.7, 21.4; HRMS (*m*/*z*) for C_12_H_15_N_3_OS: [M + H]^+^_calcd_ = 249.0936, [M + H]^+^_measured_ = 249.0935.

### 3.5. Cholinesterase Inhibition Activity Measurements

AChE and BChE inhibition was determined using a modified spectrophotometric Ellman’s method [34]. Acetylthiocholine iodide (ATChI), S-butyrylthiocholine iodide (BTChI), AChE (EC 3.1.1.7, *Electrophorus electricus*), BChE (EC 3.1.1.8, equine serum) and Trisma base were purchased from Sigma-Aldrich (St. Louis, MO, USA), while Ellman’s reagent 5,50-dithiobis-(2-nitrobenzoic acid) (DTNB) was purchased from Zwijndrecht (Antwerpen, Belgium). The reaction mixture contained 180 µL Tris-HCl buffer (50 mM, pH 8.0), 10 µL of enzyme prepared in 20 mM Tris-HCl buffer, pH 7.5 (final concentration 0.03 U/mL), and 10 µL of tested solution (final concentrations 10–500 μM in ethanol, depending on solubility). The assayed solution of the test compound was pre-incubated for 5 min at 4 °C. The incubation time of 5 min is the exposure time of the enzyme to the inhibitor. The sensitivity of the in vitro assay can be improved by increasing the contact time between the enzyme and the inhibitor. It is carried out at a low temperature and in the dark so that there are no physico-chemical changes in the inhibitors or enzymes. The reaction started with adding 10 μL of DTNB (final concentration 0.3 mM prepared in Tris-HCl buffer) and 10 μL of ATChI/BTChI (final concentration of 0.5 mM prepared in Tris-HCl buffer). The developing yellow color was measured at 405 nm over 6 min at room temperature using a 96-well microplate reader (IRE 96, SFRI Medical Diagnostics). The experiment was run in triplicate. The percentage of enzyme inhibition was calculated from measured data according to the equation: Inhibition (%) = [(A_c_ − A_T_)/A_C_]∙100, where A_C_ is the enzyme activity without the test sample and A_T_ is the enzyme activity with the test sample, calculated as mean values ± standard deviation. In the control measurement, the tested compound was replaced by a buffer solution. Non-enzymatic hydrolysis was measured as blank for each measurement. The IC_50_ value was calculated by a nonlinear fit of compound concentration (log) values vs. response.

### 3.6. Computational Details

Geometry optimizations of the selected ligands were obtained at the M06-2X/6-31G(d) level of theory using the Gaussian16 program package [35] and then utilized for molecular docking. Molecular docking was performed using the Autodock program package [36], with the crystal structure 1EEA.pdb [37] for AChE taken from the Protein Data Bank and 1P0I.pdb [38] for BChE. The quaternary structure of 1EEA.pdb corresponds well to the tetramer of AChE found in electric eel; however, the amino acid sequence is taken from the *Torpedo californica* AChE, as mentioned in Section 2.3. The docking results were obtained using the Lamarckian Genetic Algorithm, with 25 requested genetic algorithm dockings with 25 binding poses for each ligand. The residues of the enzymes were kept rigid during the docking.

### 3.7. X-ray Diffraction

Single crystal X-ray diffraction data were collected on a dual source (Mo/Cu) Rigaku Oxford Diffraction Synergy S diffractometer equipped with an Oxford Cryosystems Series 800 cryostat. The program package CrysAlis PRO [39] was used for data reduction and numerical absorption correction.

The structure was solved using SHELXS97 [40] and refined with SHELXL-2017 [41]. Models were refined using the full-matrix least-squares refinement; all non-hydrogen atoms were refined anisotropically. Hydrogen atoms were located in a difference Fourier map and refined as riding entities.

Molecular geometry calculations were performed by PLATON [42], and molecular graphics were prepared using ORTEP-3 [43] and Mercury [44]. Crystallographic and refinement data for structure **5** reported in this paper are shown in Table S2 in Electronic Supplement Information.

## 4. Conclusions

The synthesis of 19 new thienobenzo/naphtho-triazoles **1**–**19** by two different approaches improved synthetic productivity. The final result was a more extensive set of molecules to test with different functionalities on the triazole ring or differing in the aromatic character of the central ring in the structure. As predicted, most prepared molecules showed better inhibition of the enzyme butyrylcholinesterase, considering that the new molecules **1**–**19** were designed according to the previous results. Seven compounds show inhibitory activity towards BChE in the range of values characteristic of common cholinesterase inhibitors, although their inhibitory potency varied with compound functionalities. Therefore, for the future design of cholinesterase inhibitors and the search for therapeutics for neurological disorders, compounds with a thienobenzo-triazole skeleton, a *para*-OCH_3_-benzyl group on it, or the *para*-OCH_3_-phenyl group on the prolonged aliphatic chain should be interesting. Furthermore, the *para*-F-benzyl group attached to the triazole with the dihydro or aromatic central ring, and the basic skeleton with an ethoxy group or additional thiophene ring, should be considered in the design of new BChE inhibitors.

## Figures and Tables

**Table 1:** Calculated IC_50_ values for compounds **1**–**19**.

Compound ^1^	Structure	IC_50_ (μM) AChE	IC_50_ (μM) BChE	Compound	Structure	IC_50_ (μM) AChE	IC_50_ (μM) BChE
**1**	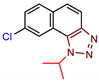	59.9	39.6	**12**	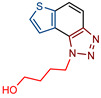	-	106.7
**3**	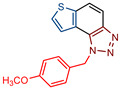	116.0	25.5	**13**	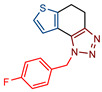	-	58.0
**4**	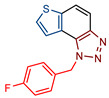	97.0	37.5	**14**	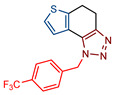	117.6	>150
**5**	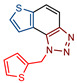	118.6	23.4	**15**	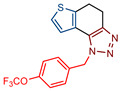	88.7	-
**6**	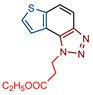	-	39.6	**16**	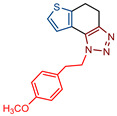	145.3	119.4
**7**	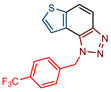	139.6	>150	**17**	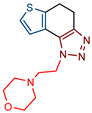	45.6	-
**8**	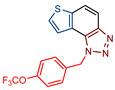	68.0	213.5	**19**	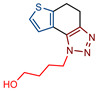	-	74.1
**9**	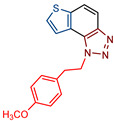	-	18.6	**Galantamine**		0.15	7.9

^1^ Compounds **2**, **10**, **11**, and **18** were inactive toward both cholinesterases.

## Data Availability

Additional data is available on request.

## Supplementary Materials

The supporting information can be downloaded at https://www.mdpi.com/article/10.3390/ijms24065879/s1.
