# Supplementary material for: New Thienobenzo/Naphtho-Triazoles as Butyrylcholinesterase Inhibitors: Design, Synthesis and Computational Study
Source: Int J Mol Sci. 2023 Mar 20;24(6):5879. doi: 10.3390/ijms24065879 (PMC10059756; doi:10.3390/ijms24065879)
Supplement: Supplementary file 1 [file ijms-24-05879-s001.zip › ijms-2279595-supplementary.pdf]

Electronic Supporting Information

New Thienobenzo/Naphtho-Triazoles as Butyrylcholinesterase Inhibitors. Design, Synthesis and Computational Study

Milena Mlakić [1,†], Ida Selec [1,2,†], Irena Ćaleta [2,*], Ilijana Odak [3], Danijela Barić [4], Ana Ratković [2], Krešimir Molčanov [5] and Irena Škorić [1,*]

1 Department of Organic Chemistry, Faculty of Chemical Engineering and Technology, University of Zagreb, Marulićev trg 19, HR-10000 Zagreb, Croatia
2 Chemistry, Selvita Ltd., Prilaz Baruna Filipovića 29, HR-10000 Zagreb, Croatia
3 Department of Chemistry, Faculty of Science and Education, University of Mostar, Matice hrvatske bb, 88000 Mostar, Bosnia and Herzegovina
4 Group for Computational Life Sciences, Division of Physical Chemistry, Ruđer Bošković Institute, Bijenička cesta 54, HR-10000 Zagreb, Croatia
5 Division of Physical Chemistry, Ruđer Bošković Institute, Bijenička cesta 54, HR-10000 Zagreb, Croatia
* Correspondence: iskoric@fkit.hr (I.Š.), Tel.: +385-1-4597 241; irena.caleta@selvita.com (I.Ć.), Tel.: +385-1-888 63 87
† These authors contributed equally to this work.

<u>Content:</u>

1. ^{1}H and ^{13}C NMR spectra of synthesized compounds (Figs S1–S62)

2. MS spectra and HRMS analyses of synthesized compounds (Figs S63–S79)

3. Cartesian coordinates of ligands docked into AChE and BChE

4. Estimated free energies of binding obtained by molecular docking (Table S1)

5. Crystallographic and refinement data for the structure **5** (Table S2)

6. Reorganized experimental results on the inhibitory activity of 1-19 (Table S3)

1. ¹H and ¹³C NMR spectra of synthesized compounds

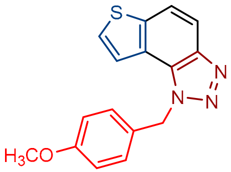

3

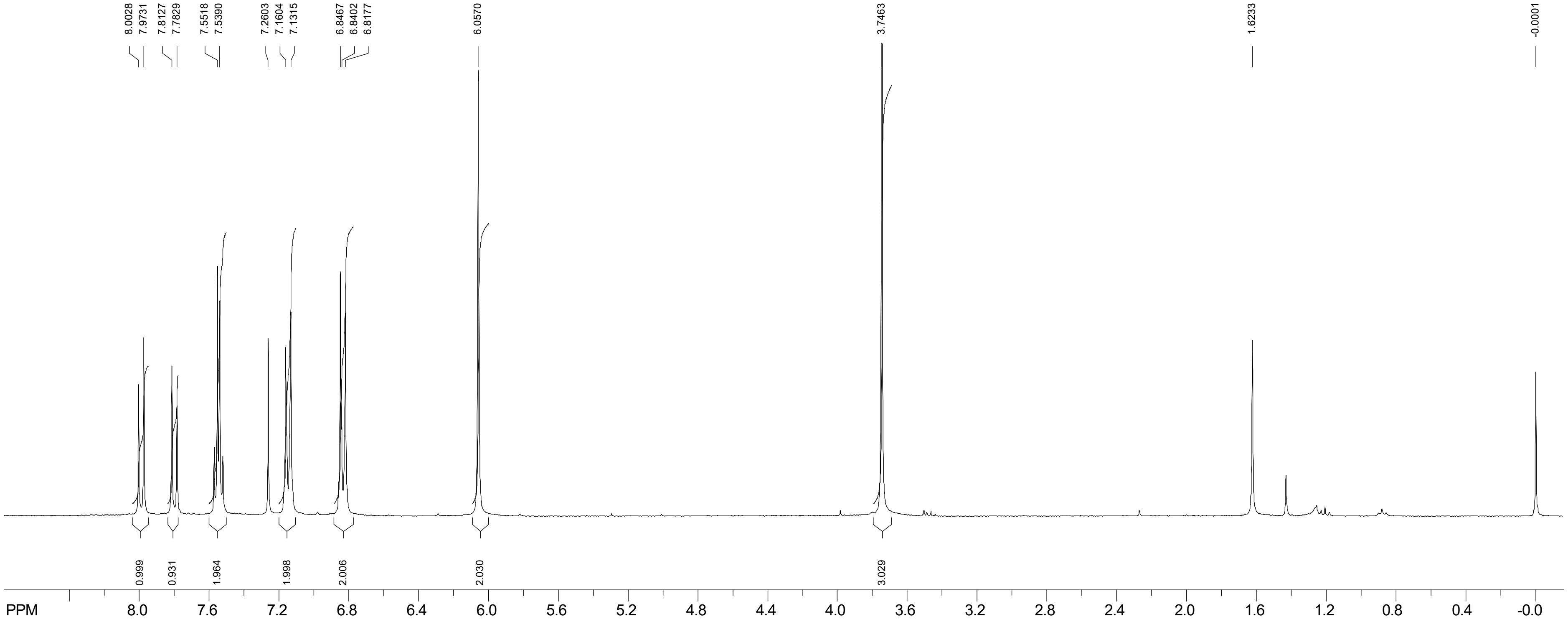

file: C:\Users\Irena\Desktop\IJMS II 2023\spektri Milena, spojevi 3-6 i poèetni\MD-303(4) p_OMe hv\fid expt: <zg30>
transmitter freq.: 300.132701 MHz
time domain size: 32768 points
width: 6172.84 Hz = 20.567034 ppm = 0.188380 Hz/pt
number of scans: 64

freq. of 0 ppm: 300.130006 MHz
processed size: 32768 complex points
LB: 0.000 GB: 0.0000

Figure S1. ¹H NMR spectrum ($CDCl_3$) of compound **3**.

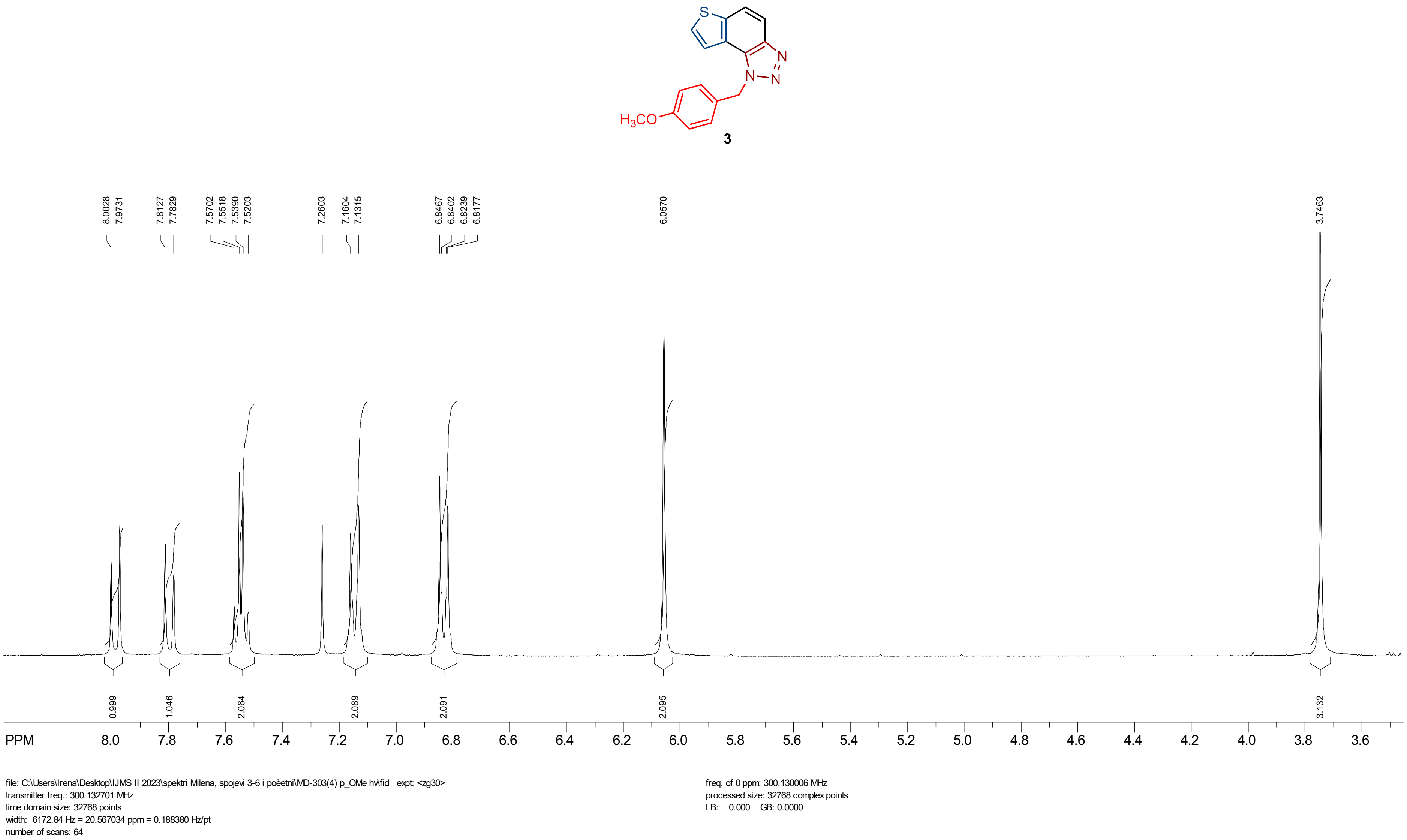

Figure S2. Part of the ^{1}H NMR spectrum ($CDCl_3$) of compound **3**.

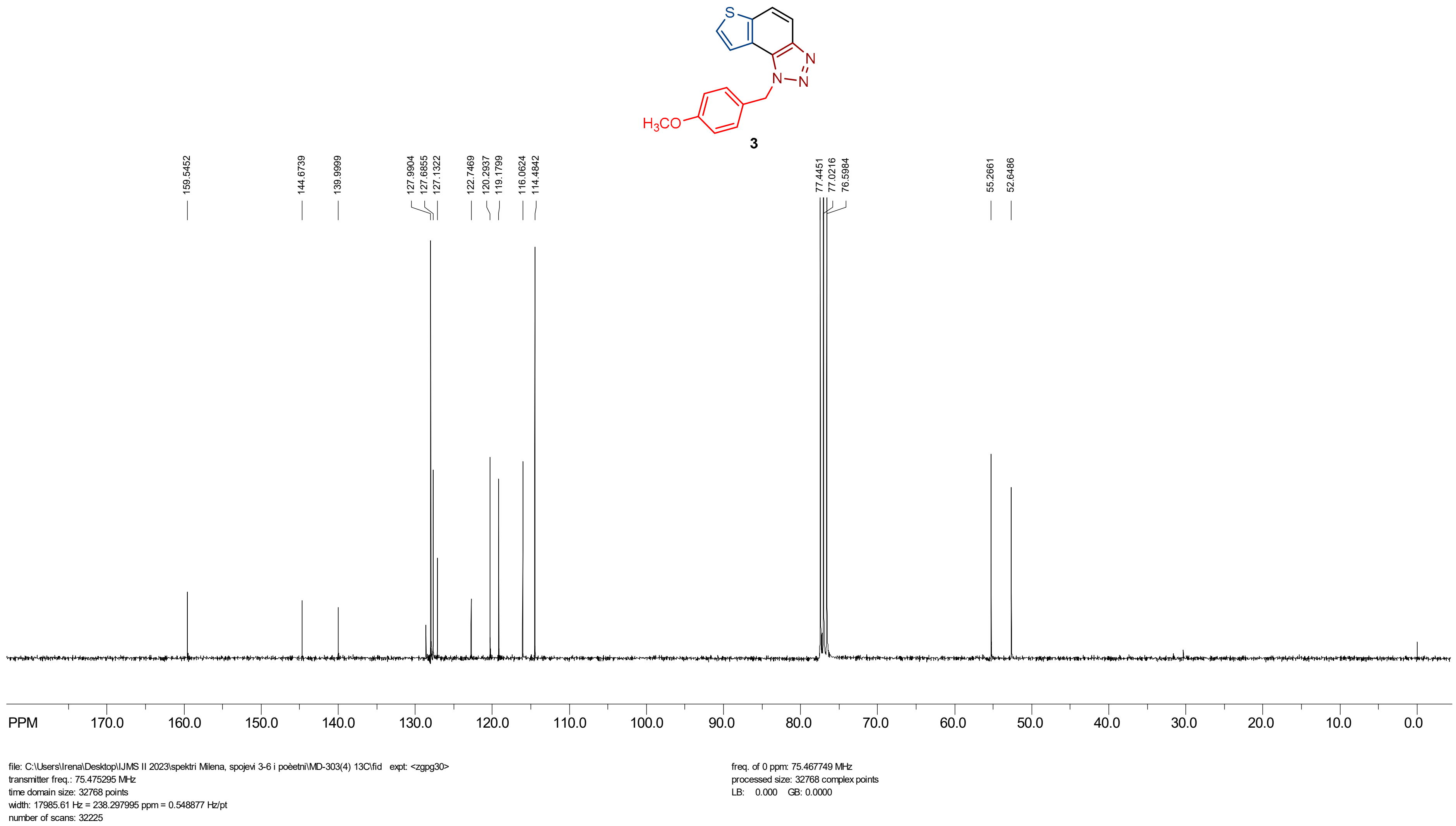

Figure S3. ^{1}H NMR spectrum ($CDCl_3$) of compound **3**.

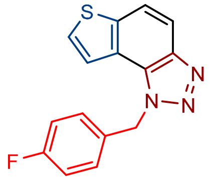

4

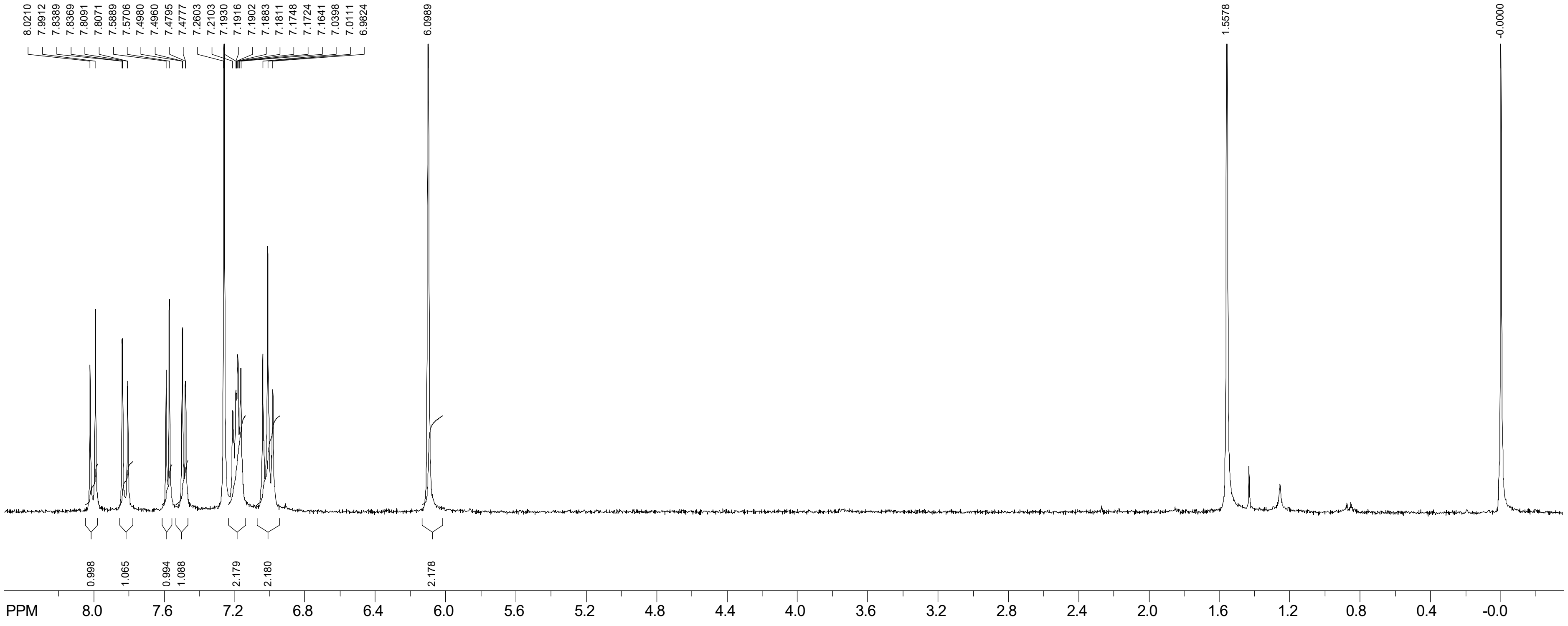

file: C:\Users\Irena\Desktop\IJMS II 2023\spektri Milena, spojevi 3-6 i poèetni\EĐ-28(9) p_F hv\fid   expt: <zg30>
transmitter freq.: 300.132701 MHz
time domain size: 32768 points
width: 6172.84 Hz = 20.567034 ppm = 0.188380 Hz/pt
number of scans: 64

freq. of 0 ppm: 300.130006 MHz
processed size: 32768 complex points
LB: 0.000 GB: 0.0000

Figure S4. ^{1}H NMR spectrum ($CDCl_3$) of compound **4**.

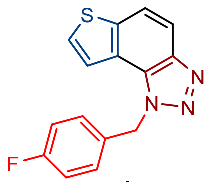

4

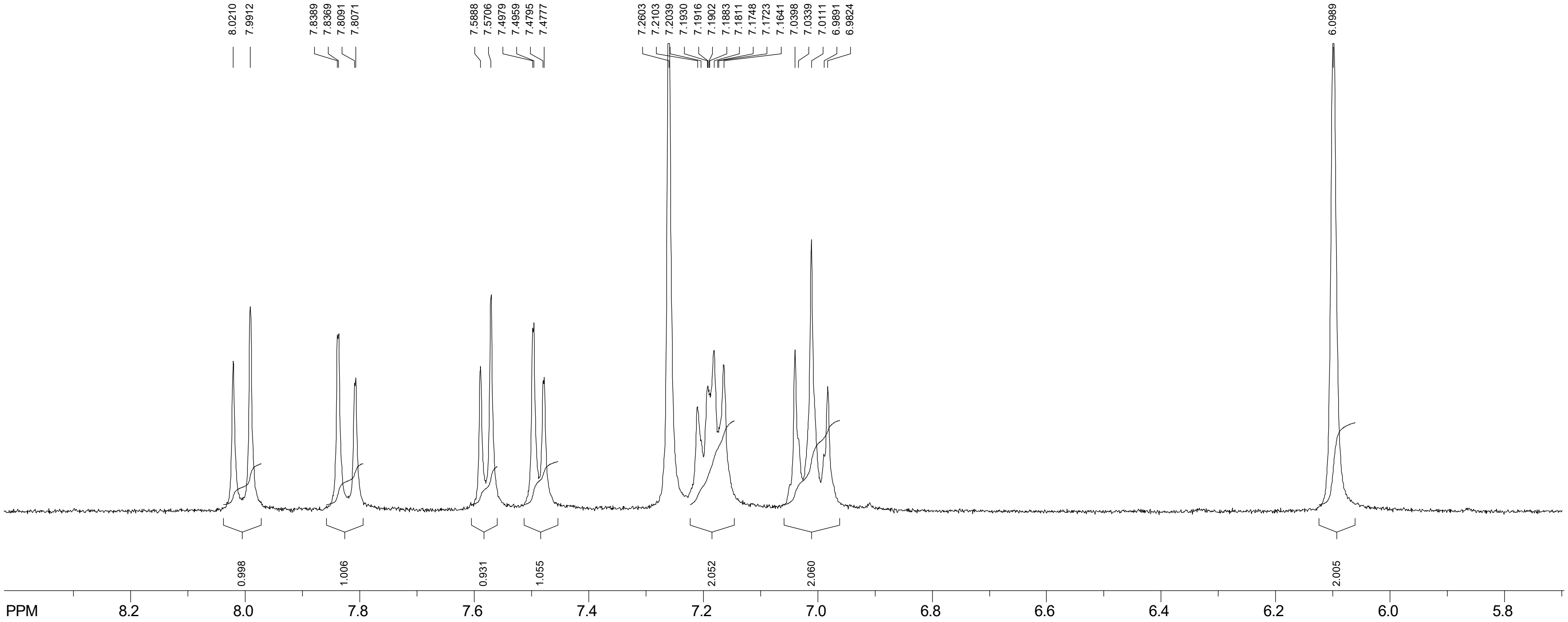

file: C:\Users\Irena\Desktop\IJMS II 2023\spektri Milena, spojevi 3-6 i poèetni\EĐ-28(9) p_F hv\fid   expt: <zg30>
transmitter freq.: 300.132701 MHz
time domain size: 32768 points
width: 6172.84 Hz = 20.567034 ppm = 0.188380 Hz/pt
number of scans: 64

freq. of 0 ppm: 300.130006 MHz
processed size: 32768 complex points
LB: 0.000 GB: 0.0000

Figure S5. Part of the ^{1}H NMR spectrum ($CDCl_3$) of compound **4**.

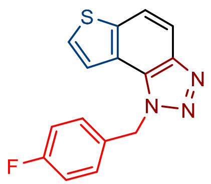

4

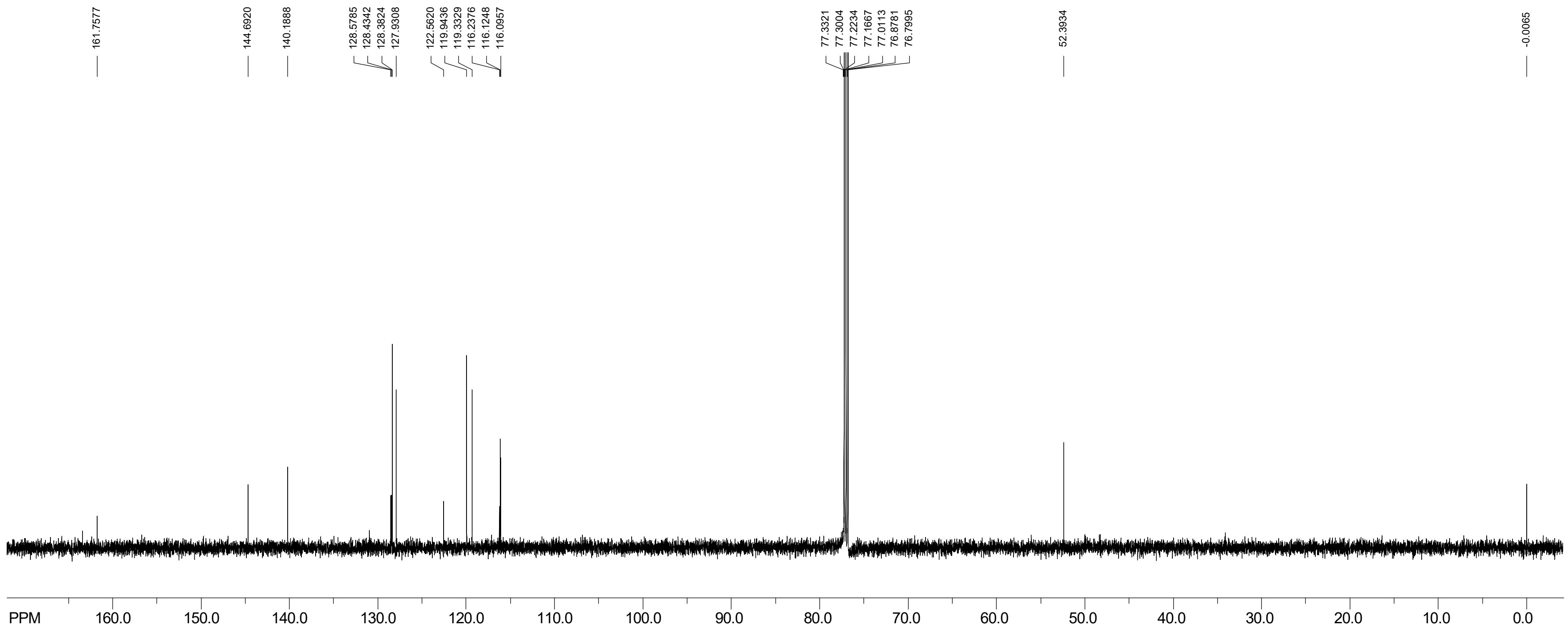

file: C:\Users\Irena\Desktop\IJMS II 2023\spektri Milena, spojevi 3-6 i poèetni\EĐ-28(9) COM\fid expt: <zgpg30>
transmitter freq.: 150.917899 MHz
time domain size: 65536 points
width: 35971.22 Hz = 238.349615 ppm = 0.548877 Hz/pt
number of scans: 2104

freq. of 0 ppm: 150.902809 MHz
processed size: 32768 complex points
LB: 0.000 GB: 0.0000

Figure S6. ^{13}C NMR spectrum ($CDCl_3$) of compound **4**.

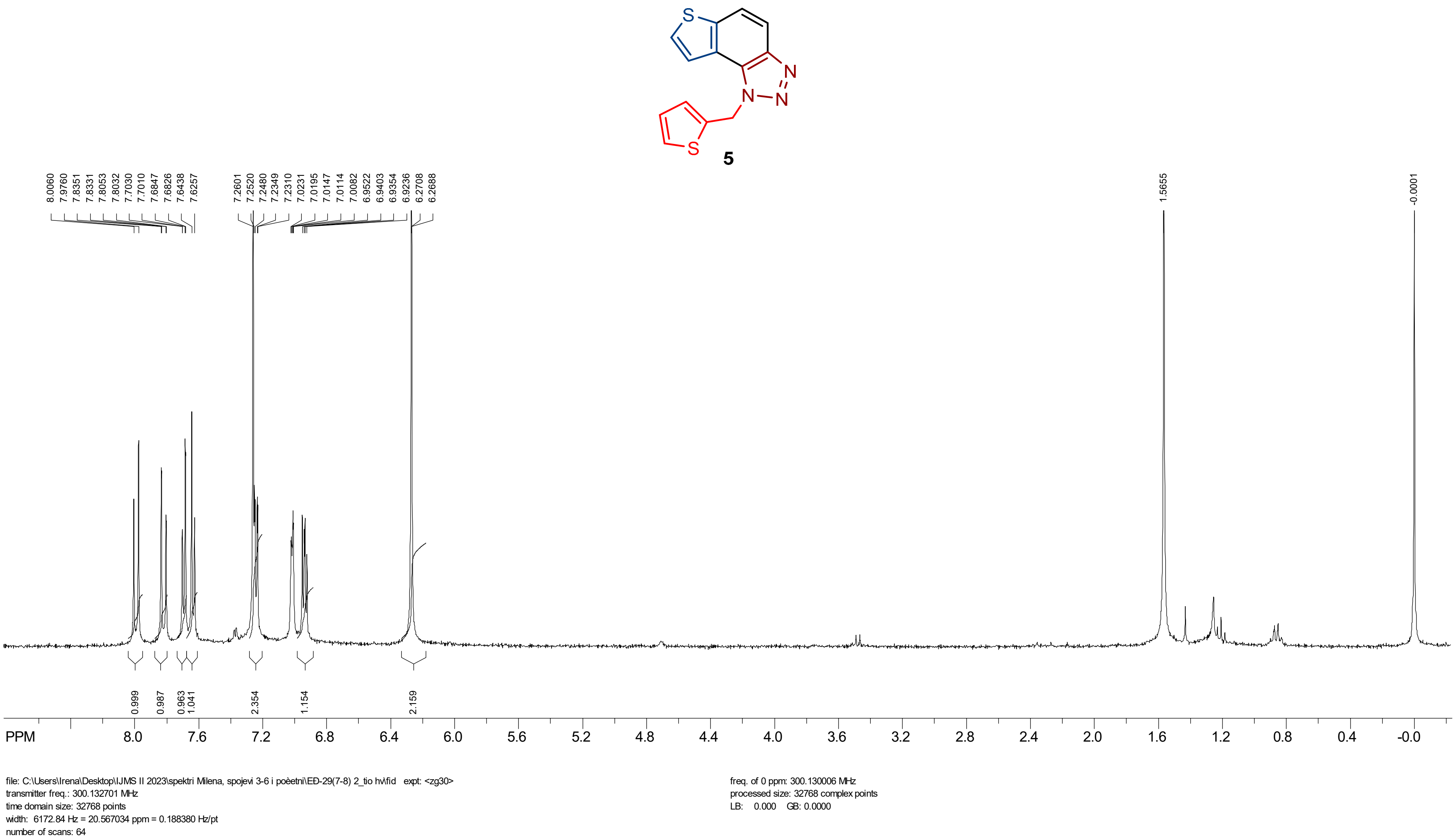

Figure S7. ^{1}H NMR spectrum ($CDCl_3$) of compound **5**.

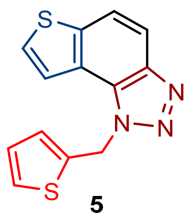

5

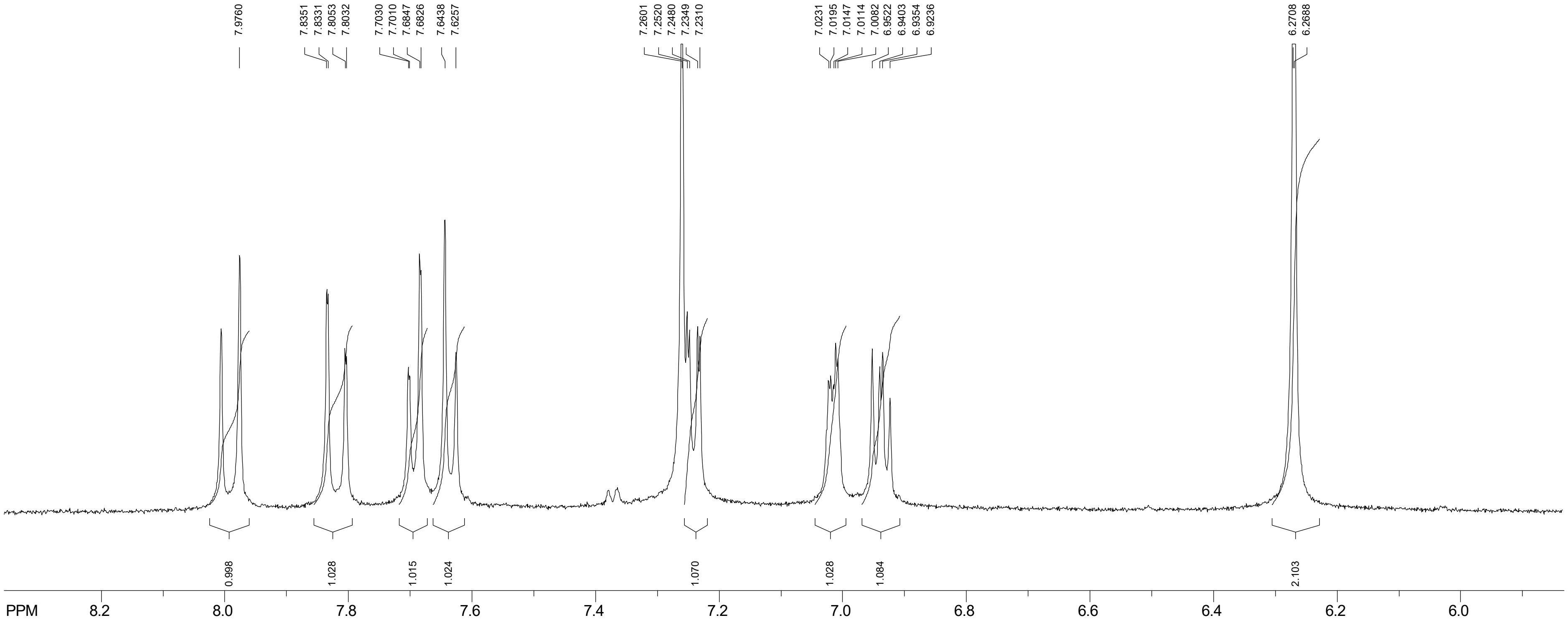

file: C:\Users\Irena\Desktop\IJMS II 2023\spektri Milena, spojevi 3-6 i poèetni\EÐ-29(7-8) 2_tio hv\fid expt: <zg30>
transmitter freq.: 300.132701 MHz
time domain size: 32768 points
width: 6172.84 Hz = 20.567034 ppm = 0.188380 Hz/pt
number of scans: 64

freq. of 0 ppm: 300.130006 MHz
processed size: 32768 complex points
LB: 0.000 GB: 0.0000

Figure S8. Part of the ^{1}H NMR spectrum ($CDCl_3$) of compound **5**.

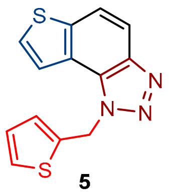

5

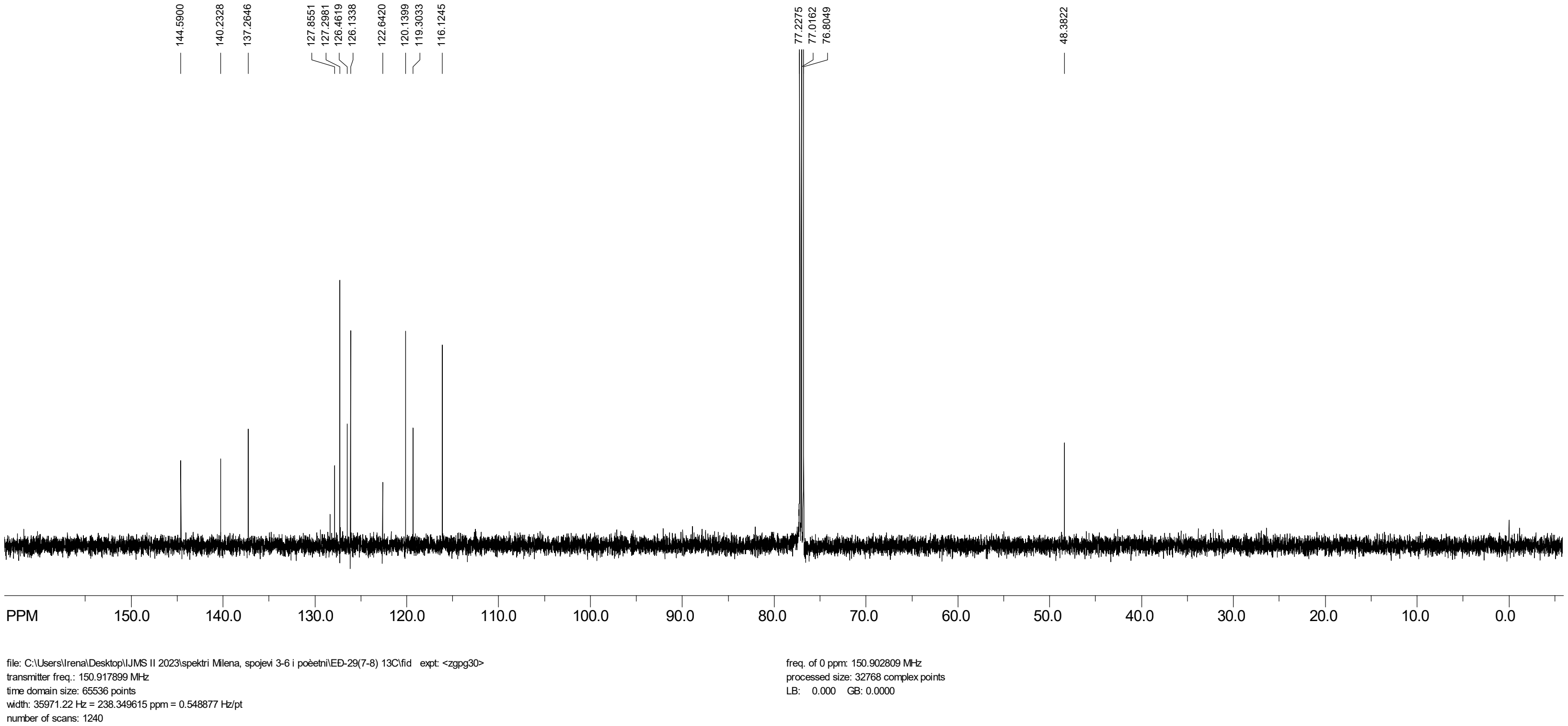

Figure S9. ^{13}C NMR spectrum ($CDCl_3$) of compound **5**.

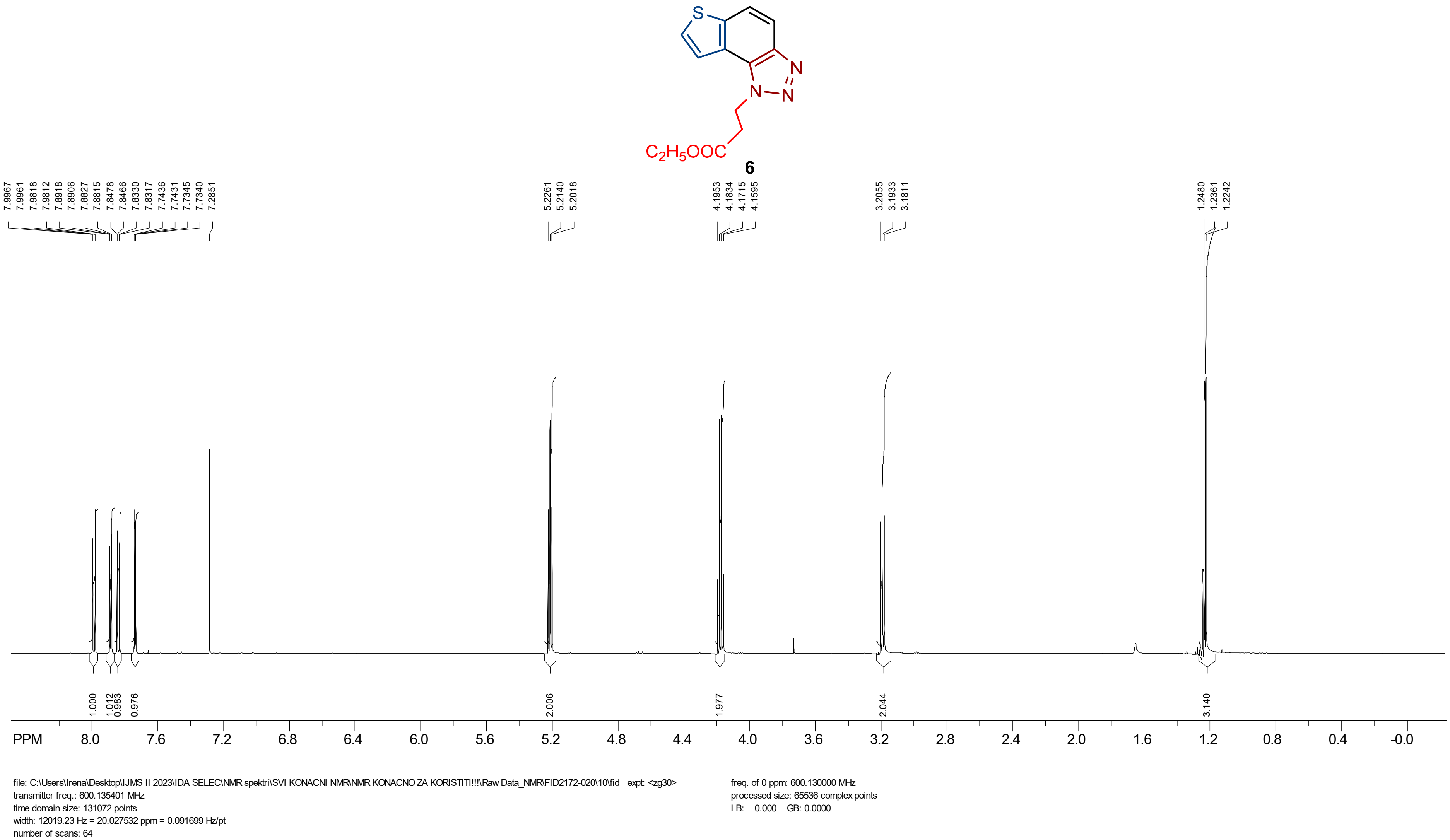

Figure S10. ^{1}H NMR spectrum ($CDCl_3$) of compound **6**.

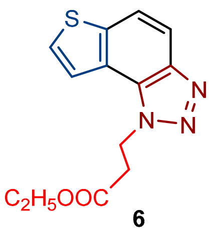

6

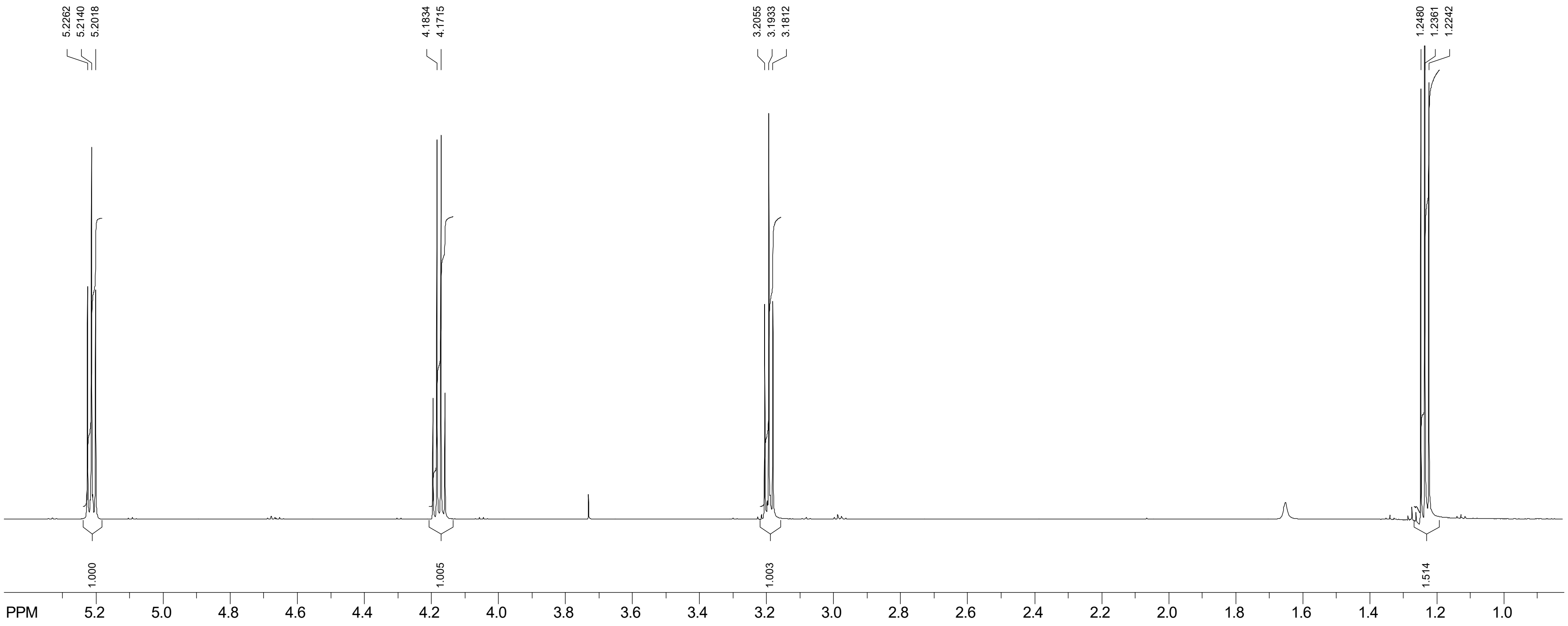

file: C:\Users\Irena\Desktop\IDA SELEC\NMR spektri\SVI KONACNI NMR\Raw data_NMR\icaleta-2023-02-01_27-ZG6 spoj 20P4 1H, 13C, COSY\10\fid expt: <zg30>
transmitter freq.: 600.135401 MHz
time domain size: 131072 points
width: 12019.23 Hz = 20.027532 ppm = 0.091699 Hz/pt
number of scans: 64

freq. of 0 ppm: 600.130000 MHz
processed size: 65536 complex points
LB: 0.000 GB: 0.0000

Figure S11. Aliphatic part of the ^{1}H NMR spectrum ($CDCl_3$) of compound **6**.

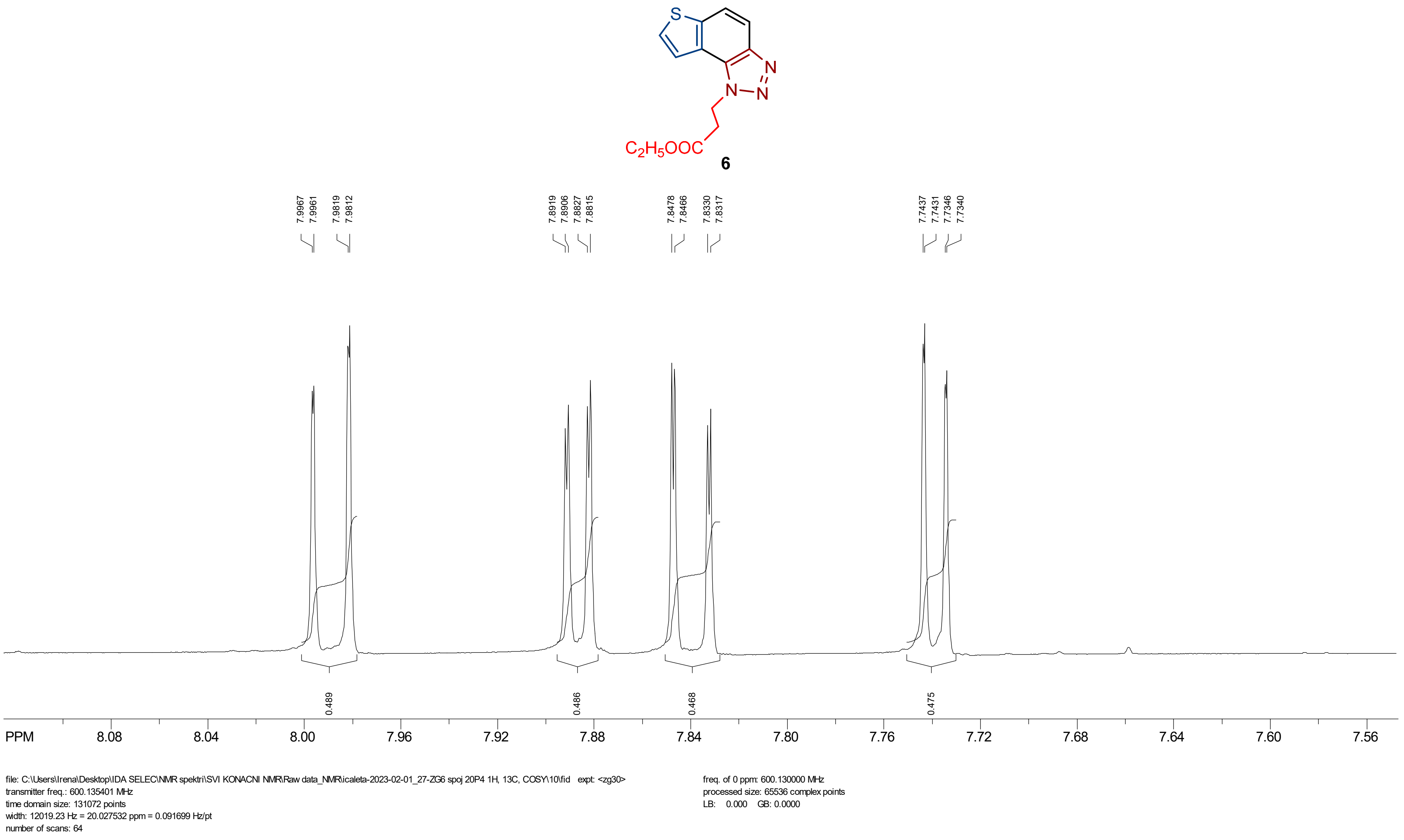

Figure S12. Aromatic part of the ^1H NMR spectrum ($CDCl_3$) of compound **6**.

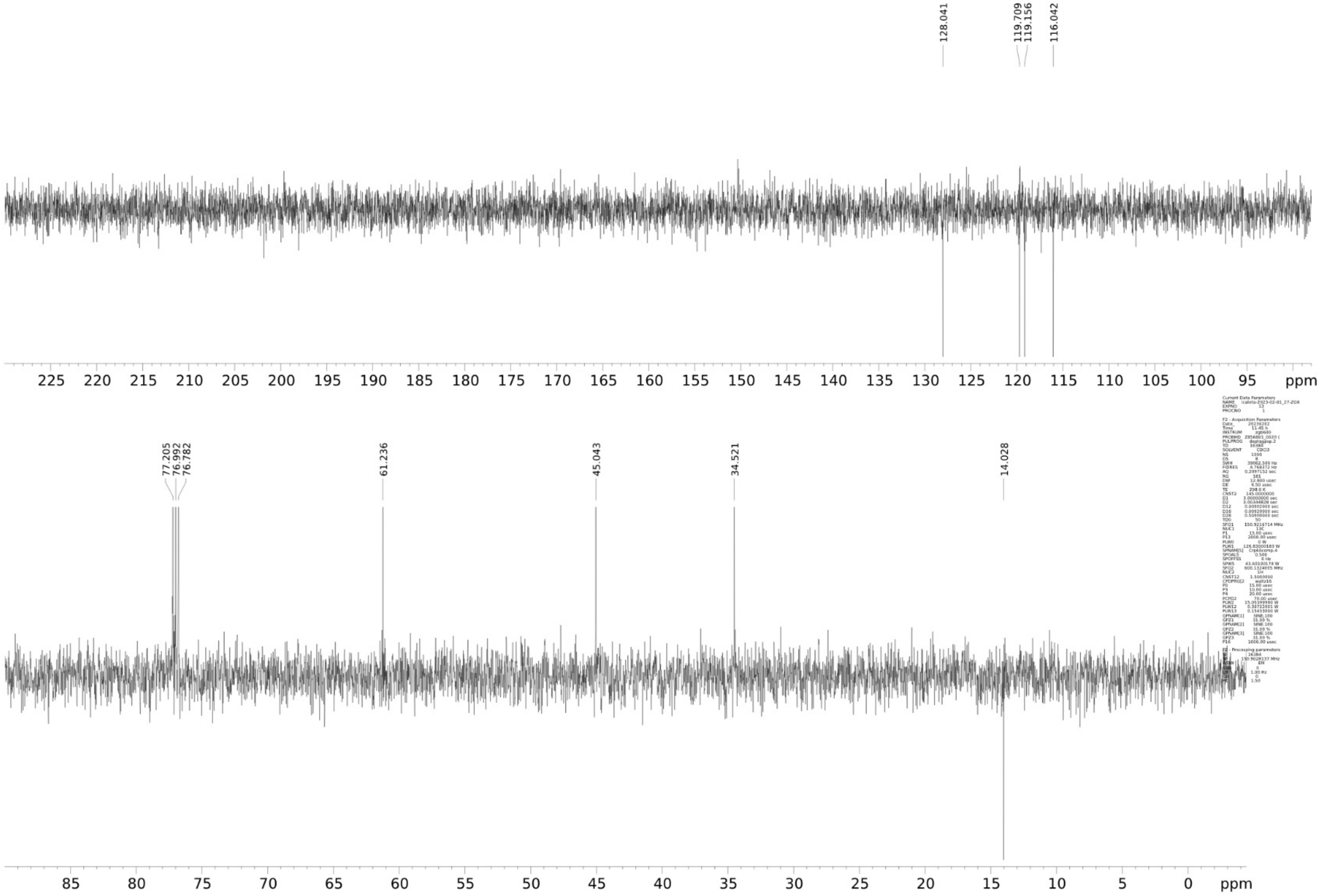

Figure S13. ^{13}C NMR spectrum ($CDCl_3$) of compound **6**.

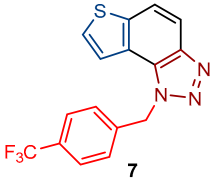

7

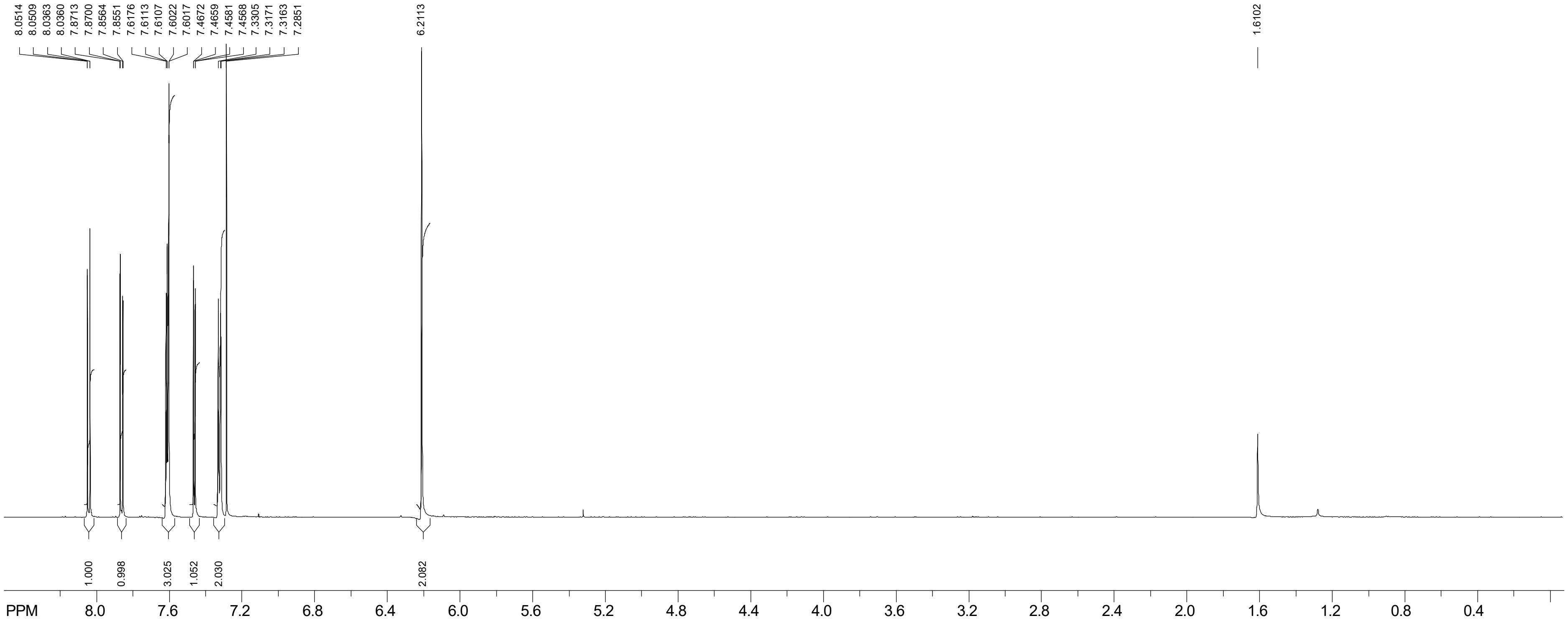

file: C:\Users\Irena\Desktop\IJMS II 2023\IDA SELEC\NMR spektri\SVI KONACNI NMR\NMR KONACNO ZA KORISTITI!!!\Raw Data_NMR\FID2172-048\10\fid expt: <zg30>
transmitter freq.: 600.135401 MHz
time domain size: 131072 points
width: 12019.23 Hz = 20.027532 ppm = 0.091699 Hz/pt
number of scans: 64

freq. of 0 ppm: 600.130000 MHz
processed size: 65536 complex points
LB: 0.000 GB: 0.0000

Figure S14. ^{1}H NMR spectrum ($CDCl_3$) of compound **7**.

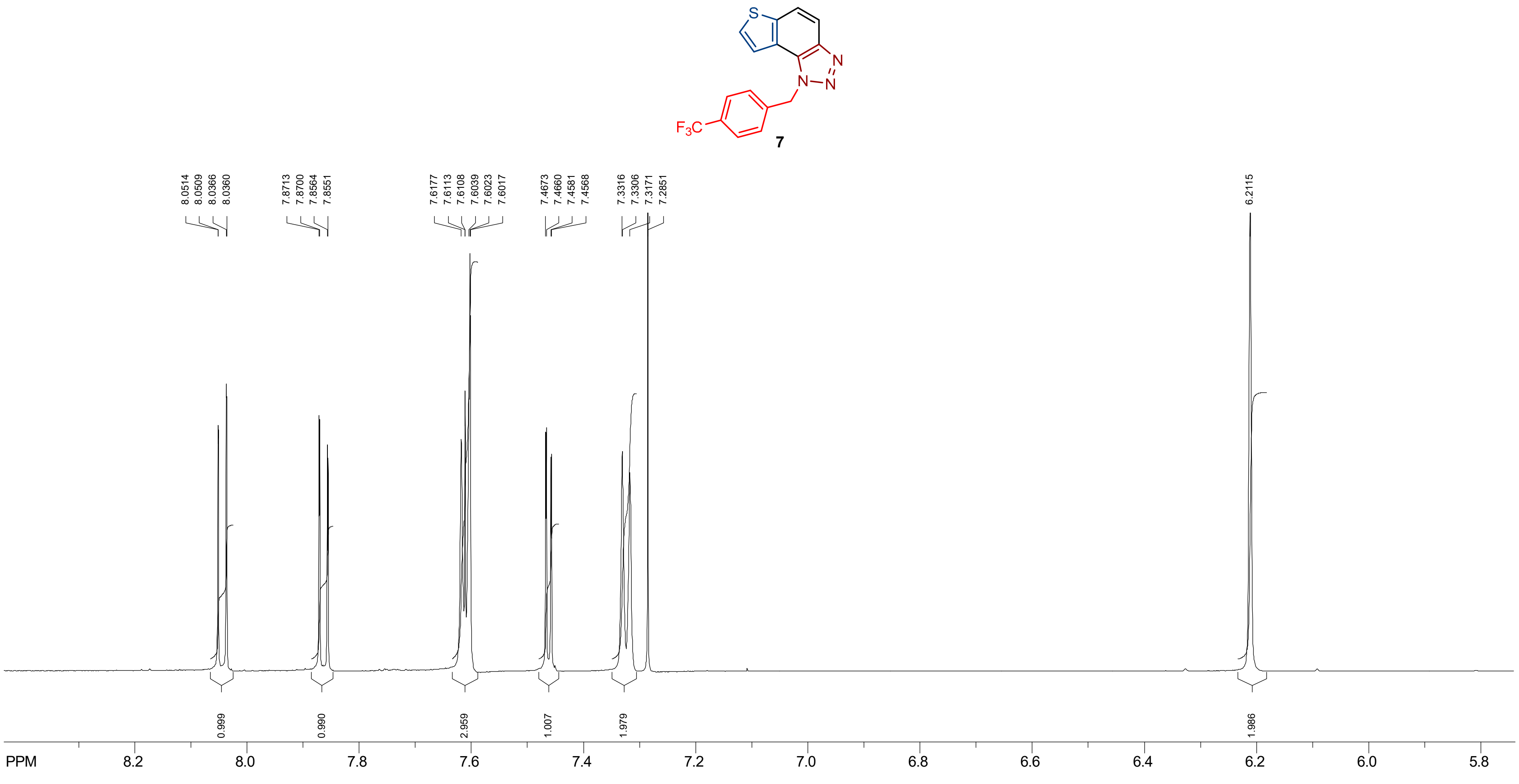

file: C:\Users\Irena\Desktop\IDA SELEC\NMR spektri\SVI KONACNI NMR\Raw data_NMR\icaleta-2023-02-01_33-ZG6 spoj 48P1 1H, 13C\10\fid expt: <zg30>
transmitter freq.: 600.135401 MHz
time domain size: 131072 points
width: 12019.23 Hz = 20.027532 ppm = 0.091699 Hz/pt
number of scans: 64

freq. of 0 ppm: 600.130000 MHz
processed size: 65536 complex points
LB: 0.000 GB: 0.0000

Figure S15. Part of the ^{1}H NMR spectrum ($CDCl_3$) of compound **7**.

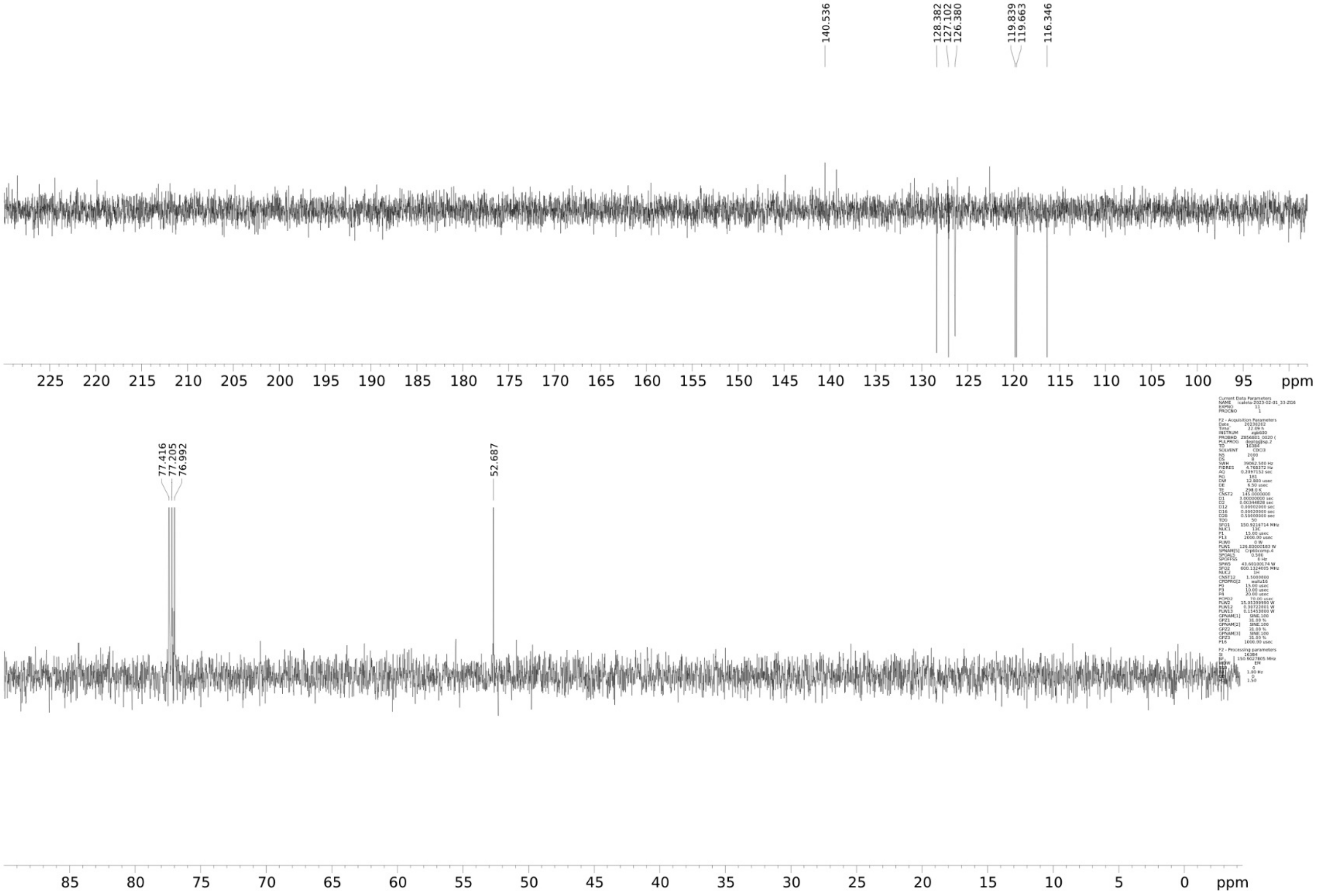

Figure S16. ^{13}C NMR spectrum ($CDCl_3$) of compound **7**.

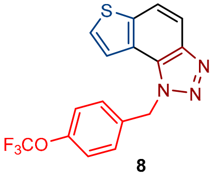

8

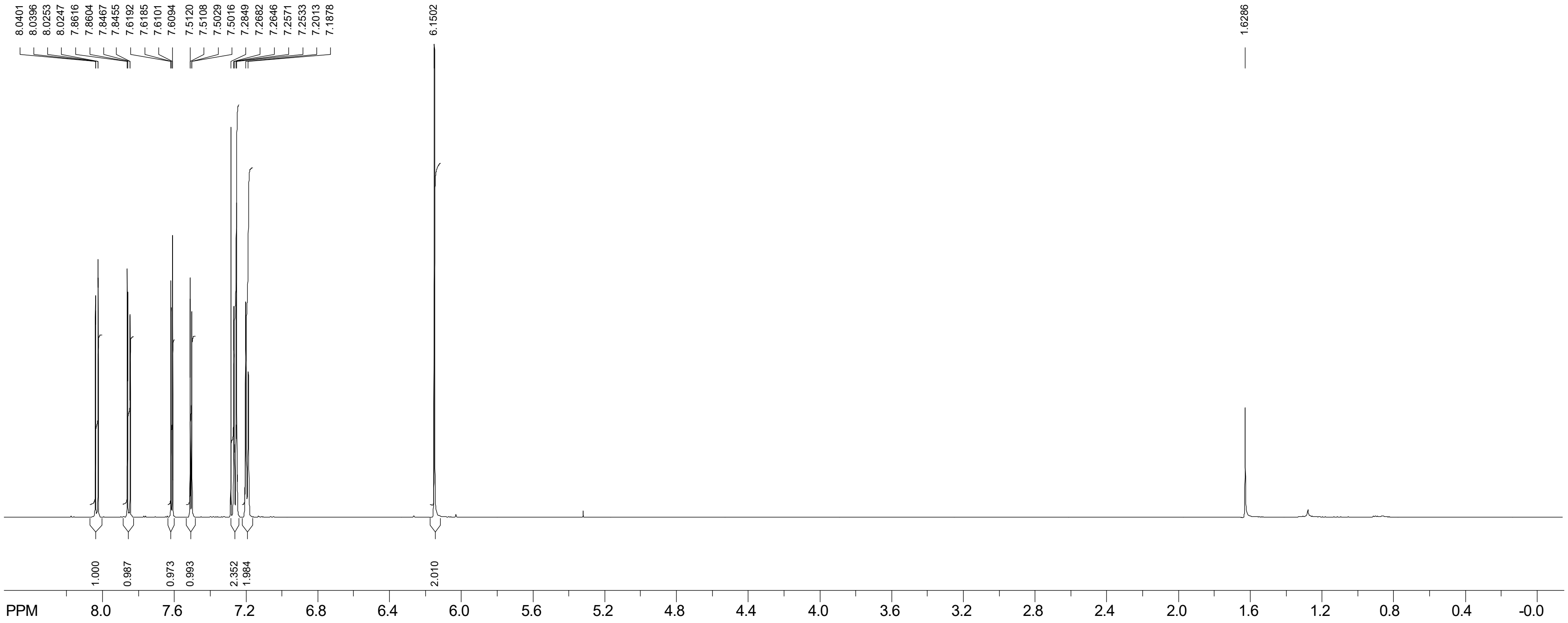

file: C:\Users\Irena\Desktop\IJMS II 2023\IDA SELEC\NMR spektri\SVI KONACNI NMR\NMR KONACNO ZA KORISTITI!!!\Raw Data_NMR\FID2172-032\10\fid expt: <zg30>
transmitter freq.: 600.135401 MHz
time domain size: 131072 points
width: 12019.23 Hz = 20.027532 ppm = 0.091699 Hz/pt
number of scans: 64

freq. of 0 ppm: 600.130000 MHz
processed size: 65536 complex points
LB: 0.000 GB: 0.0000

Figure S17. ^{1}H NMR spectrum ($CDCl_3$) of compound **8**.

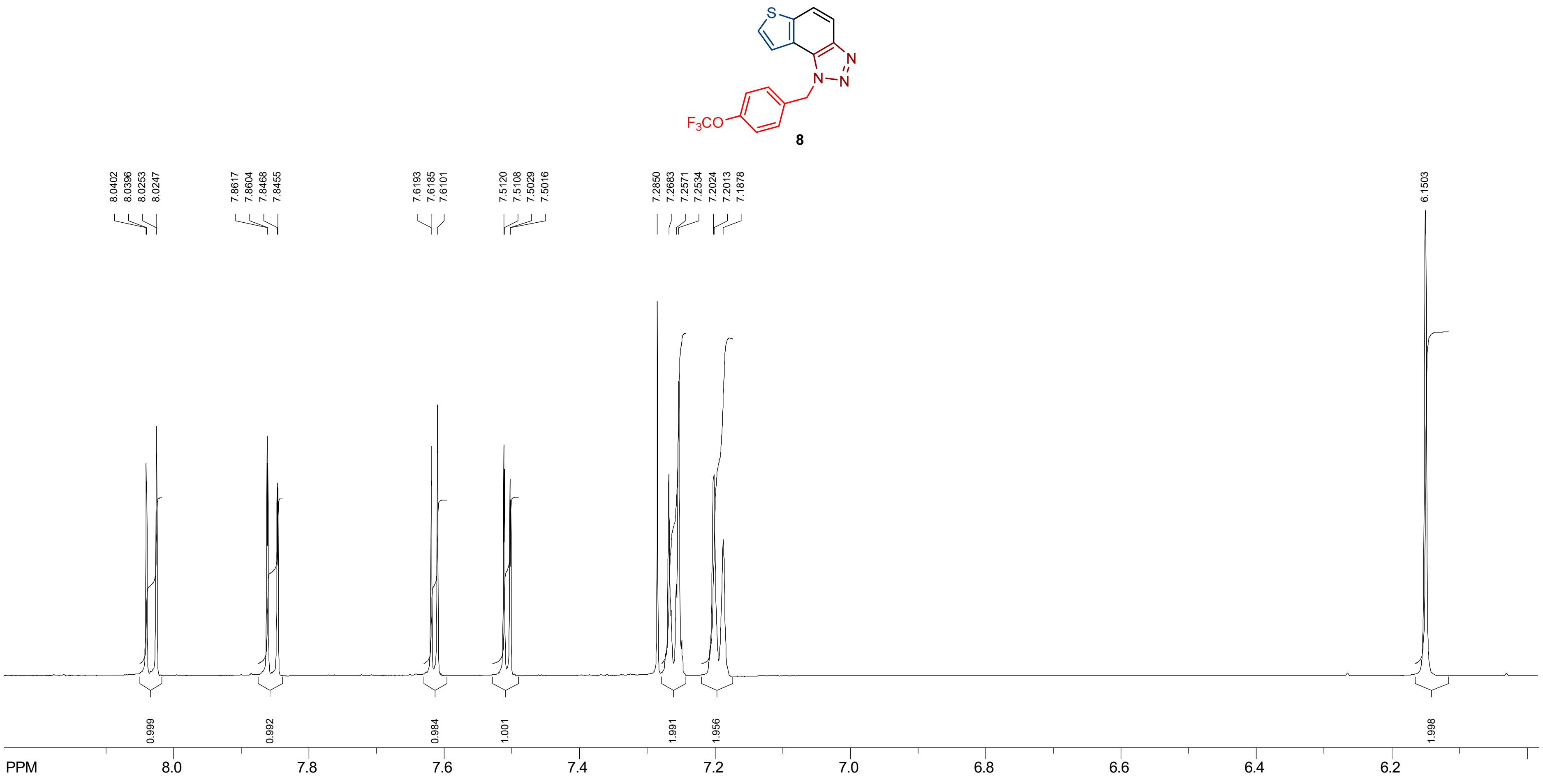

file: C:\Users\Irena\Desktop\IDA SELEC\NMR spektri\SVI KONACNI NMR\Raw data_NMR\icaleta-2023-02-01_35-ZG6 spoj 32P2 1H\10\fid expt: <zg30>
transmitter freq.: 600.135401 MHz
time domain size: 131072 points
width: 12019.23 Hz = 20.027532 ppm = 0.091699 Hz/pt
number of scans: 64

freq. of 0 ppm: 600.130000 MHz
processed size: 65536 complex points
LB: 0.000 GB: 0.0000

Figure S18. Part of the ^{1}H NMR spectrum ($CDCl_3$) of compound **8**.

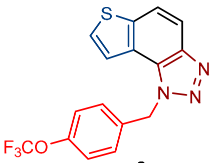

8

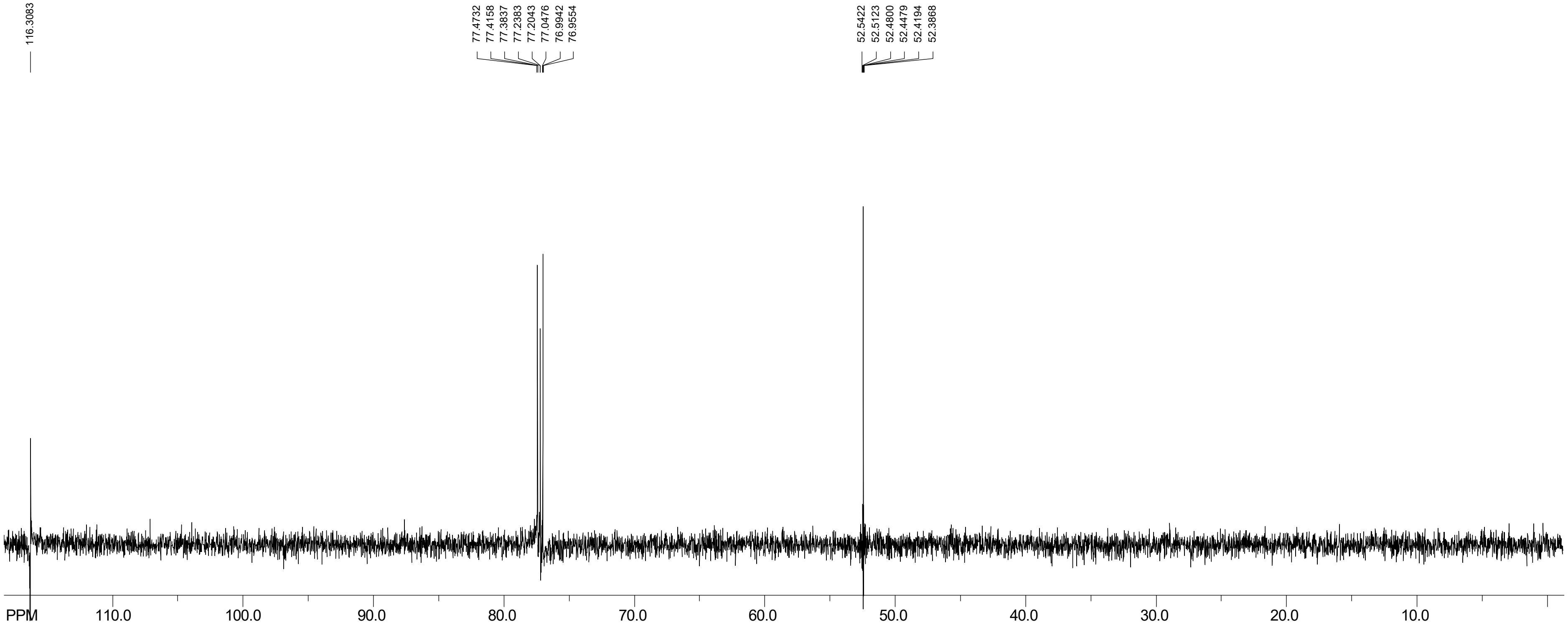

file: C:\Users\Irena\Desktop\IJMS II 2023\IDA SELEC\NMR spektri\SVI KONACNI NMR\NMR KONACNO ZA KORISTITI!!!\Raw Data_NMR\FID2172-032\11\fid expt: <deptqgpsp.2>
transmitter freq.: 150.921671 MHz
time domain size: 16384 points
width: 39062.50 Hz = 258.826315 ppm = 2.384186 Hz/pt
number of scans: 1000

freq. of 0 ppm: 150.902782 MHz
processed size: 16384 complex points
LB: 0.000 GB: 0.0000

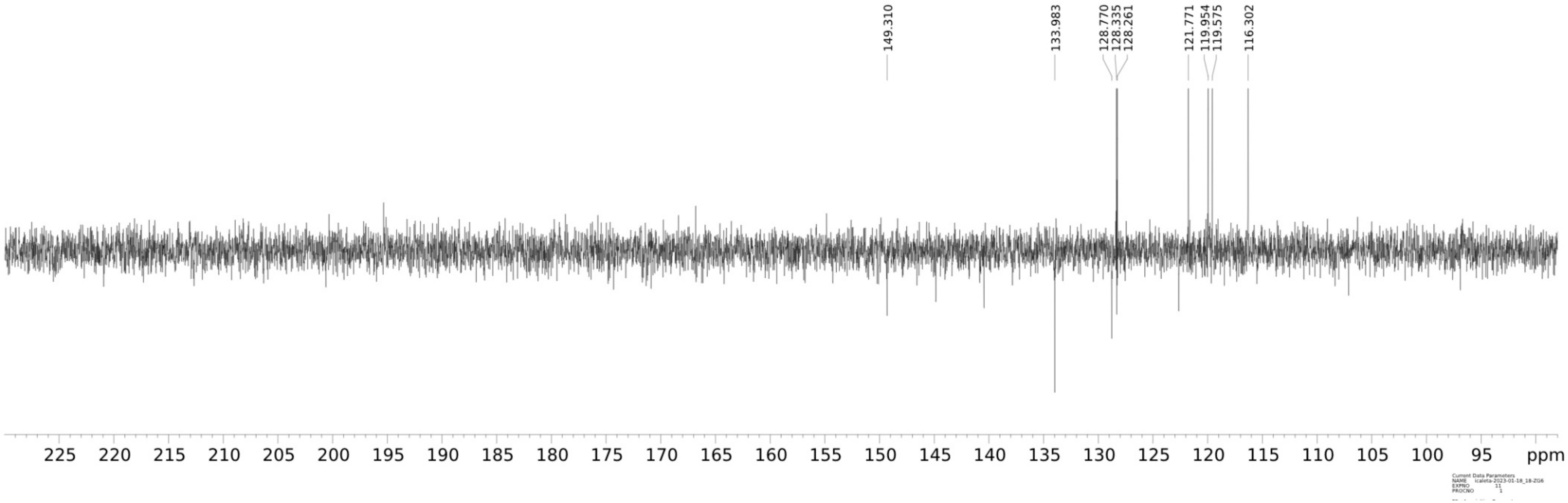

Figure S19. ^{13}C NMR spectrum ($CDCl_3$) of compound **8**.

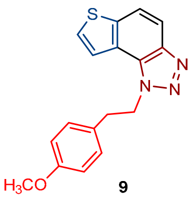

9

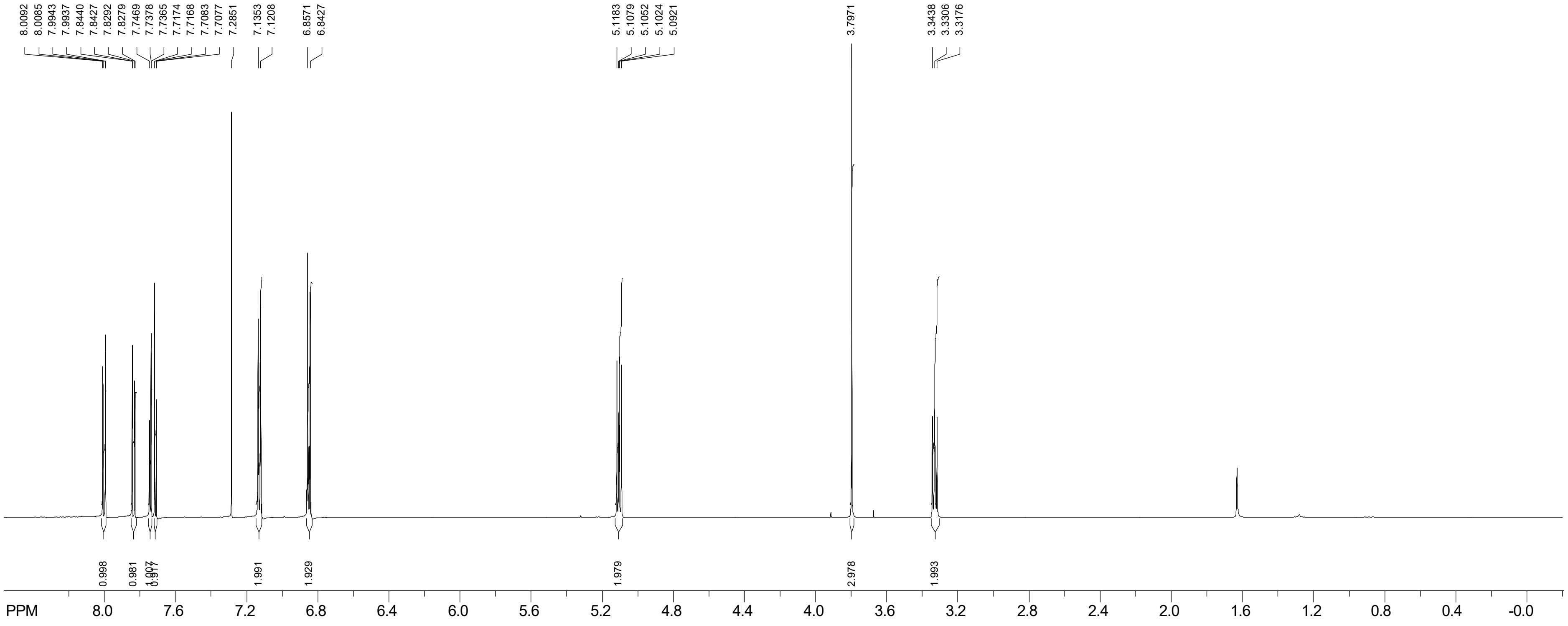

file: C:\Users\Irena\Desktop\IJMS II 2023\IDA SELEC\NMR spektri\SVI KONACNI NMR\NMR KONACNO ZA KORISTITI!!!\Raw Data_NMR\FID2172-034\10\fid expt: <zg30>
transmitter freq.: 600.135401 MHz
time domain size: 131072 points
width: 12019.23 Hz = 20.027532 ppm = 0.091699 Hz/pt
number of scans: 64

freq. of 0 ppm: 600.130000 MHz
processed size: 65536 complex points
LB: 0.000 GB: 0.0000

Figure S20. ^{1}H NMR spectrum ($CDCl_3$) of compound **9**.

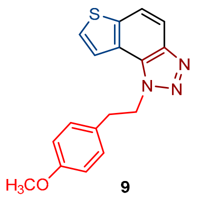

9

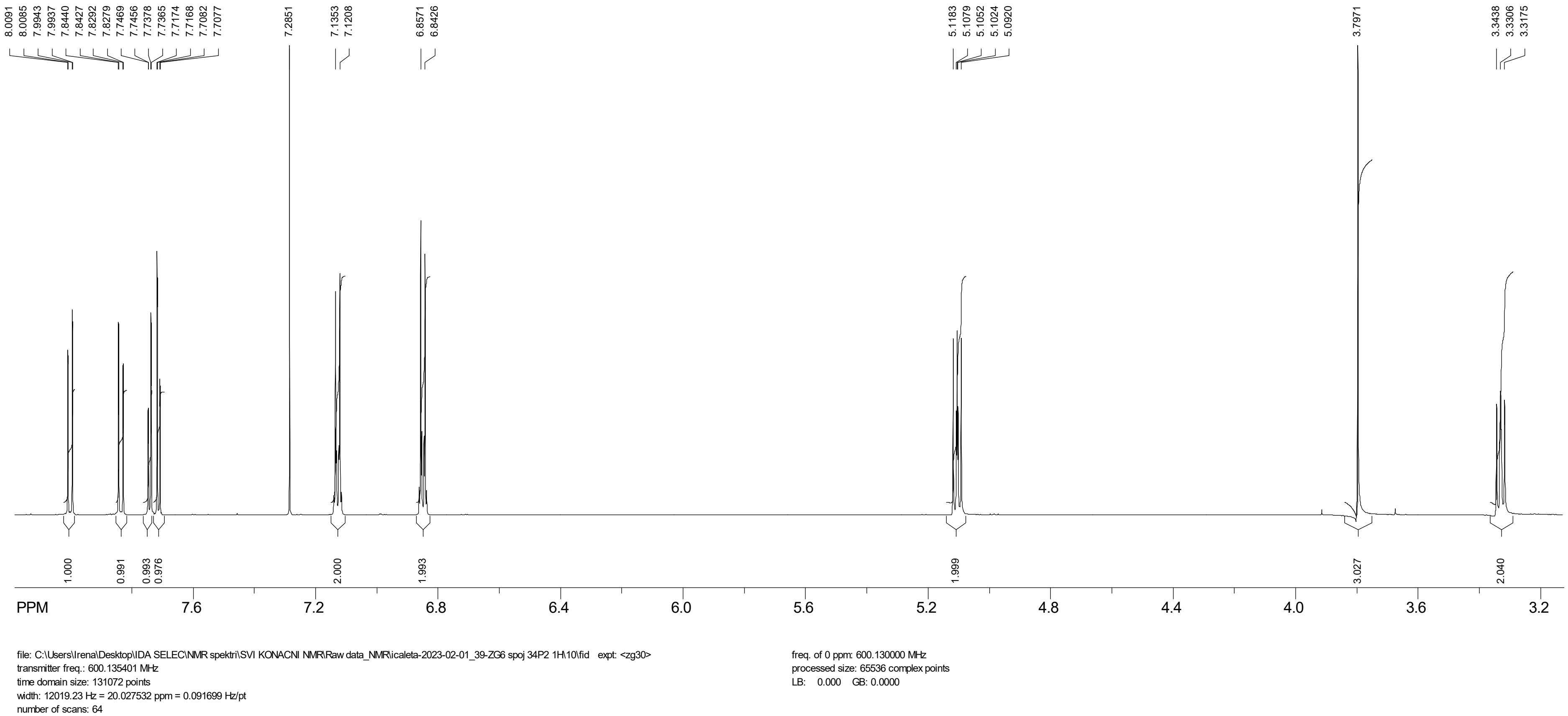

Figure S21. Part of the ^{1}H NMR spectrum ($CDCl_3$) of compound **9**.

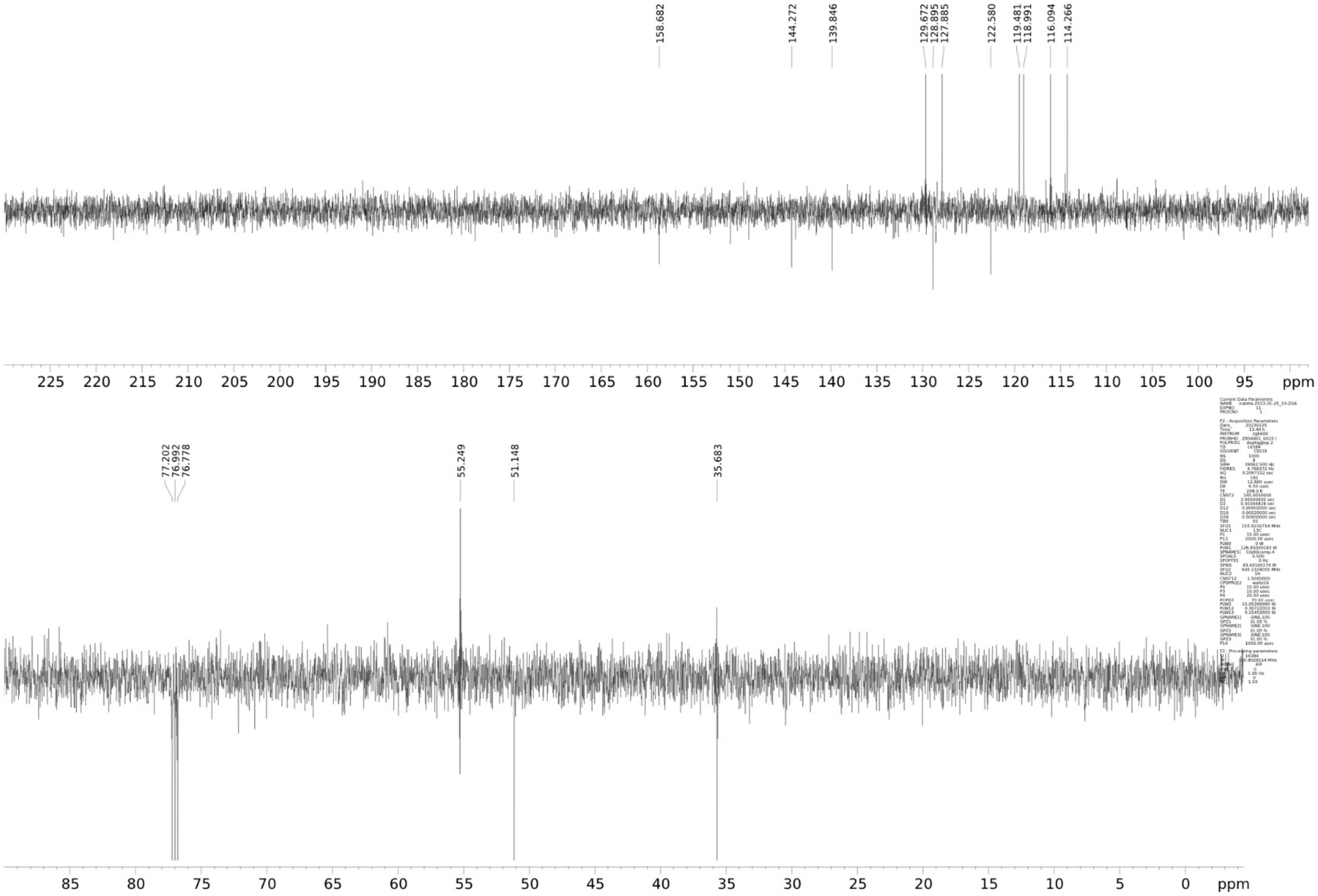

Figure S22. ^{13}C NMR spectrum ($CDCl_3$) of compound **9**.

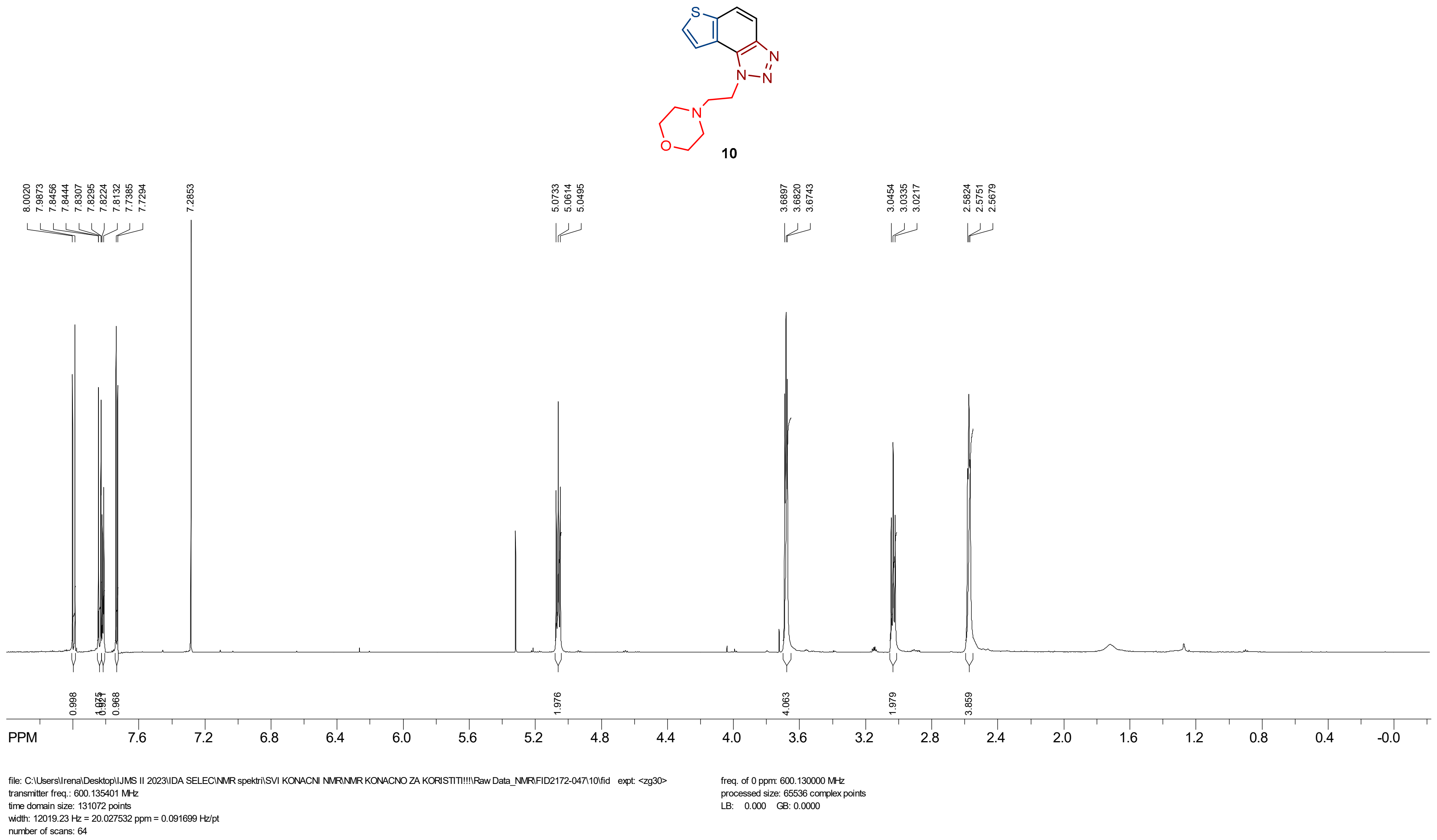

Figure S23. ^{1}H NMR spectrum ($CDCl_3$) of compound **10**.

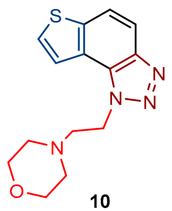

10

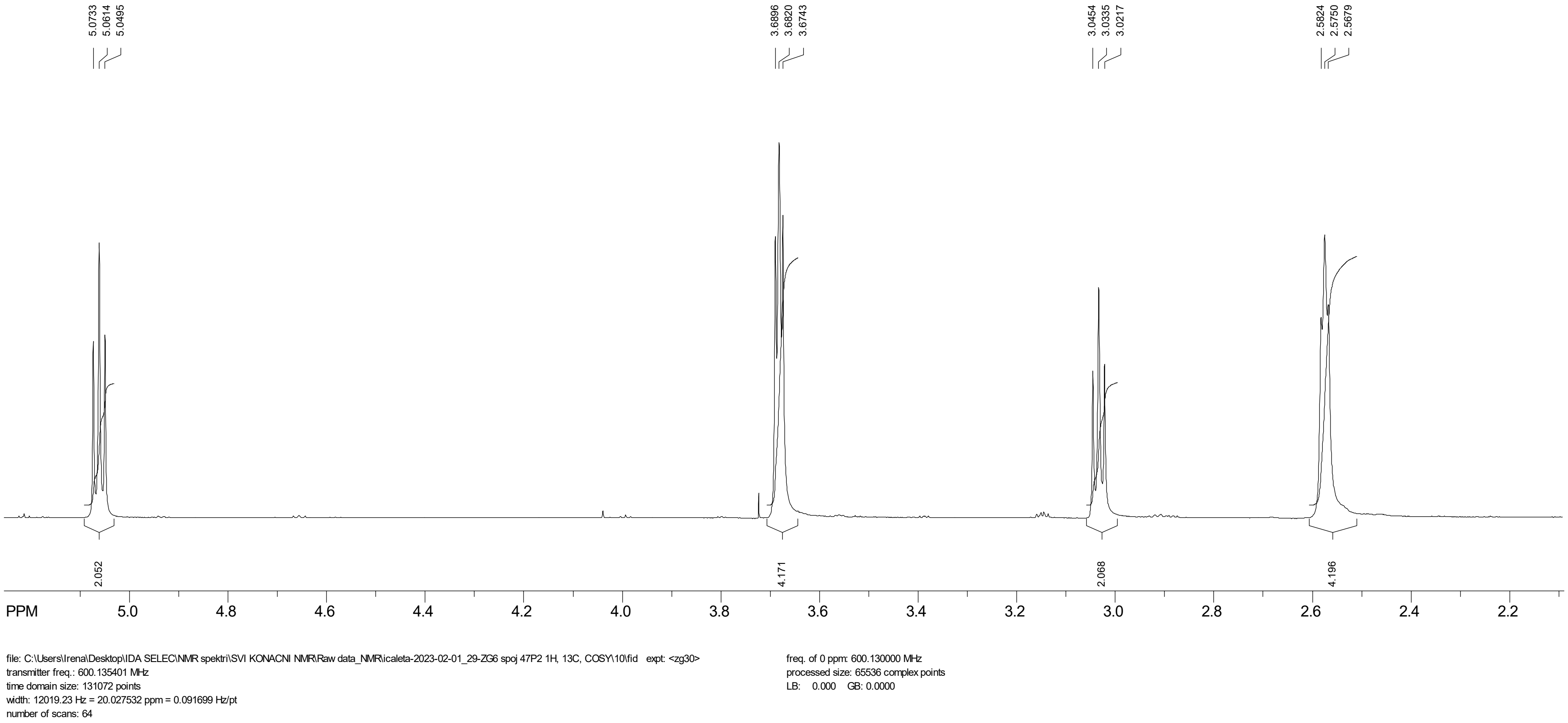

Figure S24. Aliphatic part of the ^{1}H NMR spectrum ($CDCl_3$) of compound **10**.

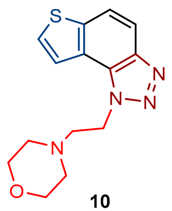

10

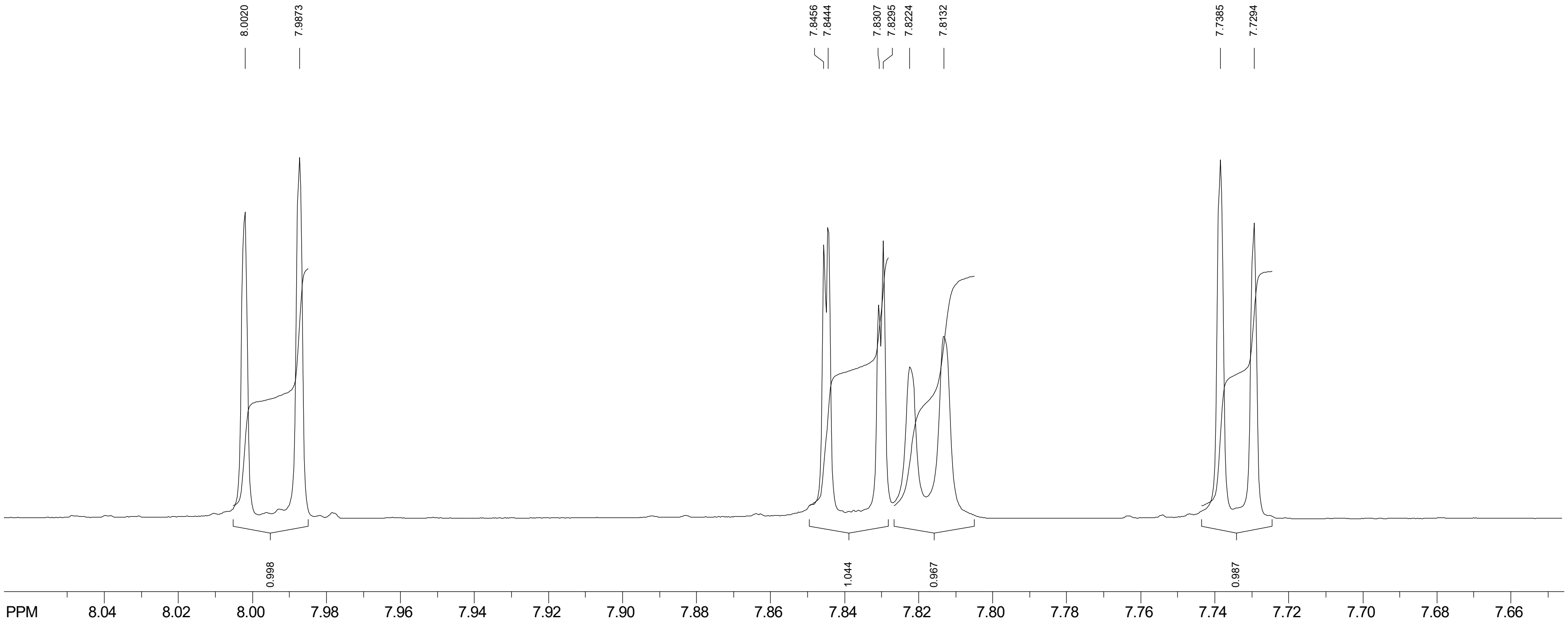

file: C:\Users\Irena\Desktop\IDA SELEC\NMR spektri\SVI KONACNI NMR\Raw data_NMR\icaleta-2023-02-01_29-ZG6 spoj 47P2 1H, 13C, COSY\10\fid expt: <zg30>
transmitter freq.: 600.135401 MHz
time domain size: 131072 points
width: 12019.23 Hz = 20.027532 ppm = 0.091699 Hz/pt
number of scans: 64

freq. of 0 ppm: 600.130000 MHz
processed size: 65536 complex points
LB: 0.000 GB: 0.0000

Figure S25. Aromatic part of the ^{1}H NMR spectrum ($CDCl_3$) of compound **10**.

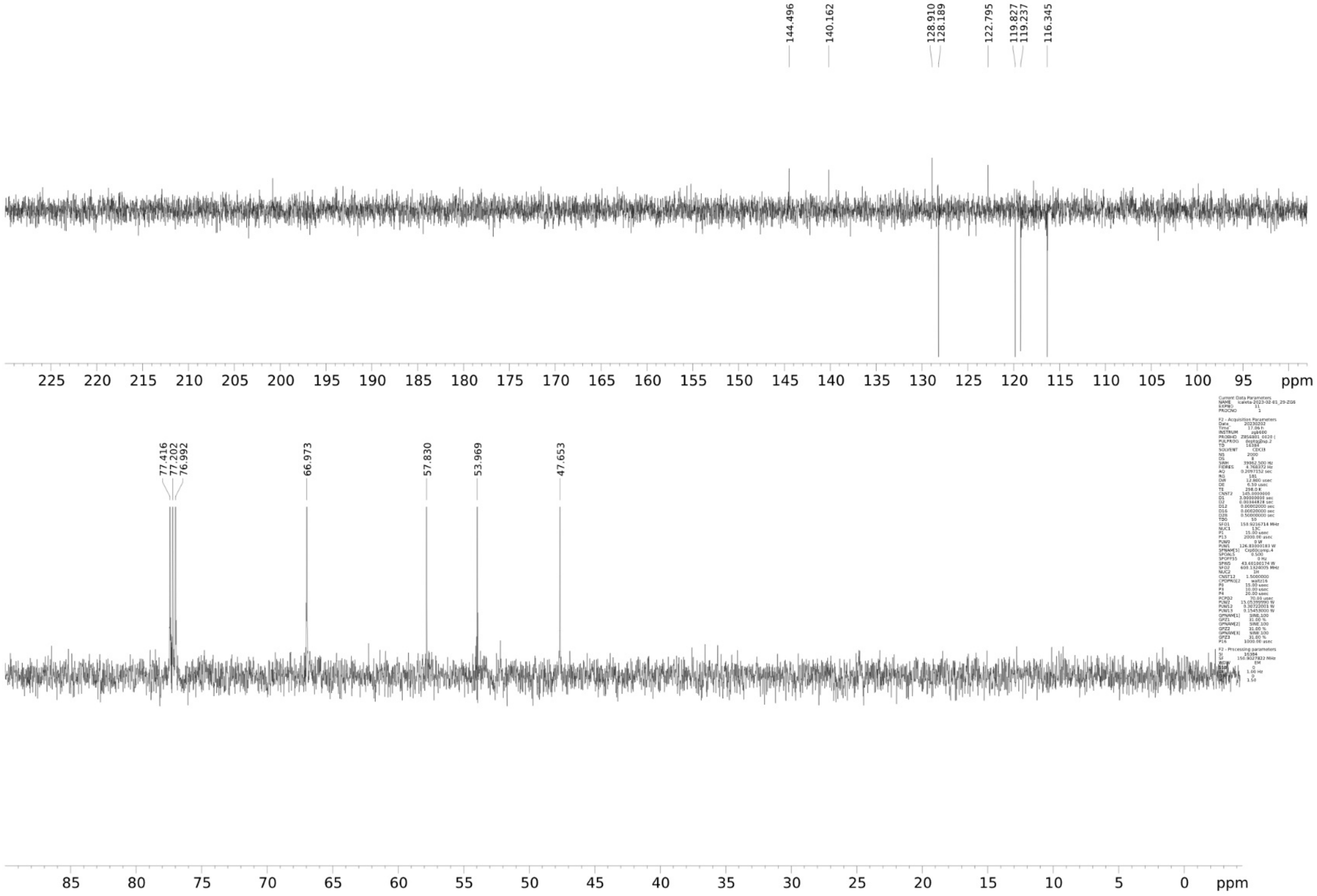

Figure S26. ^{13}C NMR spectrum ($CDCl_3$) of compound **10**.

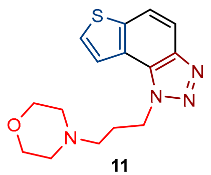

11

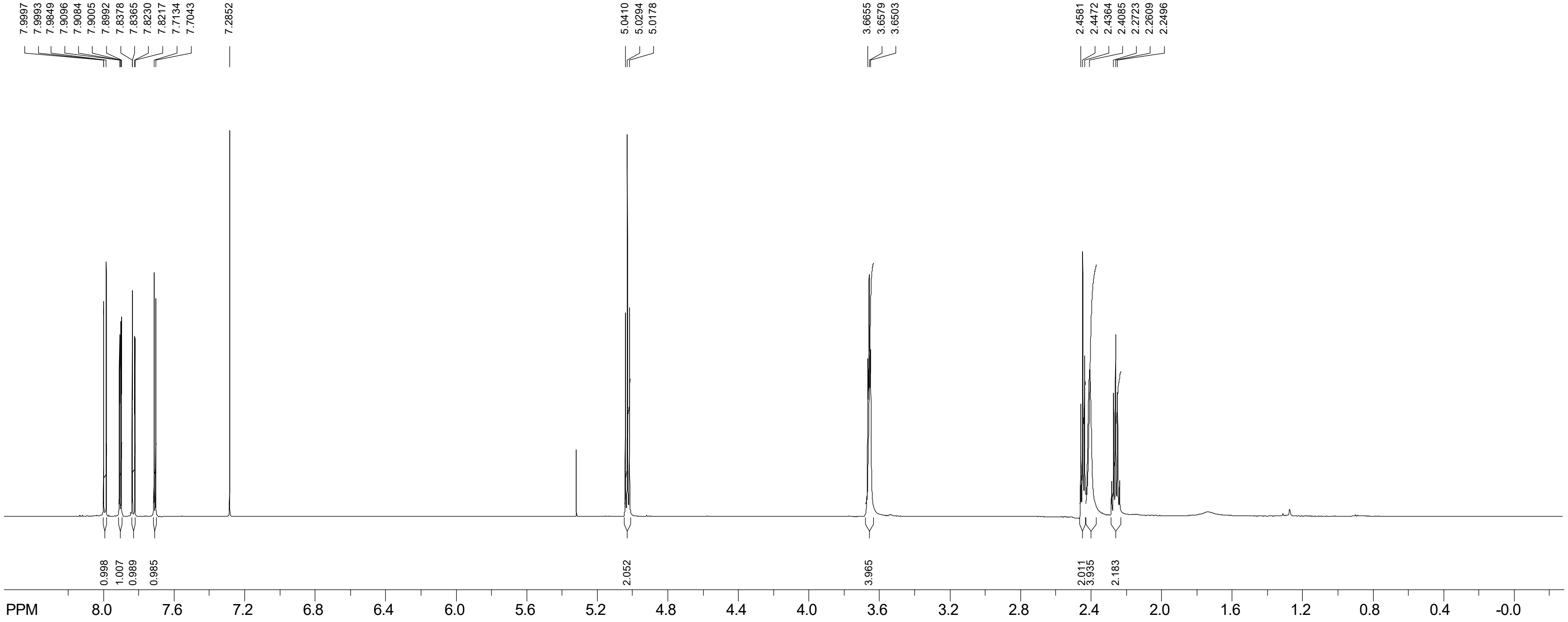

file: C:\Users\Irena\Desktop\IJMS II 2023\IDA SELEC\NMR spektri\SVI KONACNI NMR\NMR KONACNO ZA KORISTITI!!!\Raw Data_NMR\FID2172-040\10\fid expt: <zg30>
transmitter freq.: 600.135401 MHz
time domain size: 131072 points
width: 12019.23 Hz = 20.027532 ppm = 0.091699 Hz/pt
number of scans: 64

freq. of 0 ppm: 600.130000 MHz
processed size: 65536 complex points
LB: 0.000 GB: 0.0000

Figure S27. ^{1}H NMR spectrum ($CDCl_3$) of compound **11**.

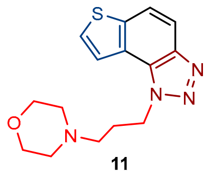

11

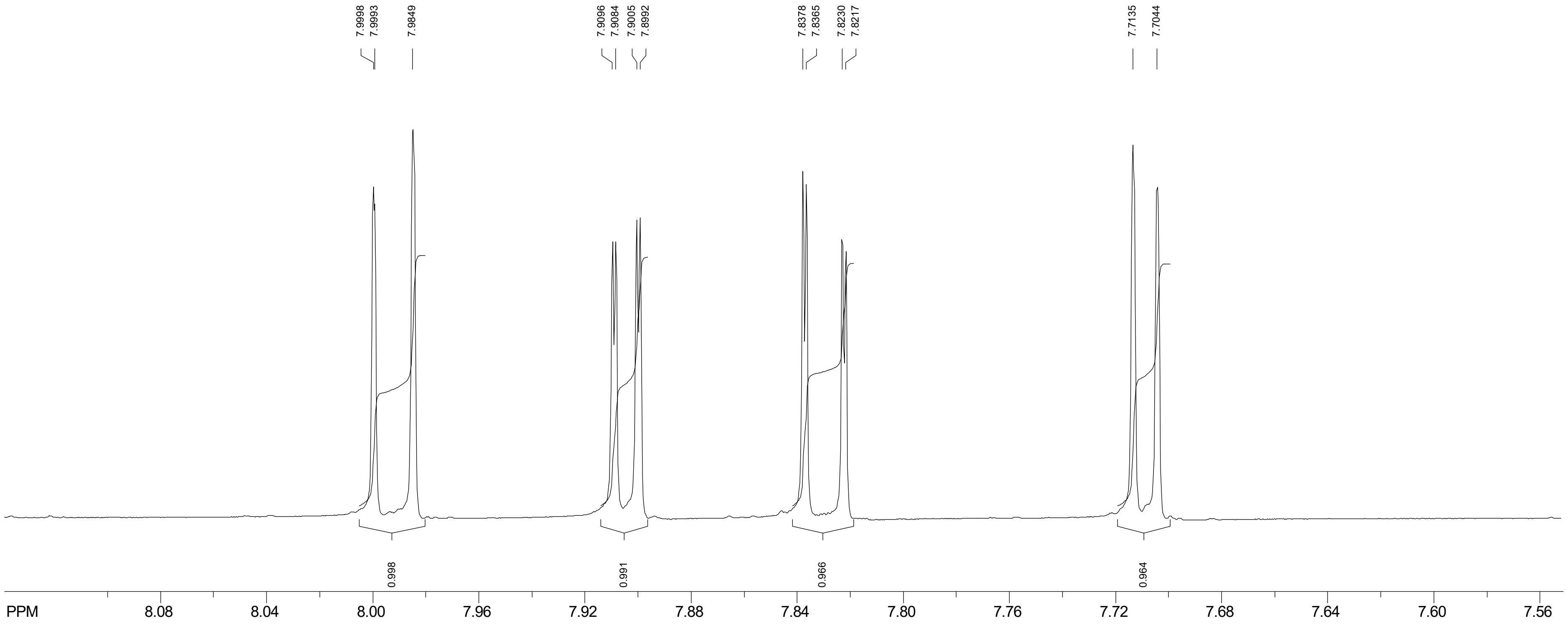

file: C:\Users\Irena\Desktop\IDA SELEC\NMR spektri\SVI KONACNI NMR\Raw data_NMR\icaleta-2023-02-01_31-ZG6 spoj 40P2 1H\10\fid expt: <zg30>
transmitter freq.: 600.135401 MHz
time domain size: 131072 points
width: 12019.23 Hz = 20.027532 ppm = 0.091699 Hz/pt
number of scans: 64

freq. of 0 ppm: 600.130000 MHz
processed size: 65536 complex points
LB: 0.000 GB: 0.0000

Figure S28. Aromatic part of the ^{1}H NMR spectrum ($CDCl_3$) of compound **11**.

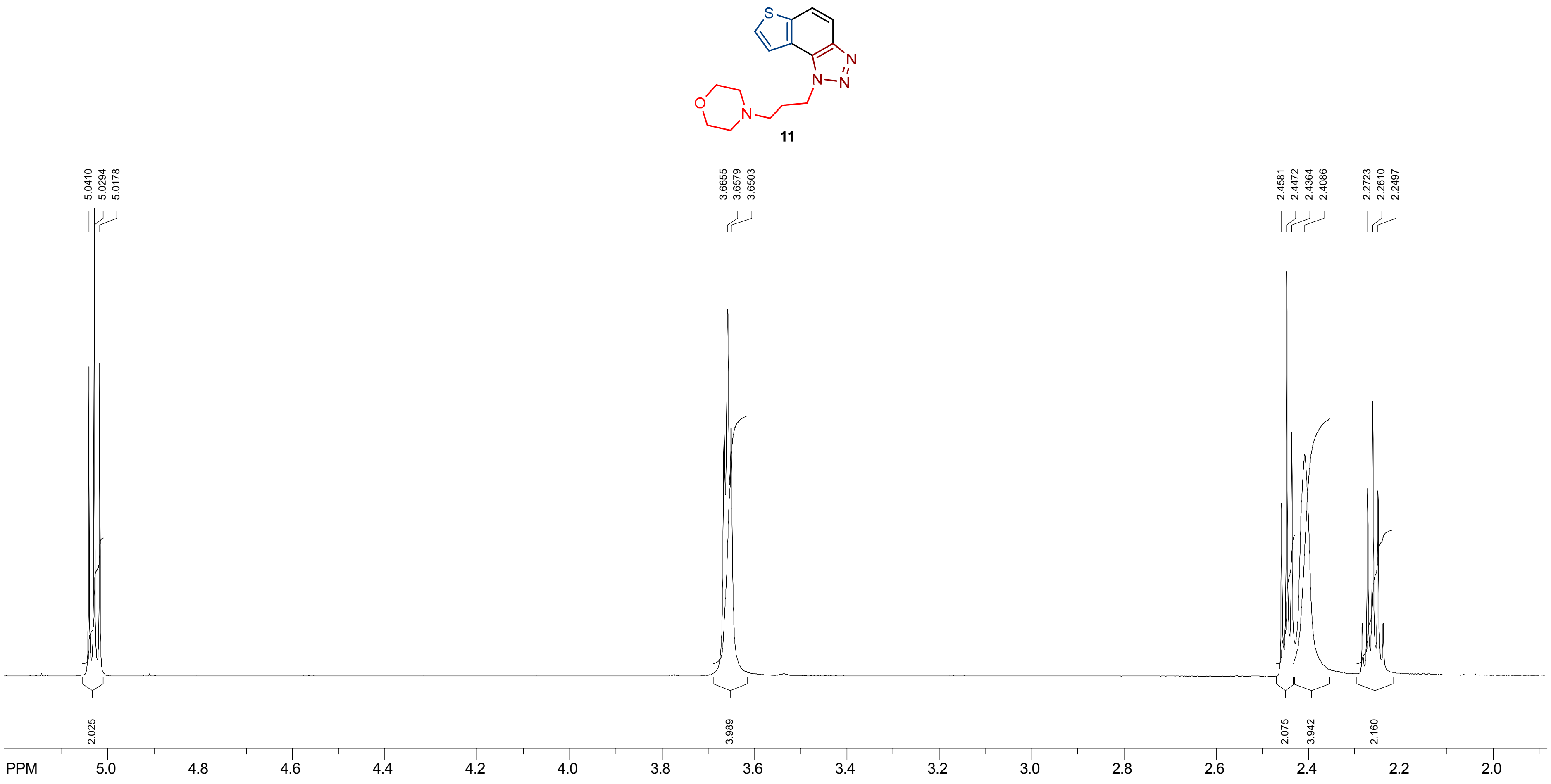

file: C:\Users\Irena\Desktop\IDA SELEC\NMR spektri\SVI KONACNI NMR\Raw data_NMR\icaleta-2023-02-01_31-ZG6 spoj 40P2 1H\10\fid expt: <zg30>
transmitter freq.: 600.135401 MHz
time domain size: 131072 points
width: 12019.23 Hz = 20.027532 ppm = 0.091699 Hz/pt
number of scans: 64

freq. of 0 ppm: 600.130000 MHz
processed size: 65536 complex points
LB: 0.000 GB: 0.0000

Figure S29. Aliphatic part of the ^{1}H NMR spectrum ($CDCl_3$) of compound **11**.

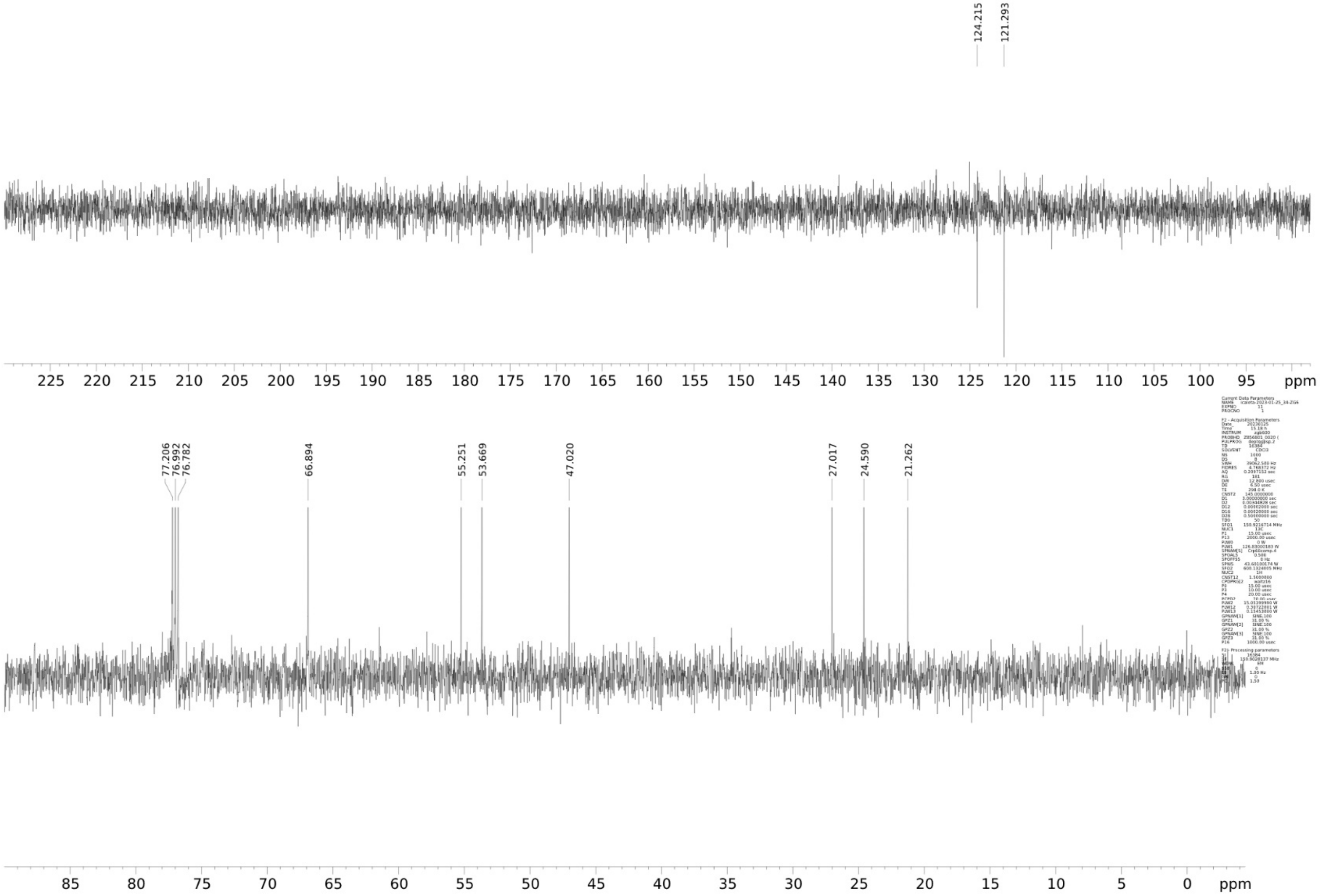

Figure S30. ^{13}C NMR spectrum ($CDCl_3$) of compound **11**.

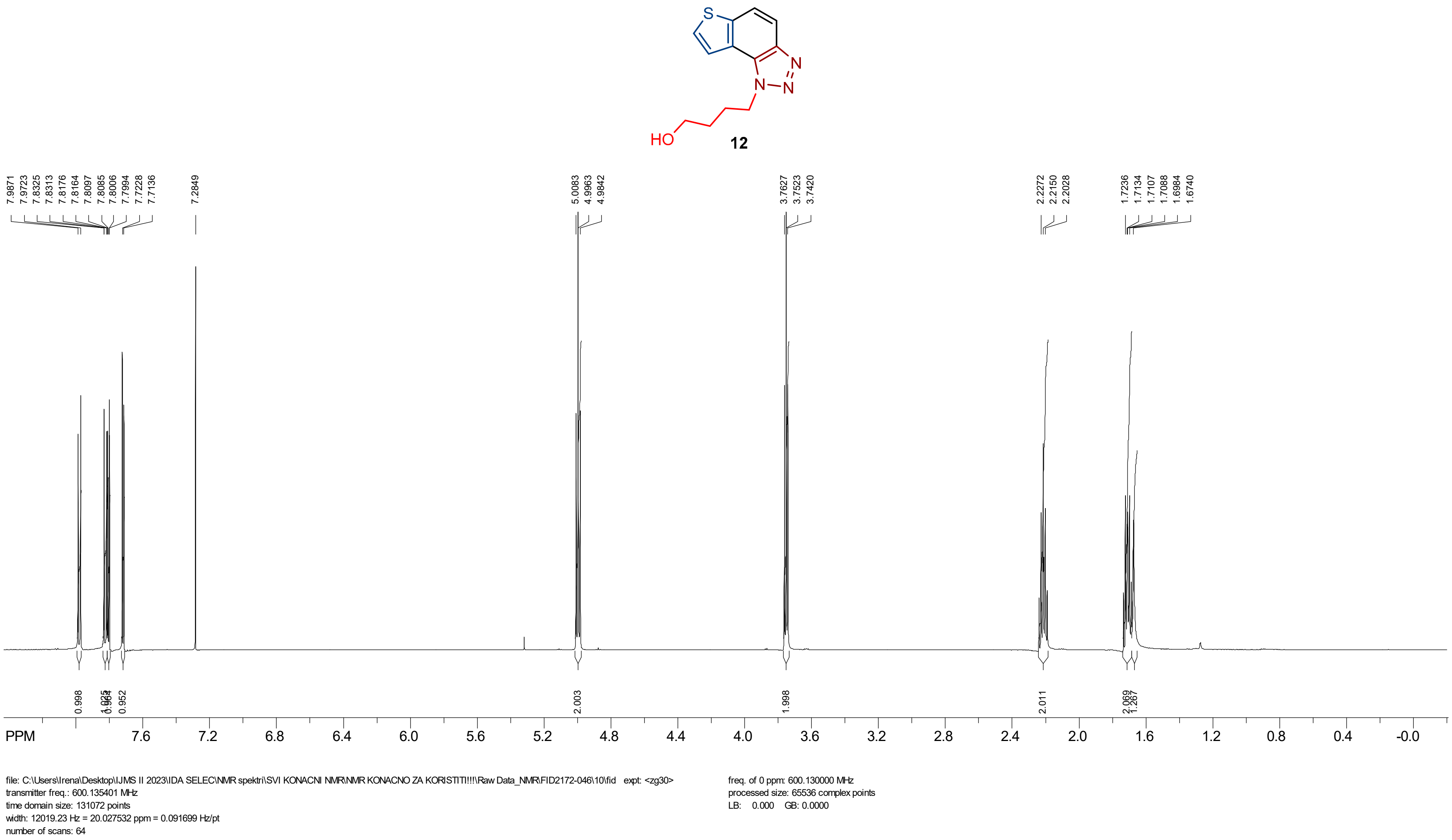

Figure S31. ^{1}H NMR spectrum ($CDCl_3$) of compound **12**.

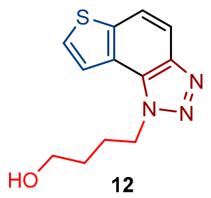

12

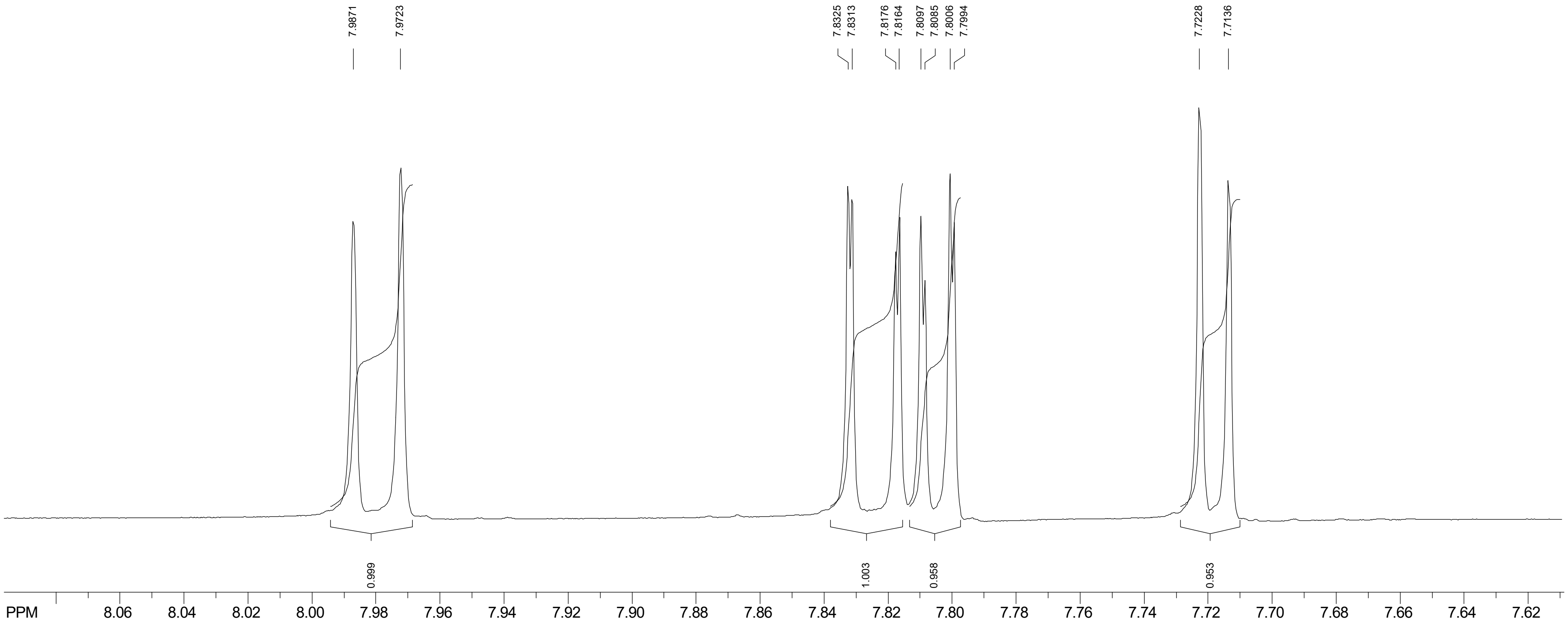

file: C:\Users\Irena\Desktop\IDA SELEC\NMR spektri\SVI KONACNI NMR\Raw data_NMR\icaleta-2023-02-01_41-ZG6 spoj 46P2 1H, 13C, COSY\10\fid expt: <zg30>
transmitter freq.: 600.135401 MHz
time domain size: 131072 points
width: 12019.23 Hz = 20.027532 ppm = 0.091699 Hz/pt
number of scans: 64

freq. of 0 ppm: 600.130000 MHz
processed size: 65536 complex points
LB: 0.000 GB: 0.0000

Figure S32. Aromatic part of the ^{1}H NMR spectrum ($CDCl_3$) of compound **12**.

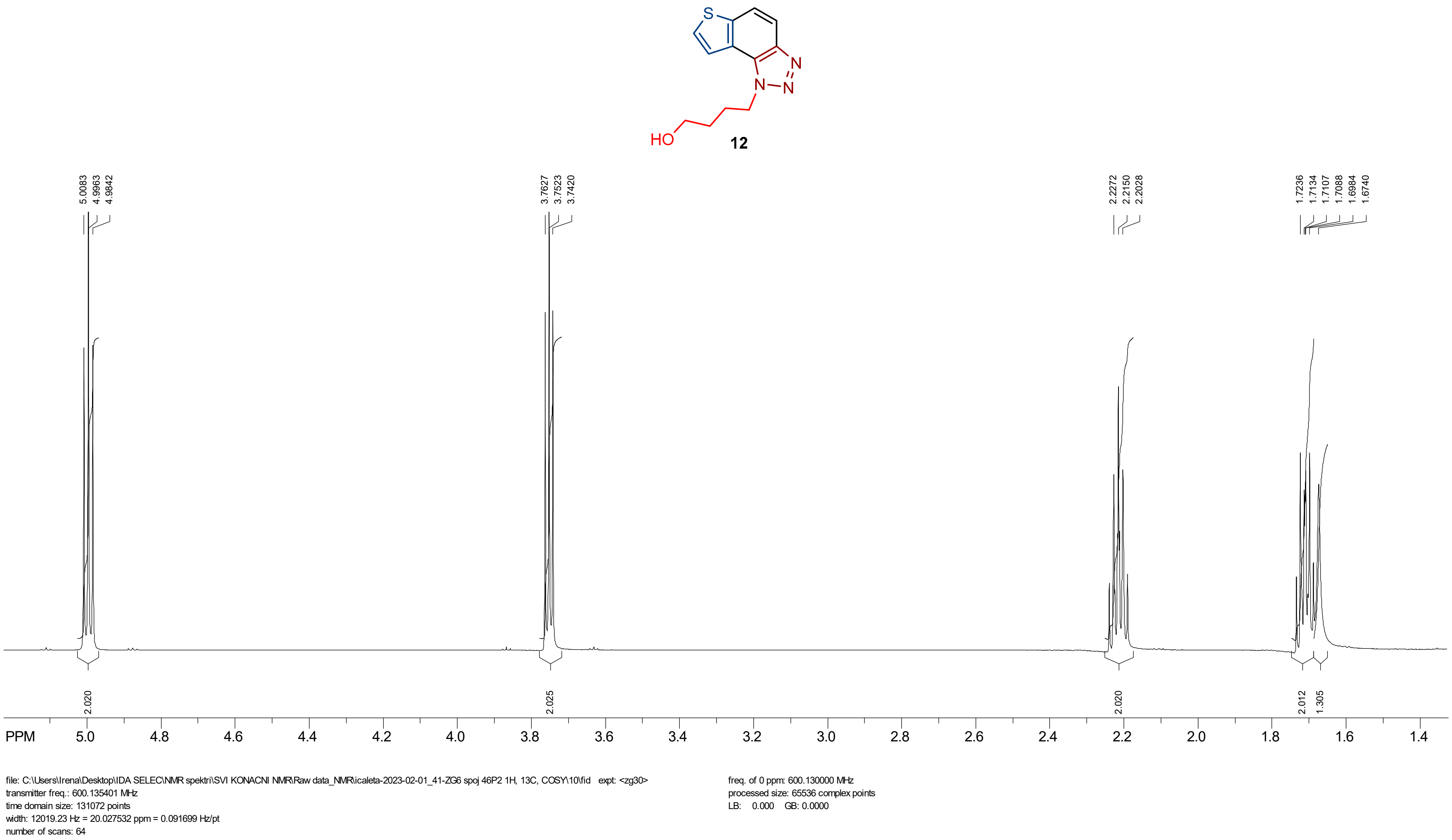

Figure S33. Aliphatic part of the ^{1}H NMR spectrum ($CDCl_3$) of compound **12**.

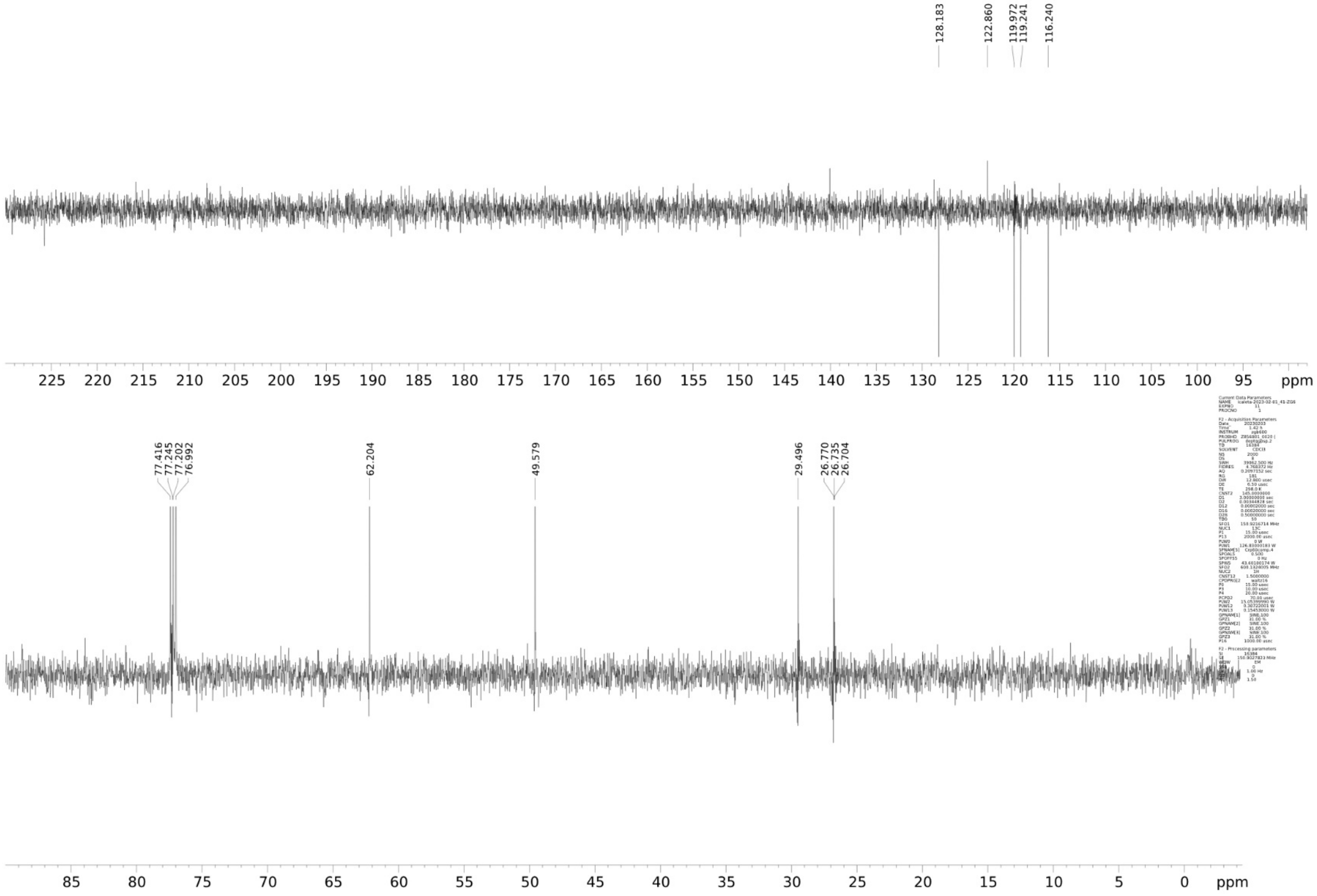

Figure S34. ^{13}C NMR spectrum ($CDCl_3$) of compound **12**.

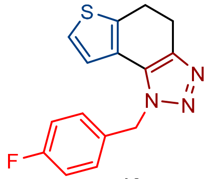

13

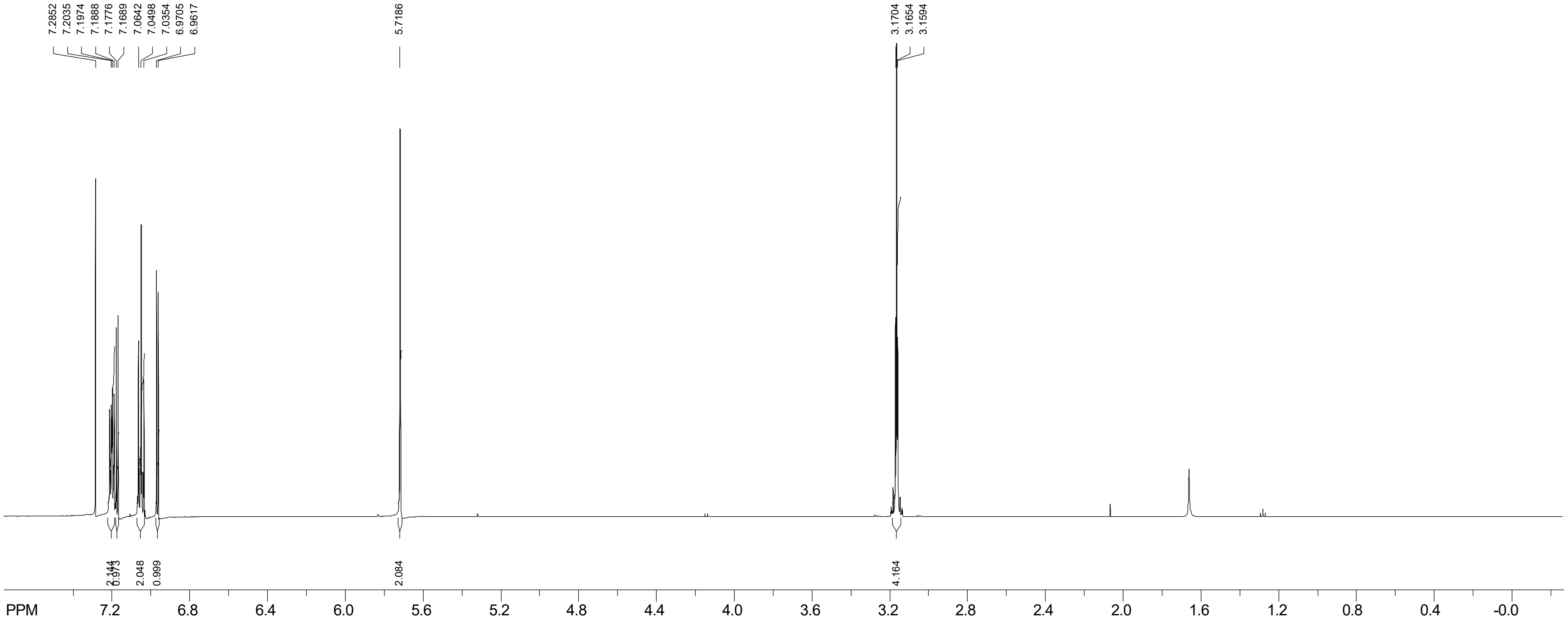

file: C:\Users\Irena\Desktop\IJMS II 2023\IDA SELEC\NMR spektri\SVI KONACNI NMR\NMR KONACNO ZA KORISTITI!!!\Raw Data_NMR\FID2172-028\10\fid expt: <zg30>
transmitter freq.: 600.135401 MHz
time domain size: 131072 points
width: 12019.23 Hz = 20.027532 ppm = 0.091699 Hz/pt
number of scans: 64

freq. of 0 ppm: 600.130000 MHz
processed size: 65536 complex points
LB: 0.000 GB: 0.0000

Figure S35. ^{1}H NMR spectrum ($CDCl_3$) of compound **13**.

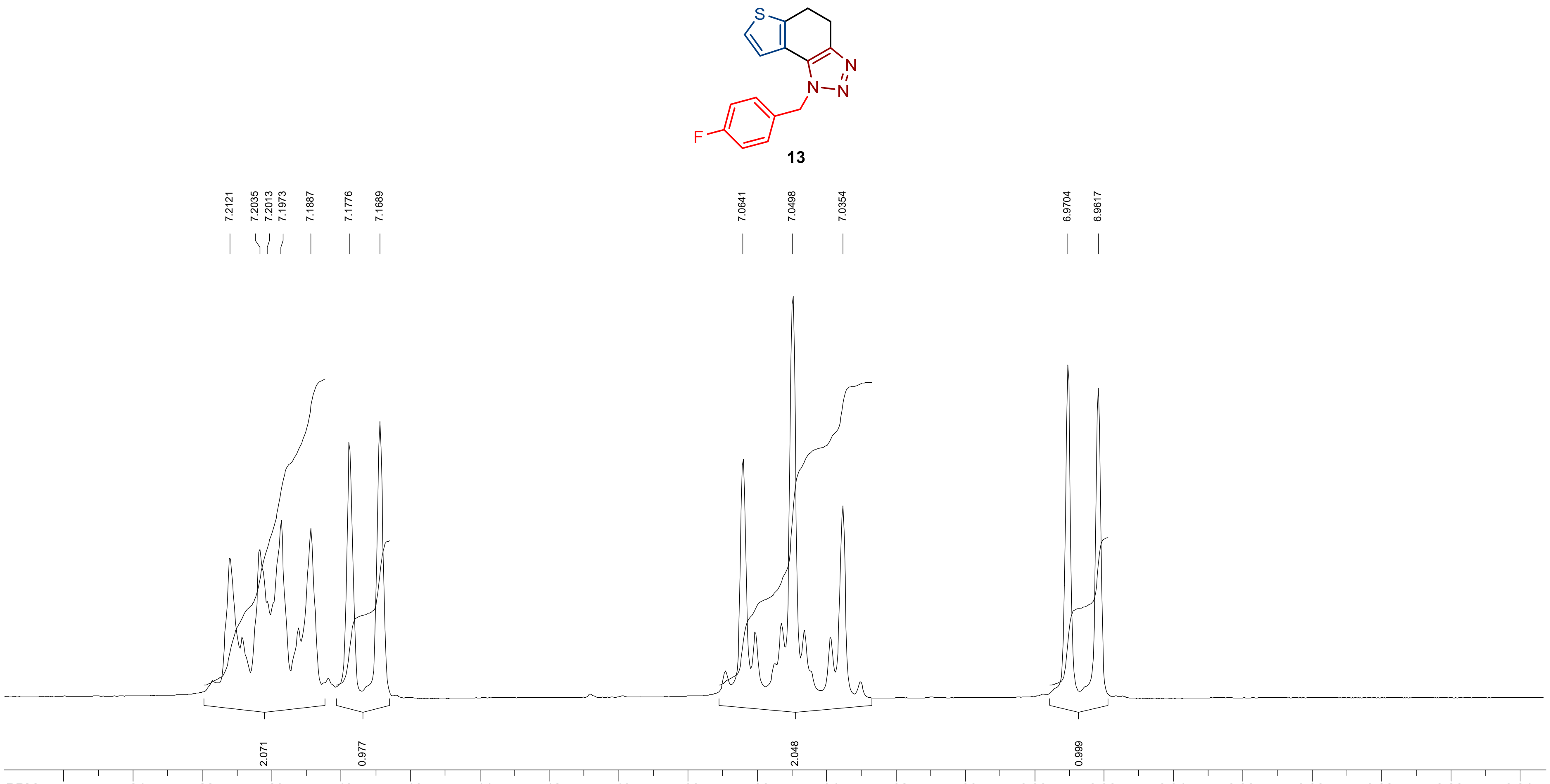

file: C:\Users\Irena\Desktop\IDA SELEC\NMR spektri\SVI KONACNI NMR\Raw data_NMR\icaleta-2023-02-01_36-ZG6 spoj 28P2 1H\10\fid expt: <zg30>
transmitter freq.: 600.135401 MHz
time domain size: 131072 points
width: 12019.23 Hz = 20.027532 ppm = 0.091699 Hz/pt
number of scans: 64

freq. of 0 ppm: 600.130000 MHz
processed size: 65536 complex points
LB: 0.000 GB: 0.0000

Figure S36. Aromatic part of the ^{1}H NMR spectrum ($CDCl_3$) of compound **13**.

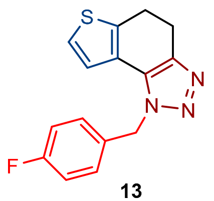

13

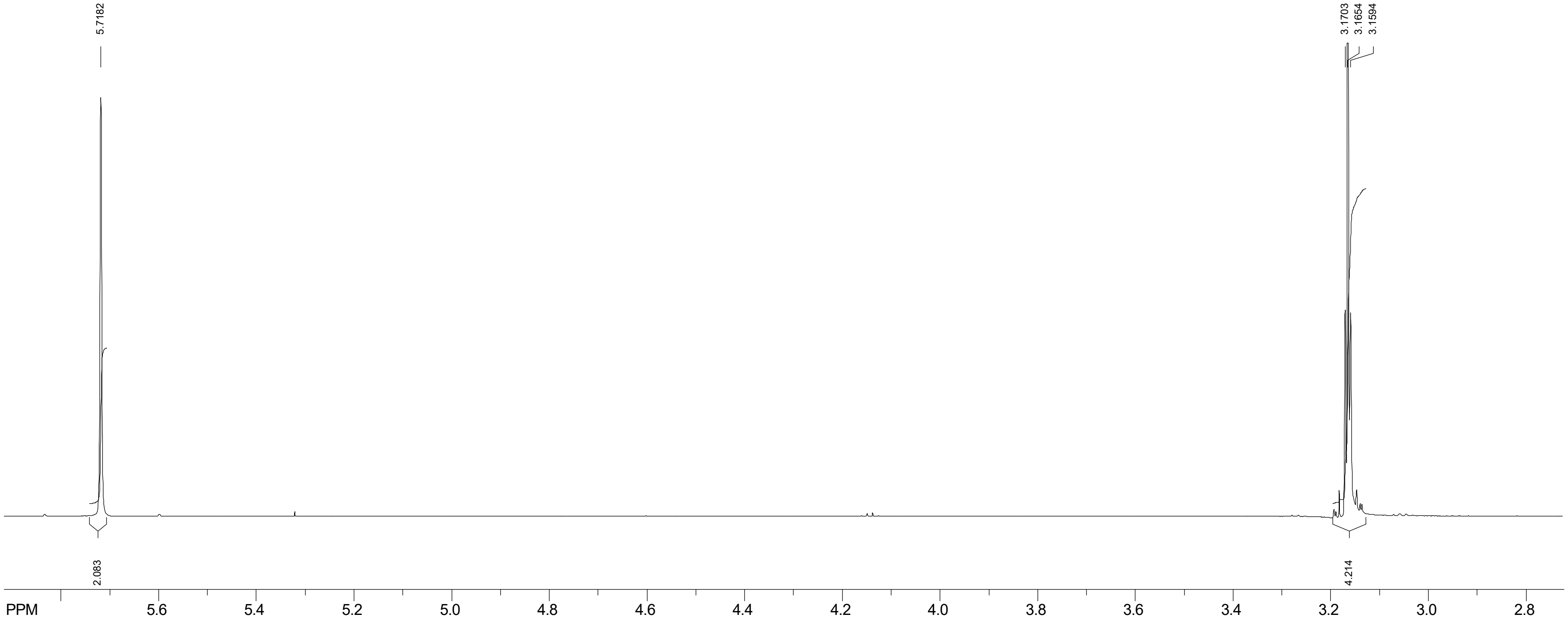

file: C:\Users\Irena\Desktop\IDA SELEC\NMR spektri\SVI KONACNI NMR\Raw data_NMR\icaleta-2023-02-01_36-ZG6 spoj 28P2 1H\10\fid expt: <zg30>
transmitter freq.: 600.135401 MHz
time domain size: 131072 points
width: 12019.23 Hz = 20.027532 ppm = 0.091699 Hz/pt
number of scans: 64

freq. of 0 ppm: 600.130000 MHz
processed size: 65536 complex points
LB: 0.000 GB: 0.0000

Figure S37. Aliphatic part of the ^{1}H NMR spectrum ($CDCl_3$) of compound **13**.

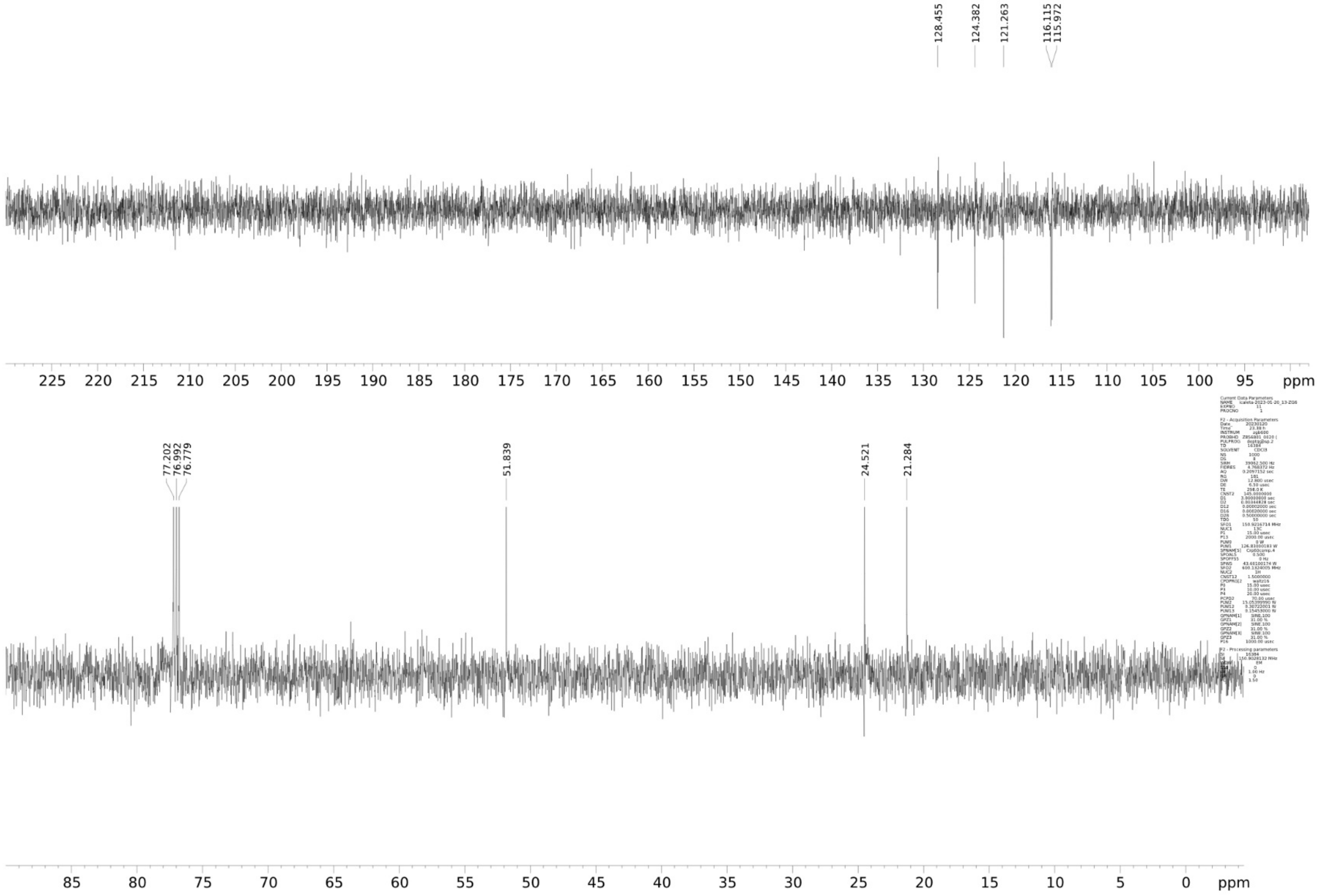

Figure S38. ^{13}C NMR spectrum ($CDCl_3$) of compound **13**.

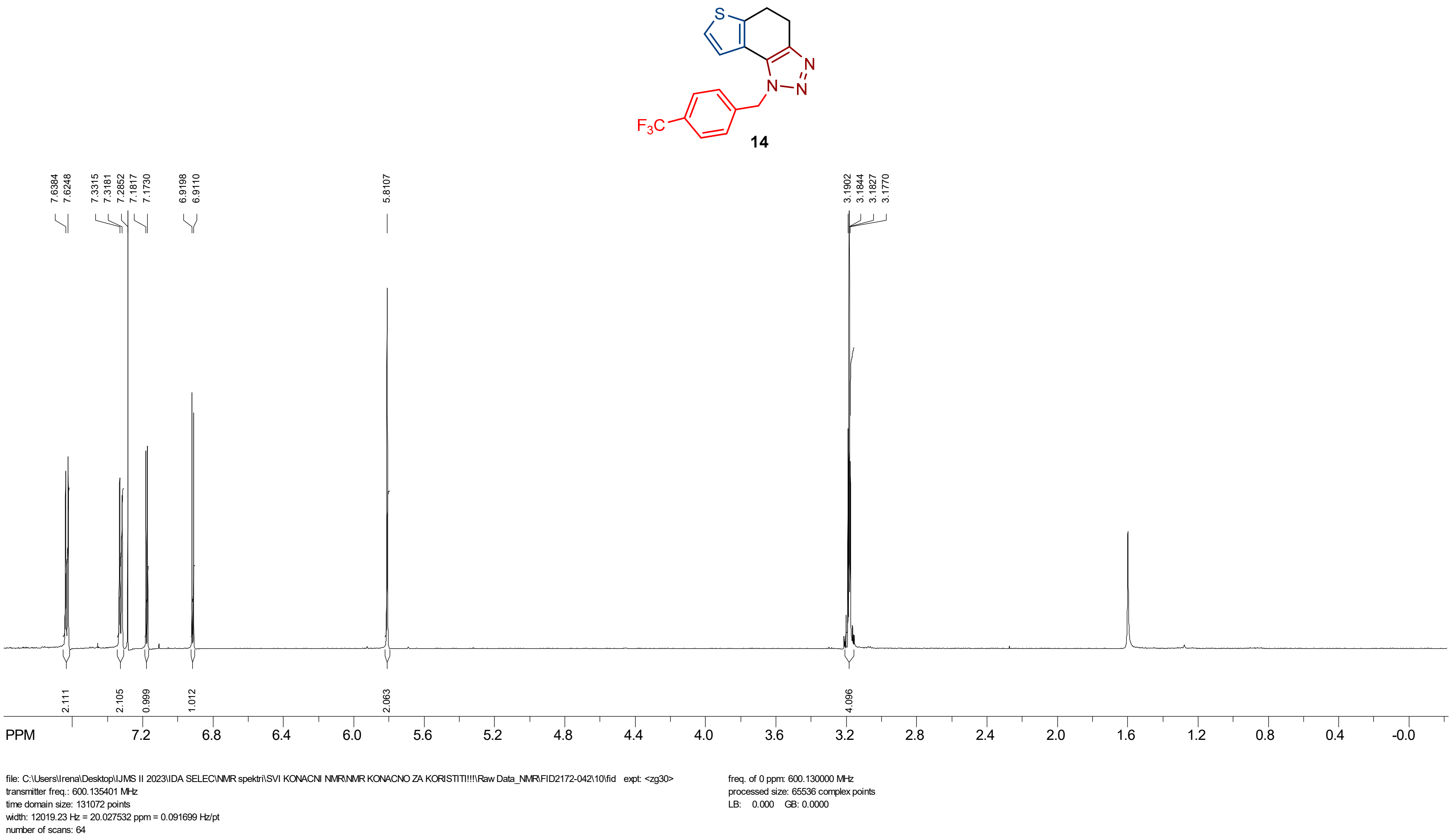

Figure S39. ^{1}H NMR spectrum ($CDCl_3$) of compound **14**.

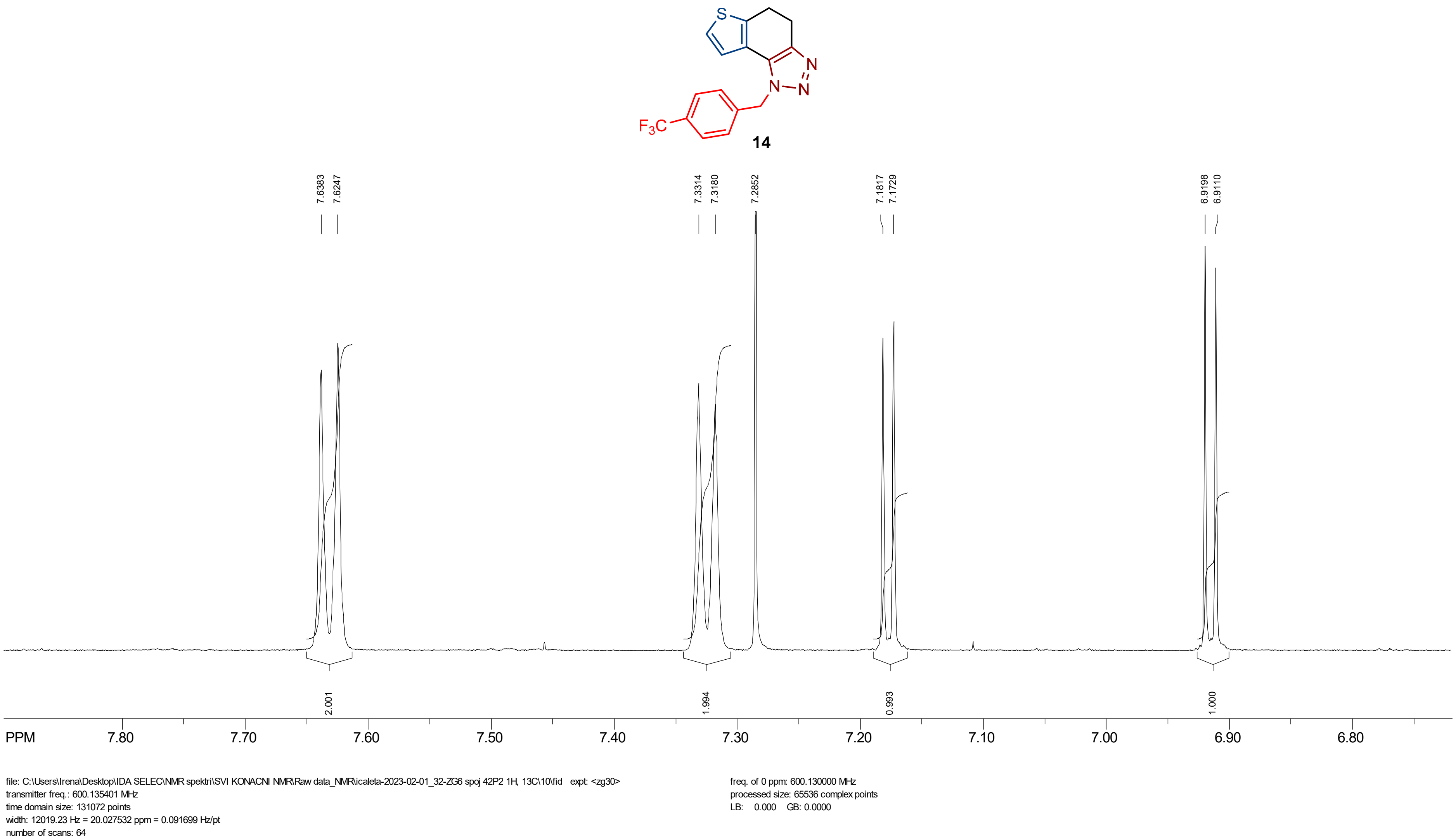

Figure S40. Aromatic part of the ^{1}H NMR spectrum ($CDCl_3$) of compound **14**.

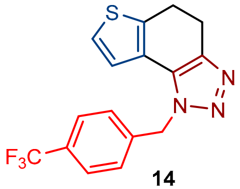

14

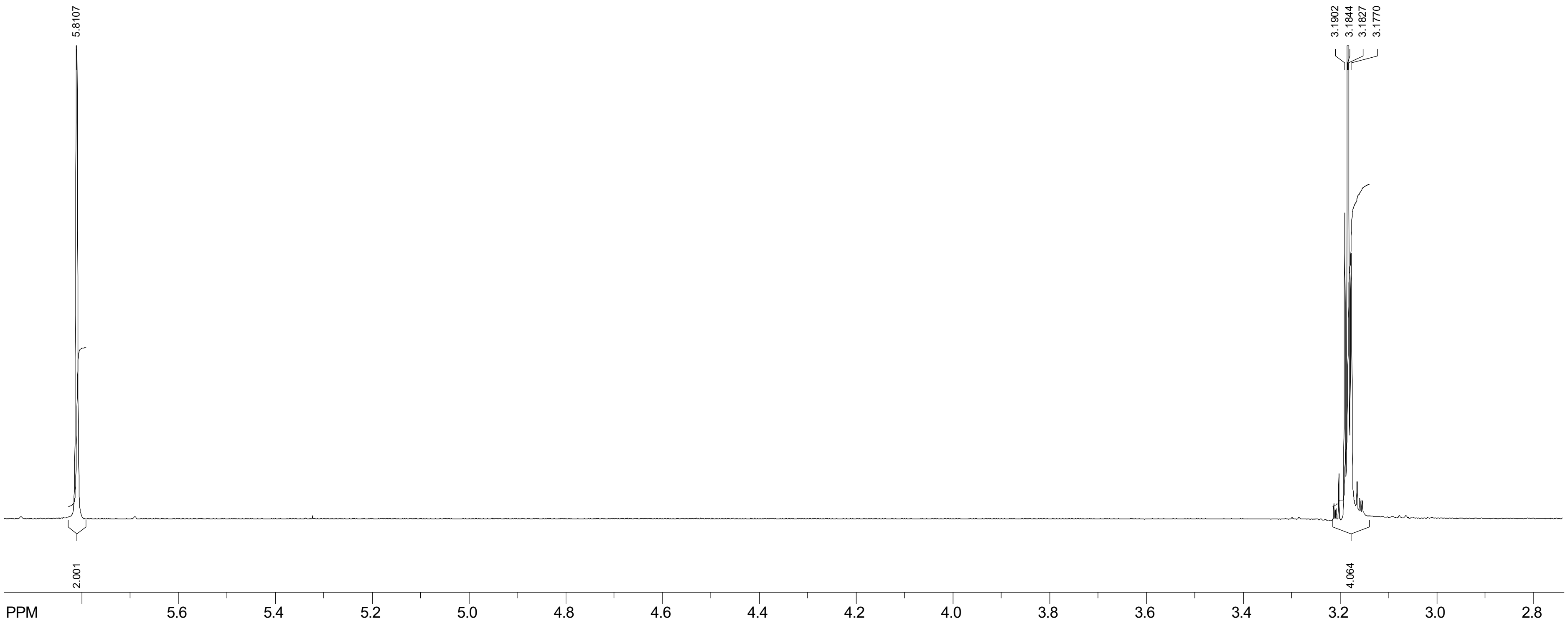

file: C:\Users\Irena\Desktop\IDA SELEC\NMR spektri\SVI KONACNI NMR\Raw data_NMR\icaleta-2023-02-01_32-ZG6 spoj 42P2 1H, 13C\10\fid expt: <zg30>
transmitter freq.: 600.135401 MHz
time domain size: 131072 points
width: 12019.23 Hz = 20.027532 ppm = 0.091699 Hz/pt
number of scans: 64

freq. of 0 ppm: 600.130000 MHz
processed size: 65536 complex points
LB: 0.000 GB: 0.0000

Figure S41. Aliphatic part of the ^{1}H NMR spectrum ($CDCl_3$) of compound **14**.

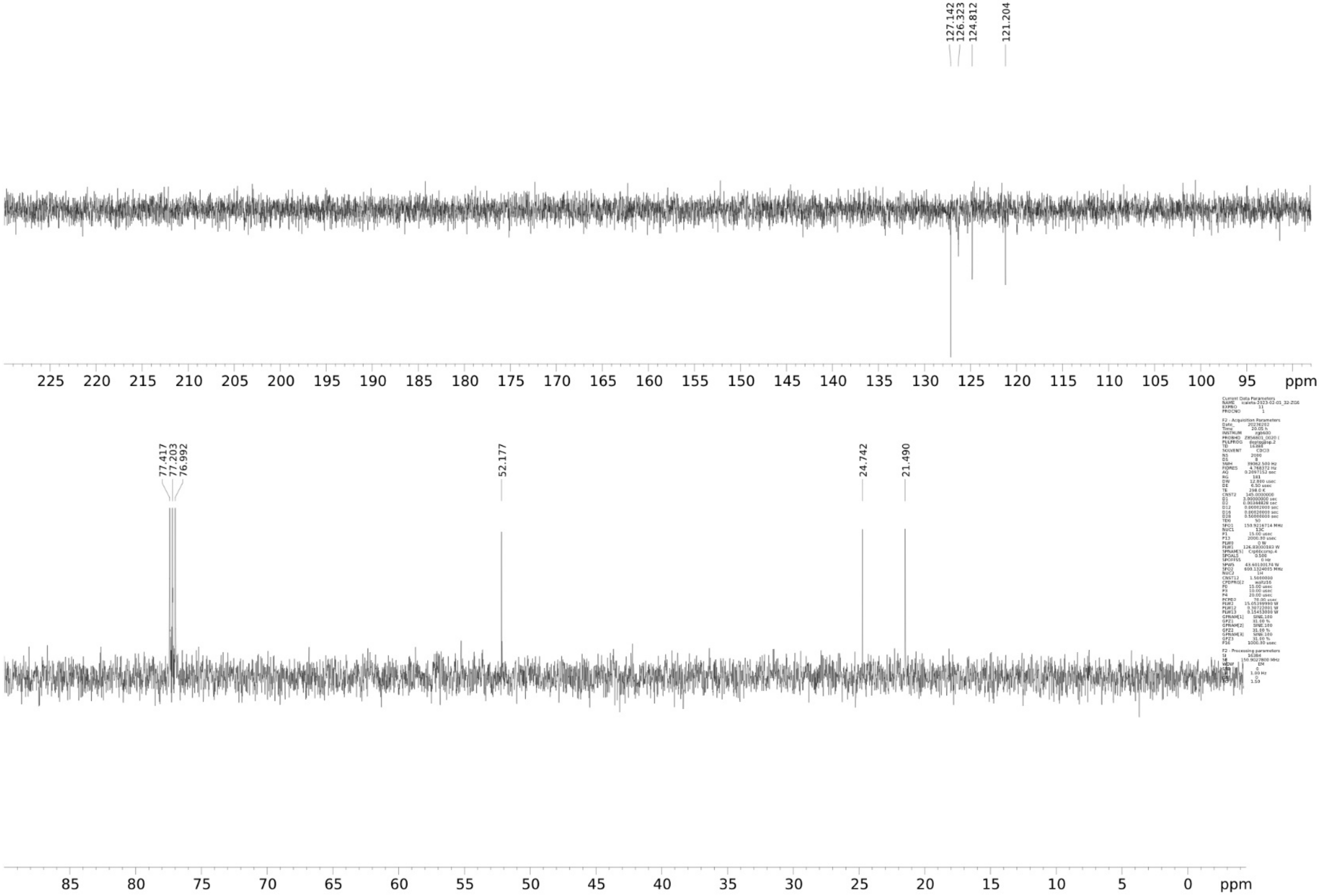

Figure S42. ^{13}C NMR spectrum ($CDCl_3$) of compound **14**.

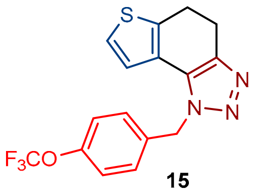

15

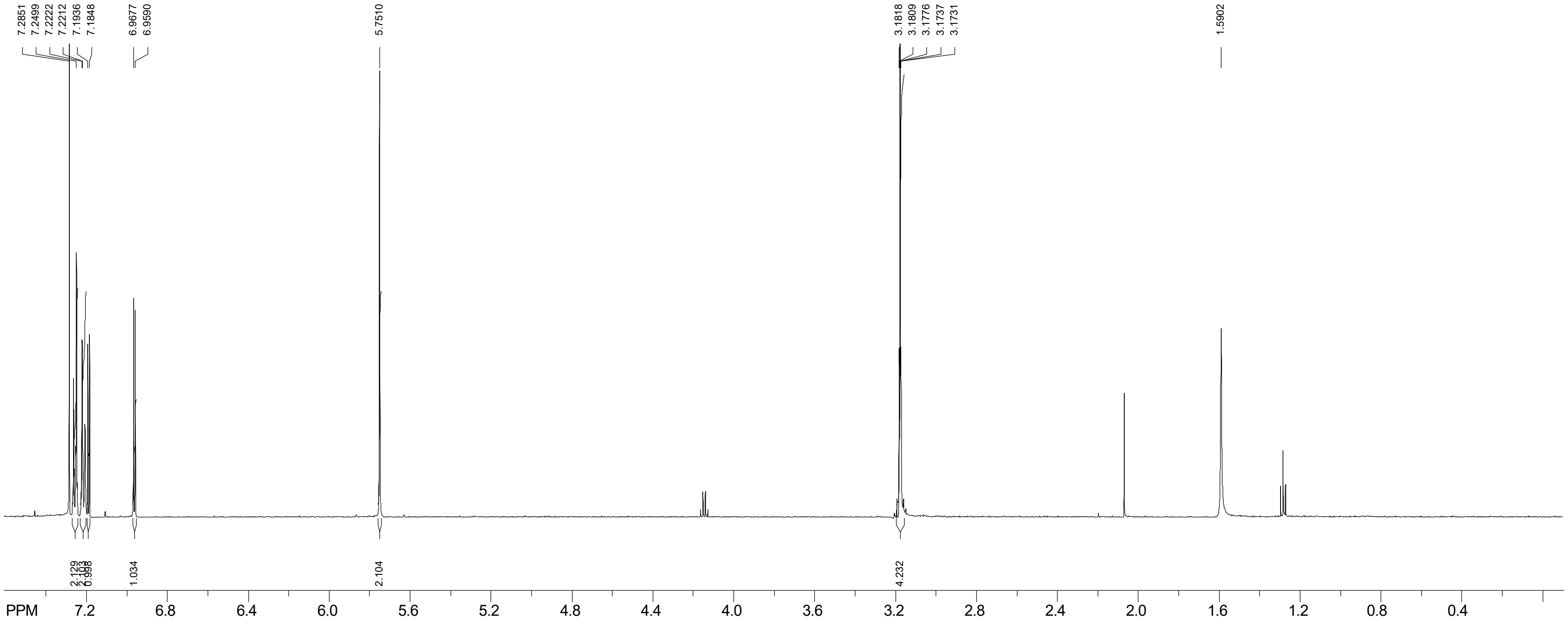

file: C:\Users\Irena\Desktop\IJMS II 2023\IDA SELEC\NMR spektri\SVI KONACNI NMR\NMR KONACNO ZA KORISTITI!!!\Raw Data_NMR\FID2172-027\10\fid expt: <zg30>
transmitter freq.: 600.135401 MHz
time domain size: 131072 points
width: 12019.23 Hz = 20.027532 ppm = 0.091699 Hz/pt
number of scans: 64

freq. of 0 ppm: 600.130000 MHz
processed size: 65536 complex points
LB: 0.000 GB: 0.0000

Figure S43. ^{1}H NMR spectrum ($CDCl_3$) of compound **15**.

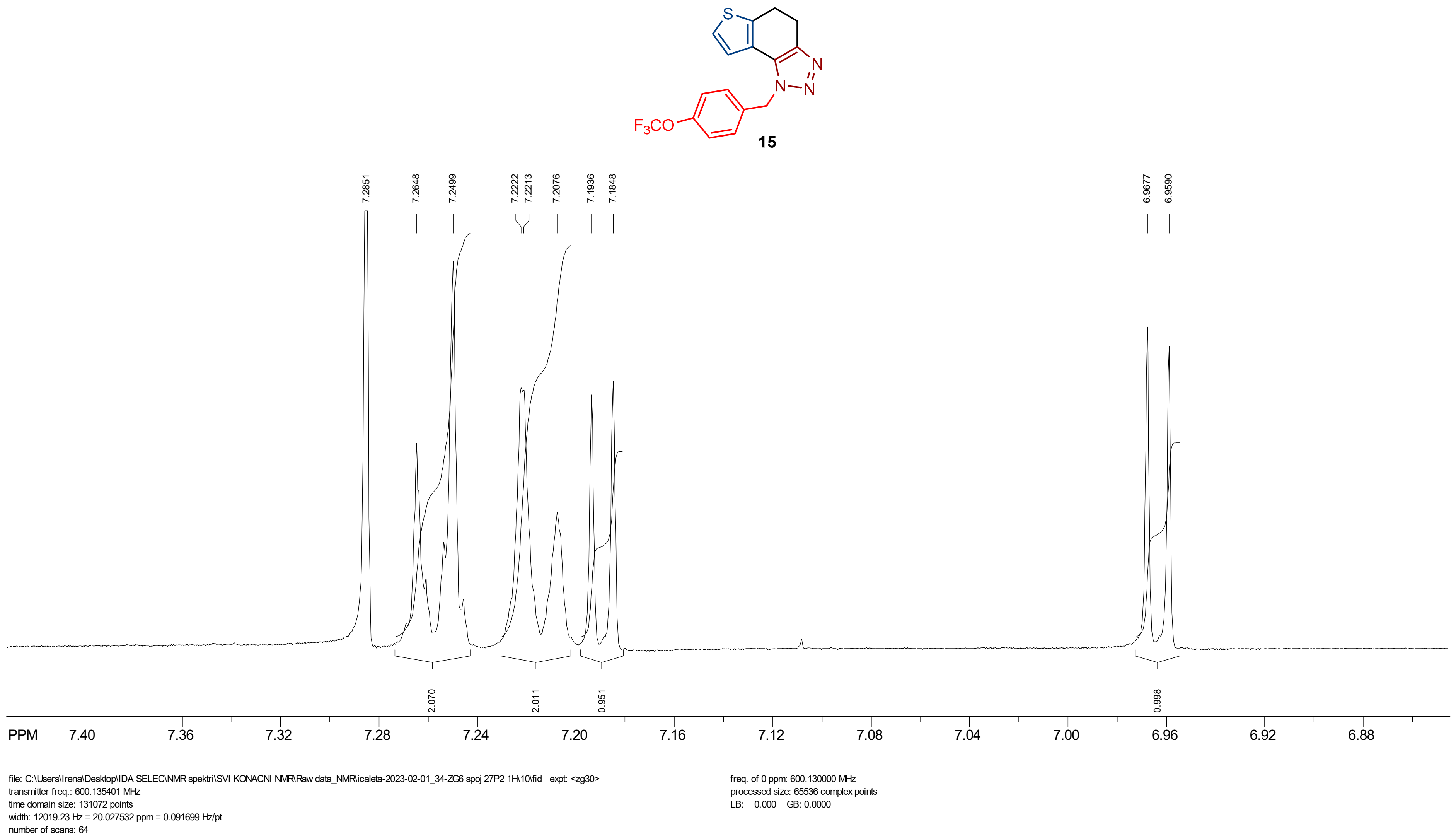

Figure S44. Aromatic part of the ^{1}H NMR spectrum ($CDCl_3$) of compound **15**.

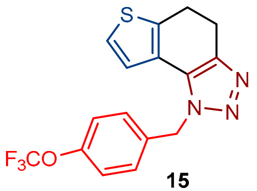

15

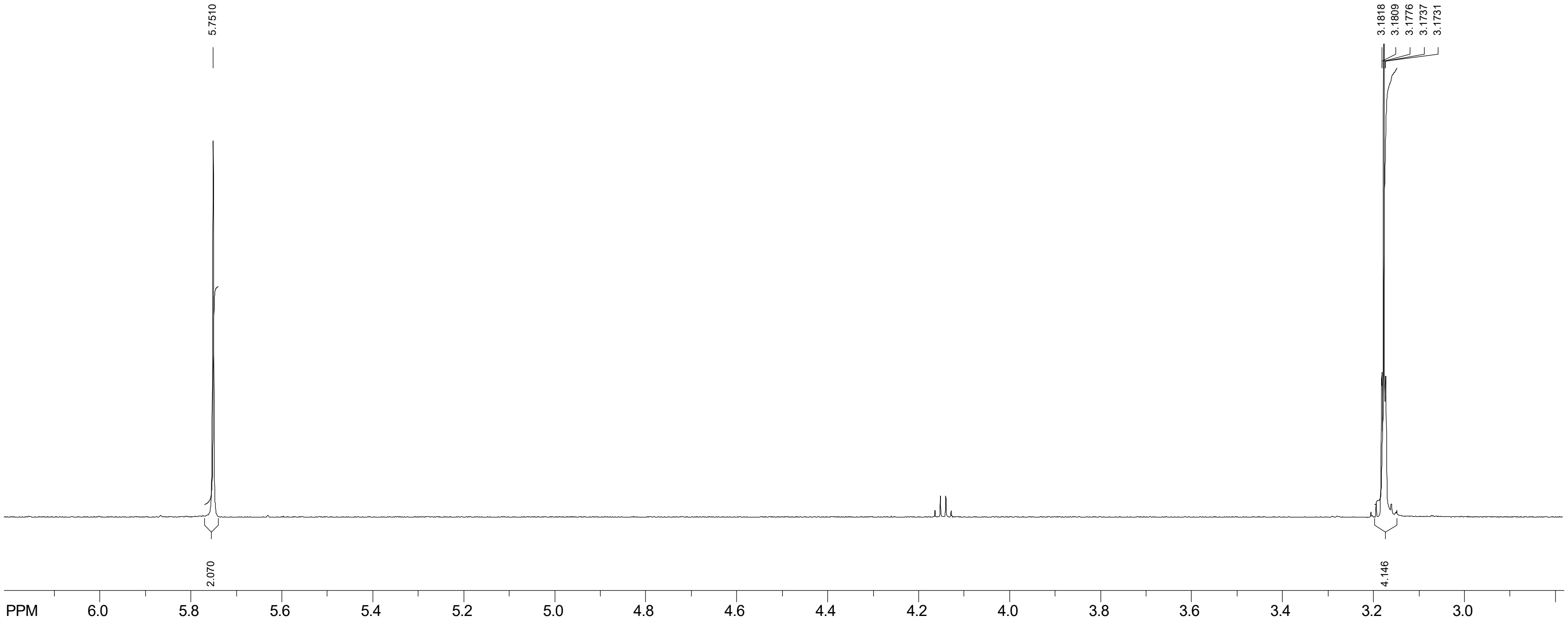

file: C:\Users\Irena\Desktop\IDA SELEC\NMR spektri\SVI KONACNI NMR\Raw data_NMR\icaleta-2023-02-01_34-ZG6 spoj 27P2 1H\10\fid expt: <zg30>
transmitter freq.: 600.135401 MHz
time domain size: 131072 points
width: 12019.23 Hz = 20.027532 ppm = 0.091699 Hz/pt
number of scans: 64

freq. of 0 ppm: 600.130000 MHz
processed size: 65536 complex points
LB: 0.000 GB: 0.0000

Figure S45. Aliphatic part of the ^{1}H NMR spectrum (CDCl$_3$) of compound **15**.

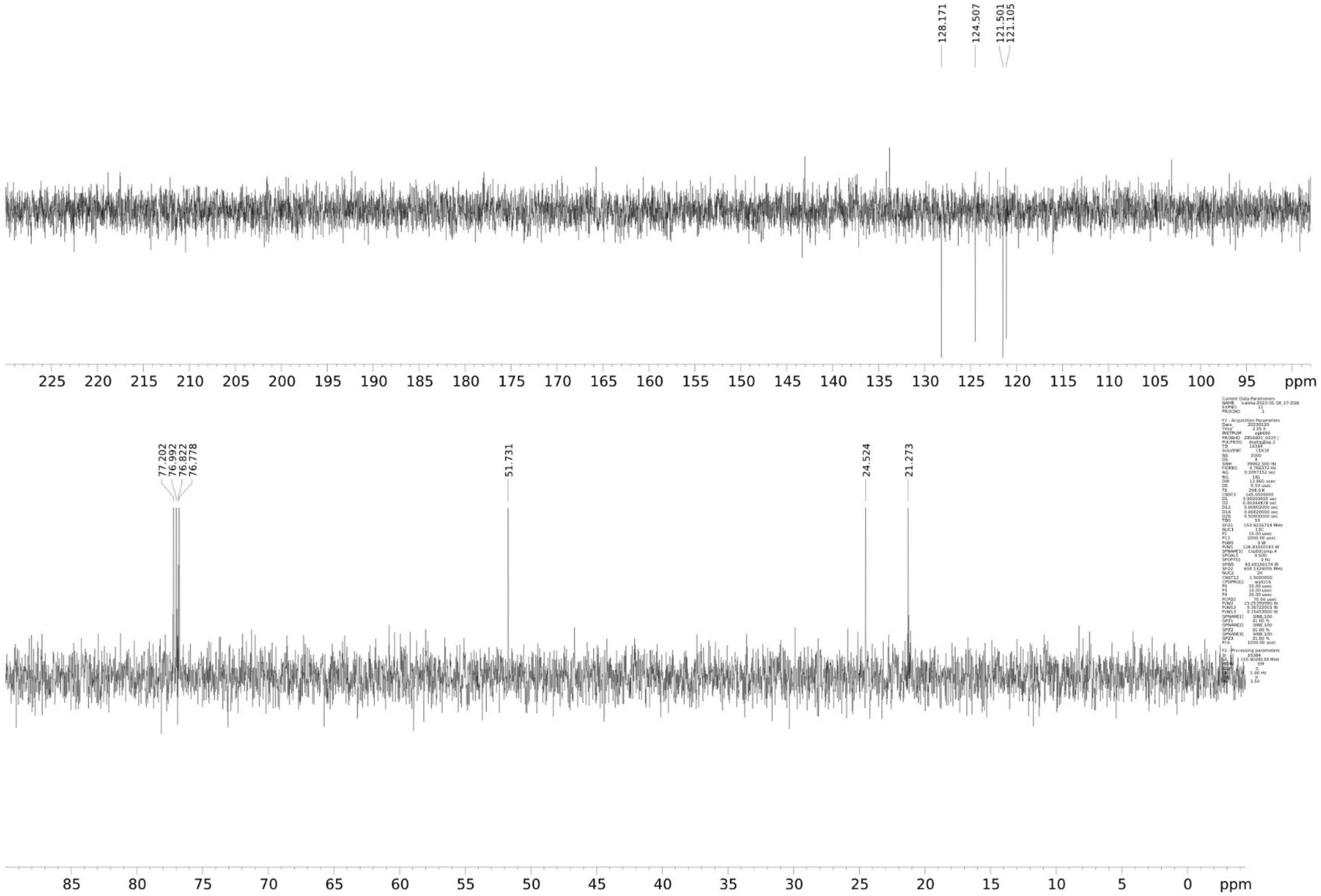

Figure S46. ^{13}C NMR spectrum ($CDCl_3$) of compound **15**.

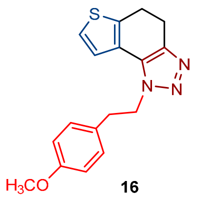

16

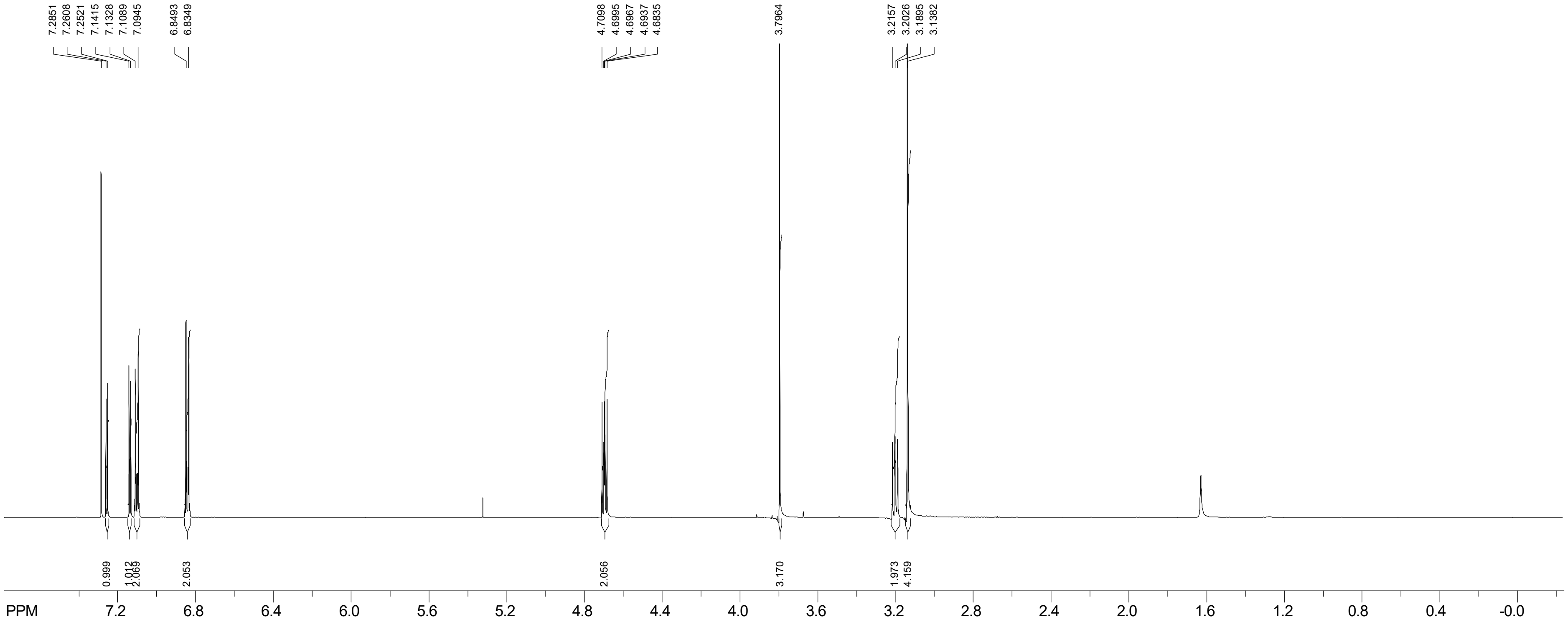

file: C:\Users\Irena\Desktop\IJMS II 2023\IDA SELEC\NMR spektri\SVI KONACNI NMR\NMR KONACNO ZA KORISTITI!!!\Raw Data_NMR\FID2172-029\10\fid expt: <zg30>
transmitter freq.: 600.135401 MHz
time domain size: 131072 points
width: 12019.23 Hz = 20.027532 ppm = 0.091699 Hz/pt
number of scans: 64

freq. of 0 ppm: 600.130000 MHz
processed size: 65536 complex points
LB: 0.000 GB: 0.0000

Figure S47. ^{1}H NMR spectrum ($CDCl_3$) of compound **16**.

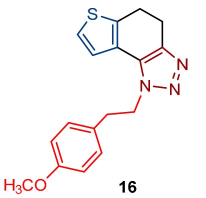

16

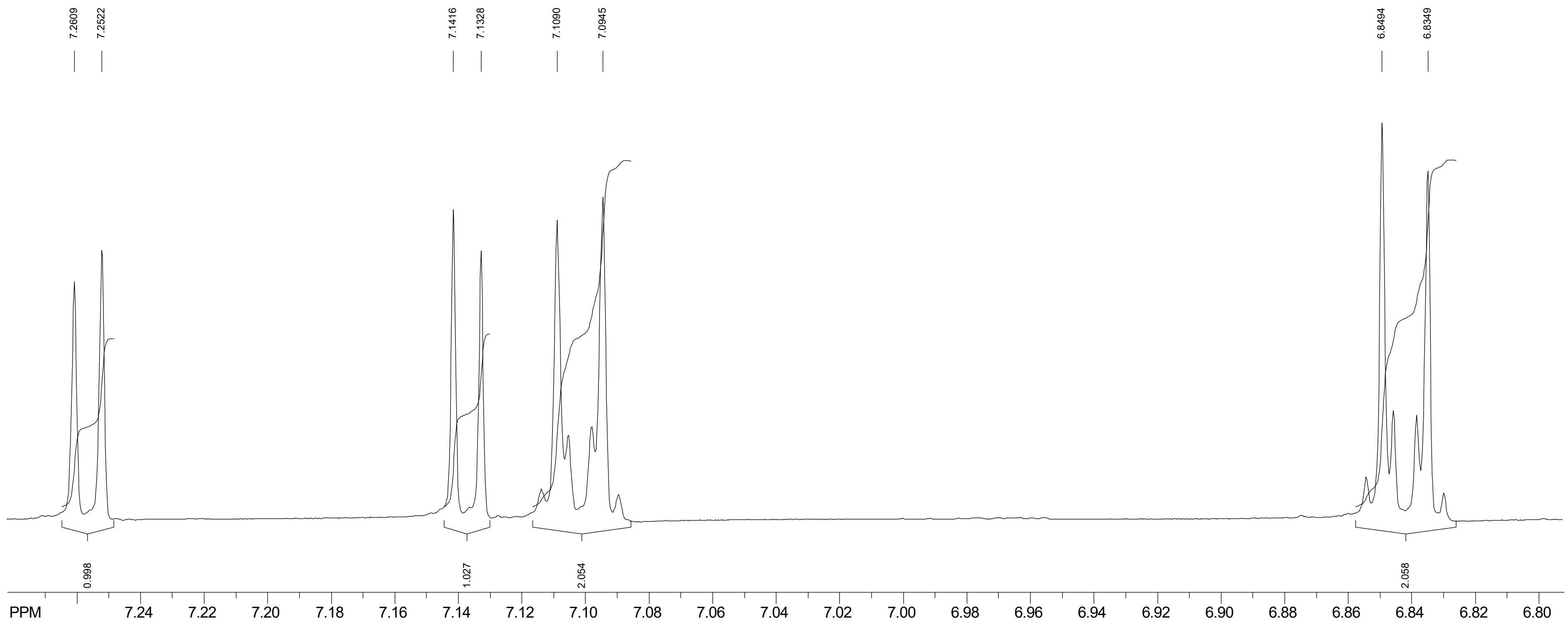

file: C:\Users\Irena\Desktop\IDA SELEC\NMR spektri\SVI KONACNI NMR\Raw data_NMR\icaleta-2023-02-01_38-ZG6 spoj 29P2 1H\10\fid expt: <zg30>
transmitter freq.: 600.135401 MHz
time domain size: 131072 points
width: 12019.23 Hz = 20.027532 ppm = 0.091699 Hz/pt
number of scans: 64

freq. of 0 ppm: 600.130000 MHz
processed size: 65536 complex points
LB: 0.000 GB: 0.0000

Figure S48. Aromatic part of the ^{1}H NMR spectrum ($CDCl_3$) of compound **16**.

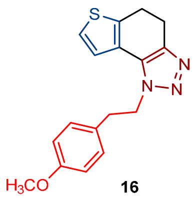

16

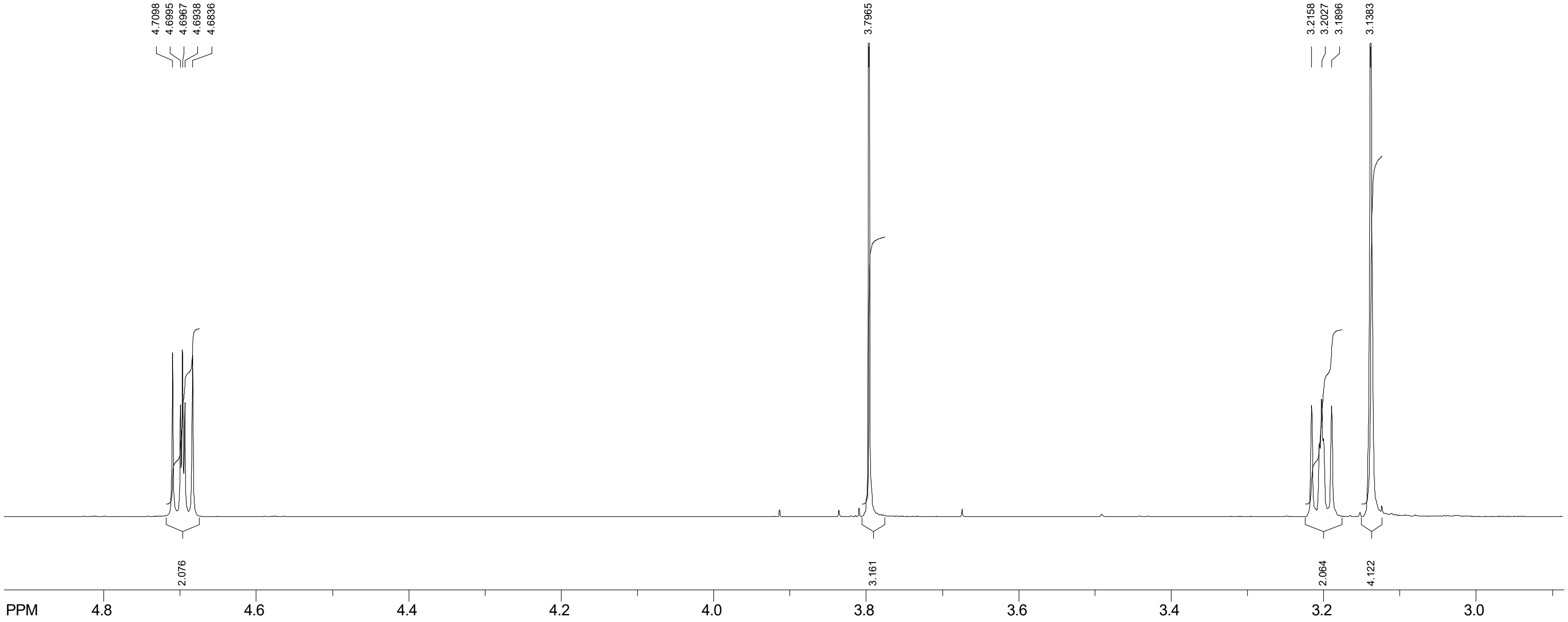

file: C:\Users\Irena\Desktop\IDA SELEC\NMR spektri\SVI KONACNI NMR\Raw data_NMR\icaleta-2023-02-01_38-ZG6 spoj 29P2 1H\10\fid expt: <zg30>
transmitter freq.: 600.135401 MHz
time domain size: 131072 points
width: 12019.23 Hz = 20.027532 ppm = 0.091699 Hz/pt
number of scans: 64

freq. of 0 ppm: 600.130000 MHz
processed size: 65536 complex points
LB: 0.000 GB: 0.0000

Figure S49. Aliphatic part of the ^{1}H NMR spectrum ($CDCl_3$) of compound **16**.

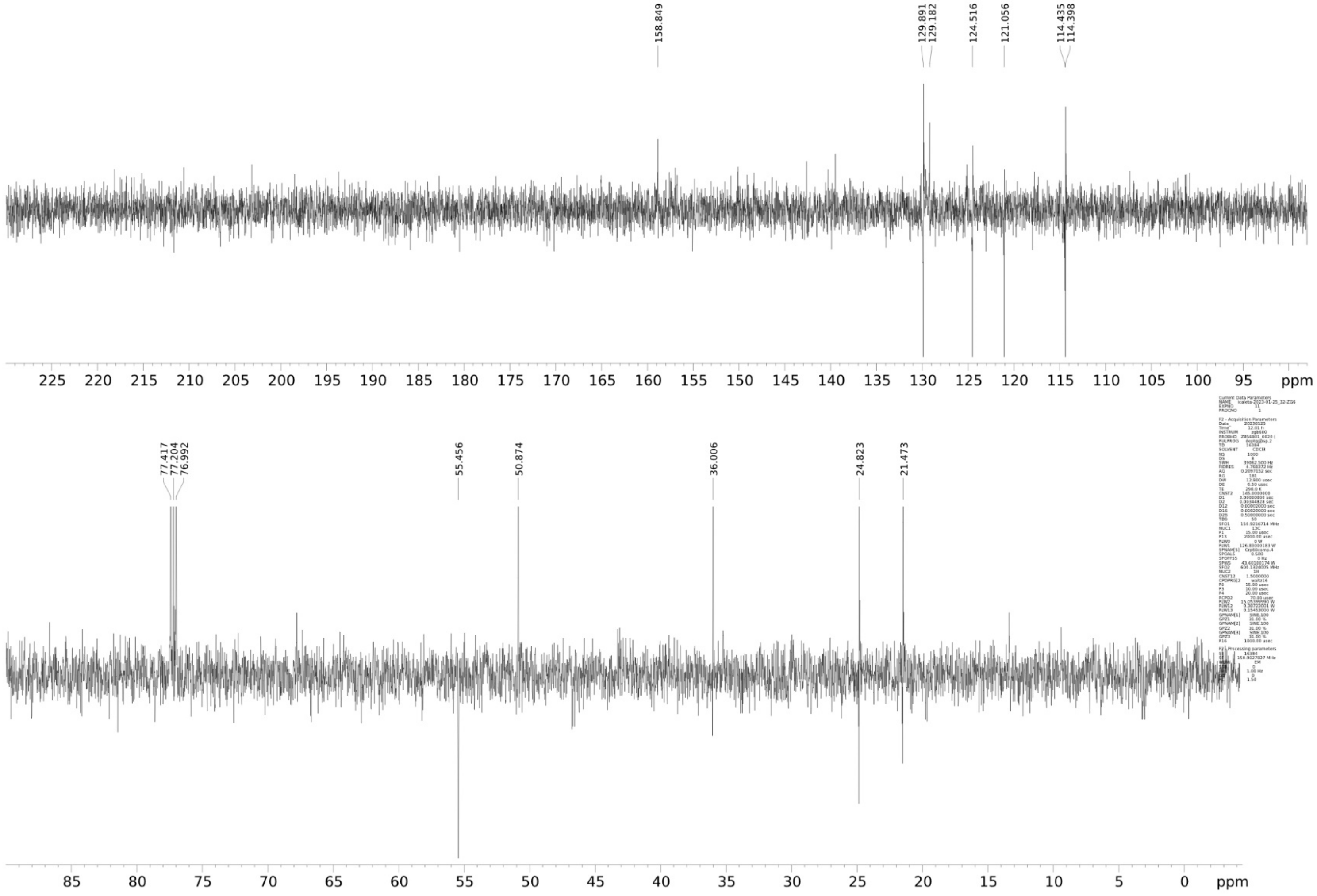

Figure S50. ^{13}C NMR spectrum ($CDCl_3$) of compound **16**.

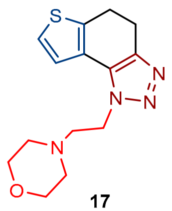

17

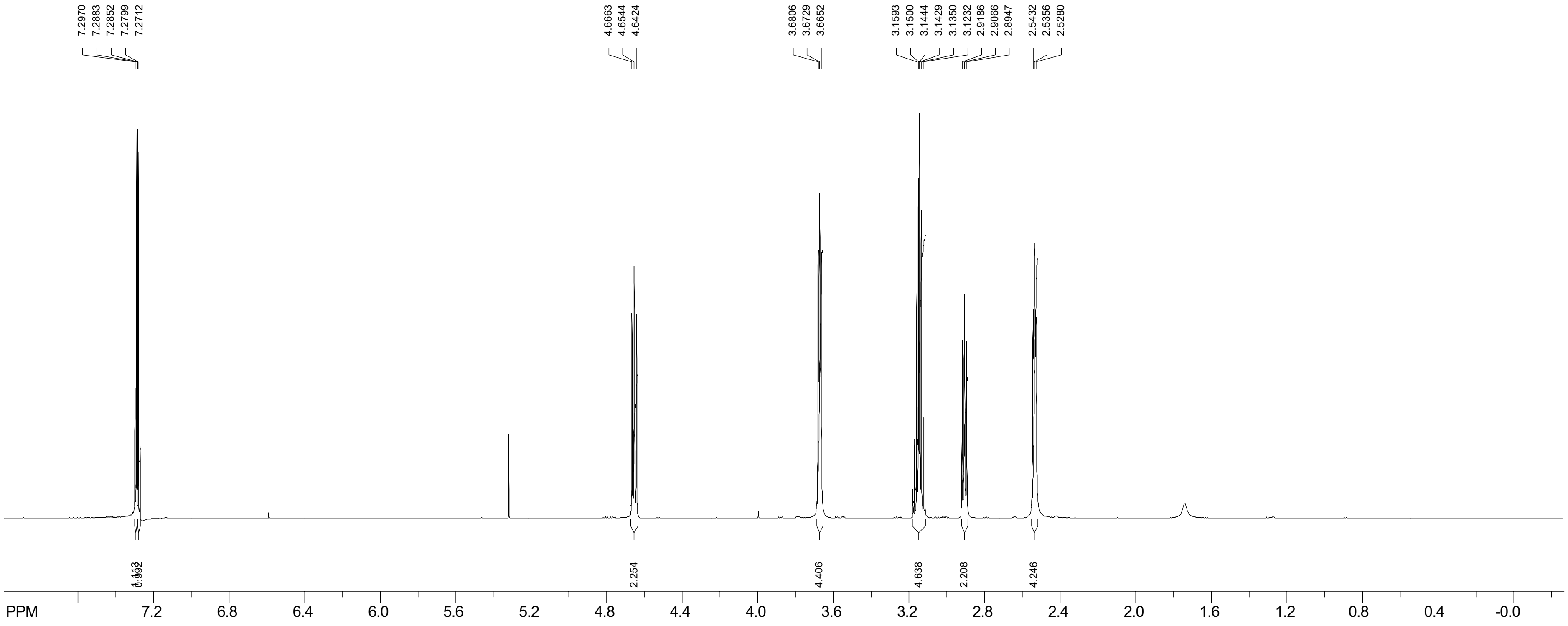

file: C:\Users\Irena\Desktop\IJMS II 2023\IDA SELEC\NMR spektri\SVI KONACNI NMR\NMR KONACNO ZA KORISTITI!!!\Raw Data_NMR\FID2172-041\10\fid expt: <zg30>
transmitter freq.: 600.135401 MHz
time domain size: 131072 points
width: 12019.23 Hz = 20.027532 ppm = 0.091699 Hz/pt
number of scans: 64

freq. of 0 ppm: 600.130000 MHz
processed size: 65536 complex points
LB: 0.000 GB: 0.0000

Figure S51. ^{1}H NMR spectrum ($CDCl_3$) of compound **17**.

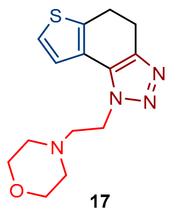

17

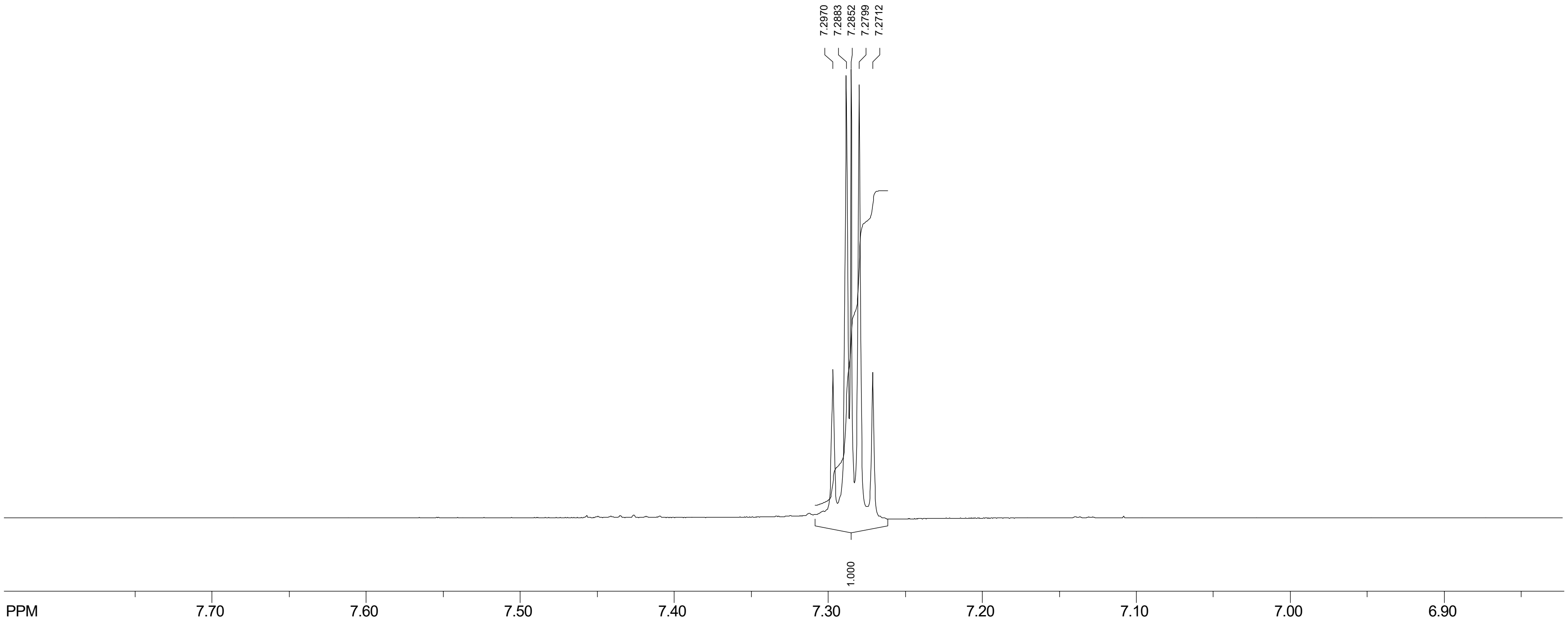

file: C:\Users\Irena\Desktop\IDA SELEC\NMR spektri\SVI KONACNI NMR\Raw data_NMR\icaleta-2023-02-01_28-ZG6 spoj 41P2 1H, 13C, COSY\10\fid expt: <zg30>
transmitter freq.: 600.135401 MHz
time domain size: 131072 points
width: 12019.23 Hz = 20.027532 ppm = 0.091699 Hz/pt
number of scans: 64

freq. of 0 ppm: 600.130000 MHz
processed size: 65536 complex points
LB: 0.000 GB: 0.0000

Figure S52. Aromatic part of the ^{1}H NMR spectrum ($CDCl_3$) of compound **17**.

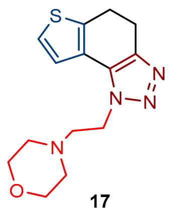

17

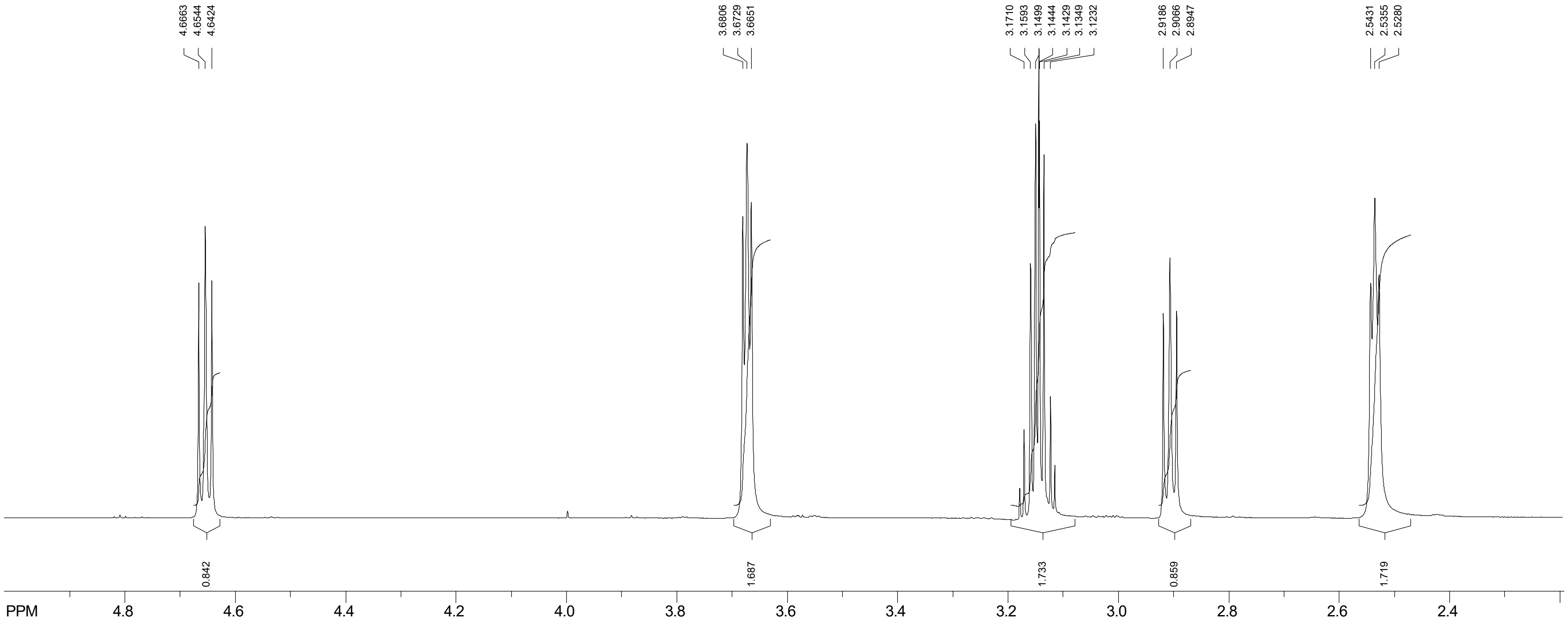

file: C:\Users\Irena\Desktop\IDA SELEC\NMR spektri\SVI KONACNI NMR\Raw data_NMR\icaleta-2023-02-01_28-ZG6 spoj 41P2 1H, 13C, COSY\10\fid expt: <zg30>
transmitter freq.: 600.135401 MHz
time domain size: 131072 points
width: 12019.23 Hz = 20.027532 ppm = 0.091699 Hz/pt
number of scans: 64

freq. of 0 ppm: 600.130000 MHz
processed size: 65536 complex points
LB: 0.000 GB: 0.0000

Figure S53. Aliphatic part of the ^{1}H NMR spectrum ($CDCl_3$) of compound **17**.

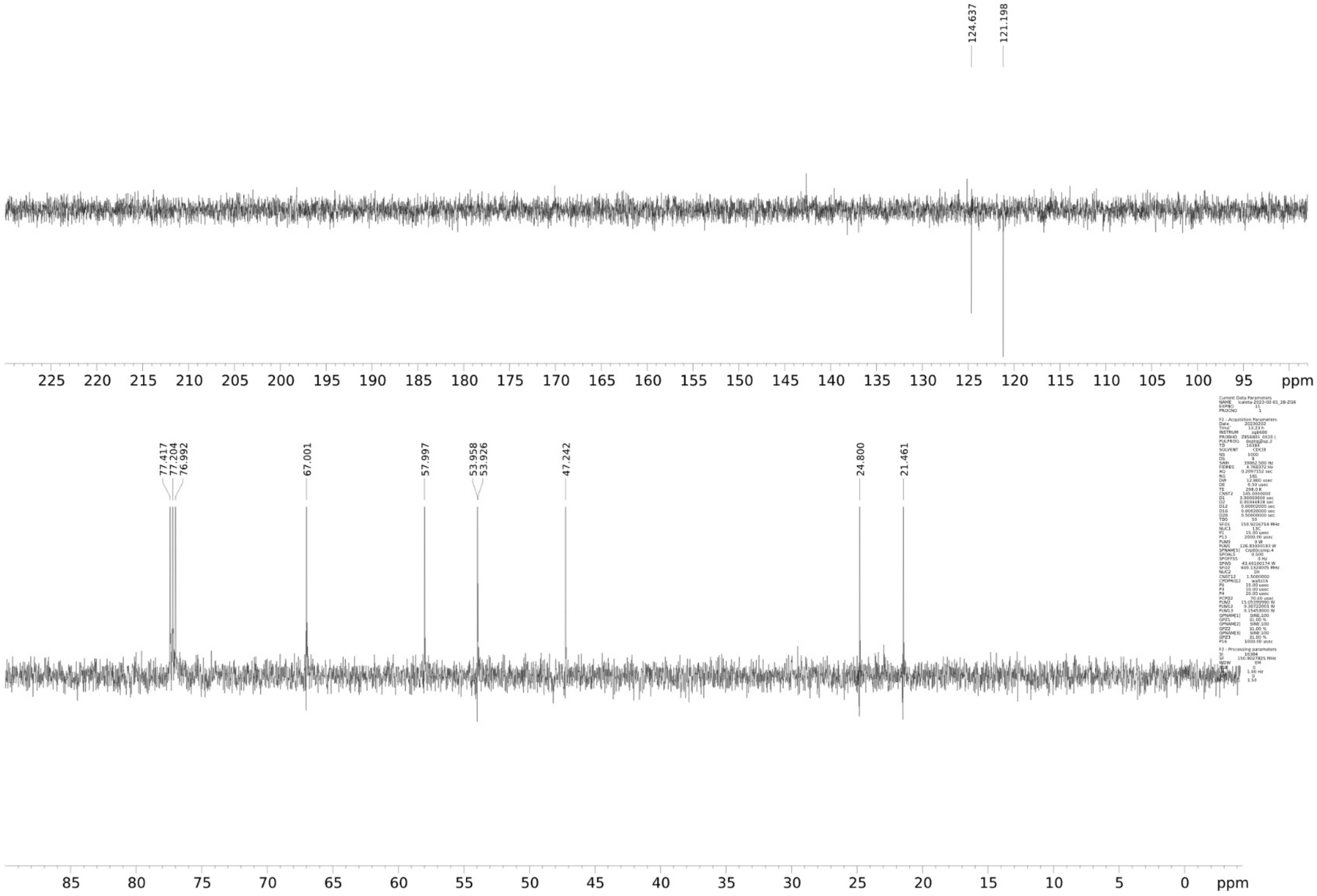

Figure S54. ^{13}C NMR spectrum ($CDCl_3$) of compound **17**.

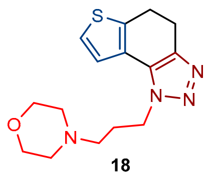

18

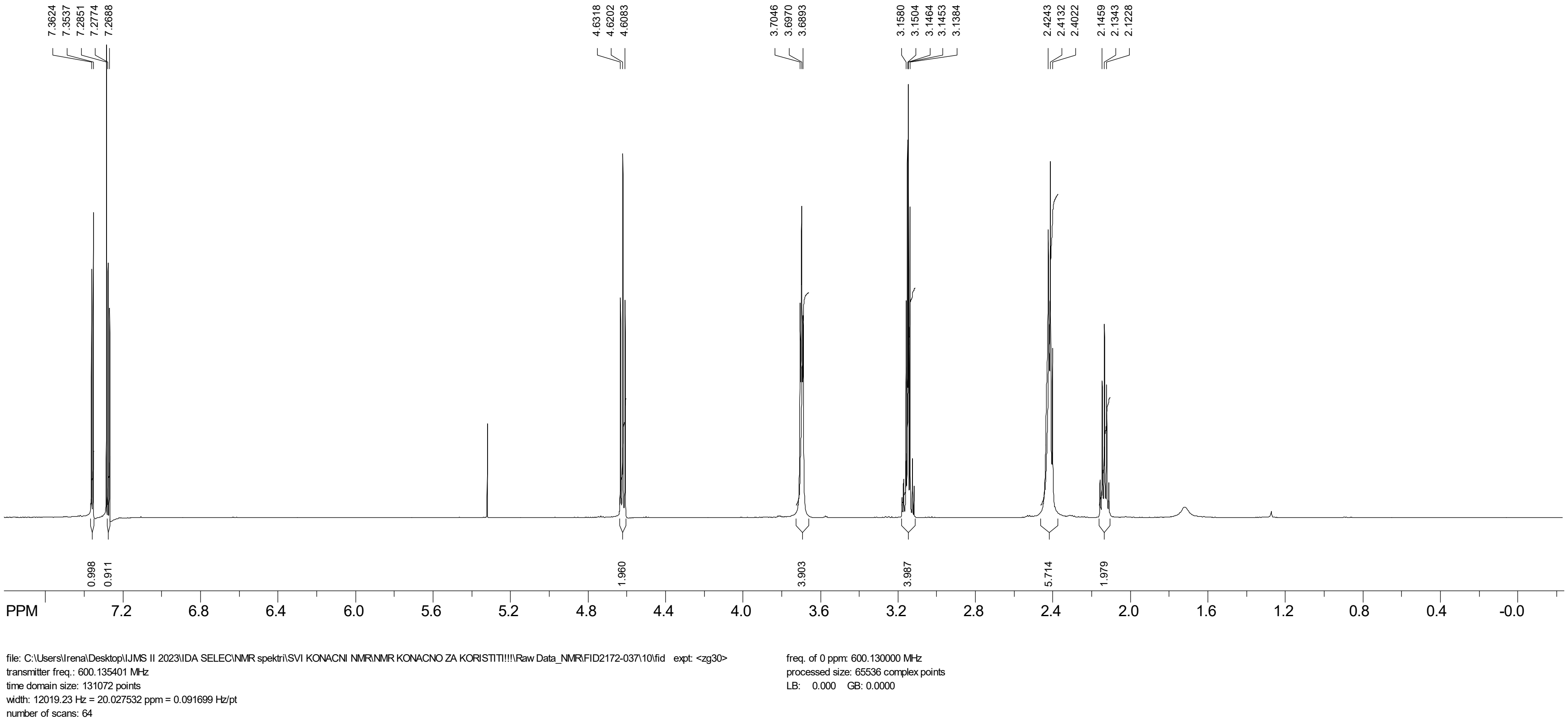

Figure S55. ^{1}H NMR spectrum ($CDCl_3$) of compound **18**.

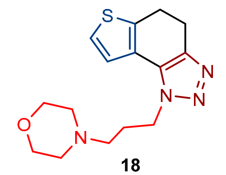

18

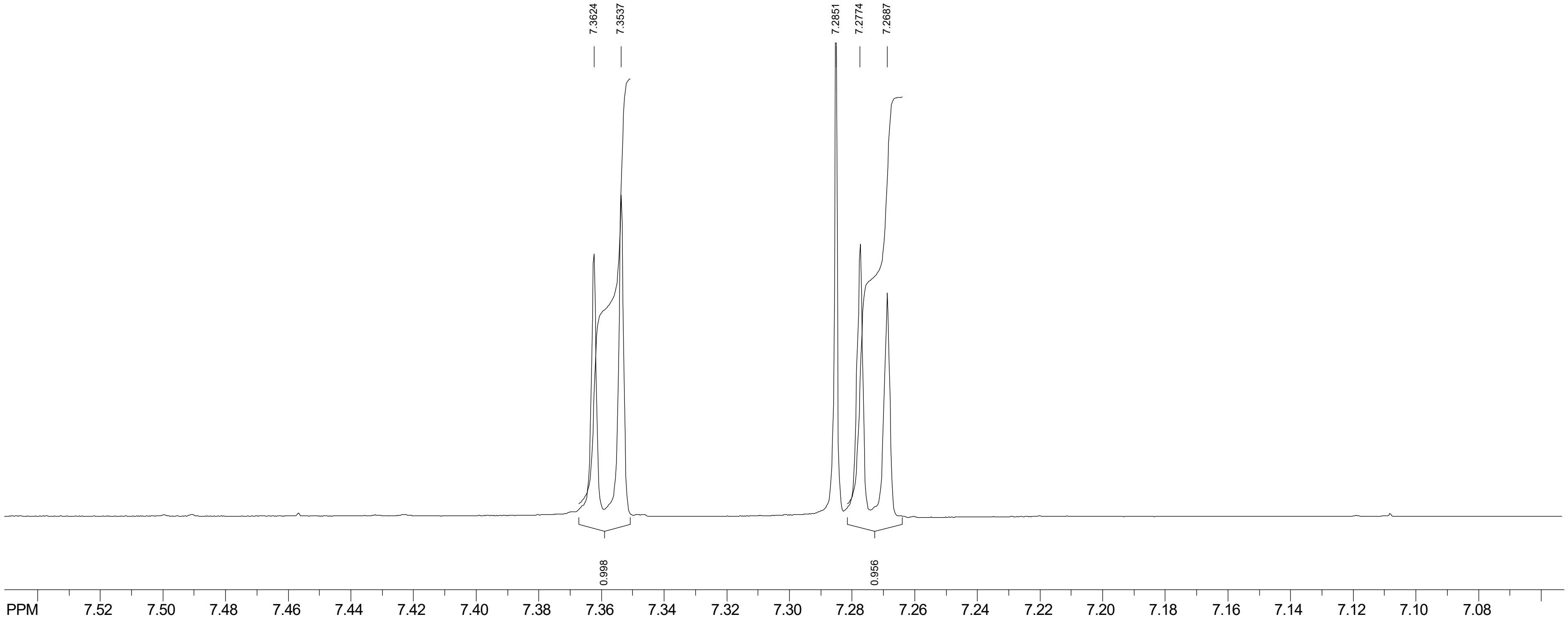

file: C:\Users\Irena\Desktop\IDA SELEC\NMR spektri\SVI KONACNI NMR\Raw data_NMR\icaleta-2023-02-01_30-ZG6 spoj 37P3 1H\10\fid expt: <zg30>
transmitter freq.: 600.135401 MHz
time domain size: 131072 points
width: 12019.23 Hz = 20.027532 ppm = 0.091699 Hz/pt
number of scans: 64

freq. of 0 ppm: 600.130000 MHz
processed size: 65536 complex points
LB: 0.000 GB: 0.0000

Figure S56. Aromatic part of the ^{1}H NMR spectrum ($CDCl_3$) of compound **18**.

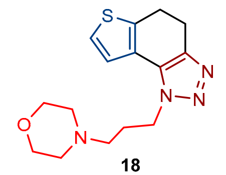

18

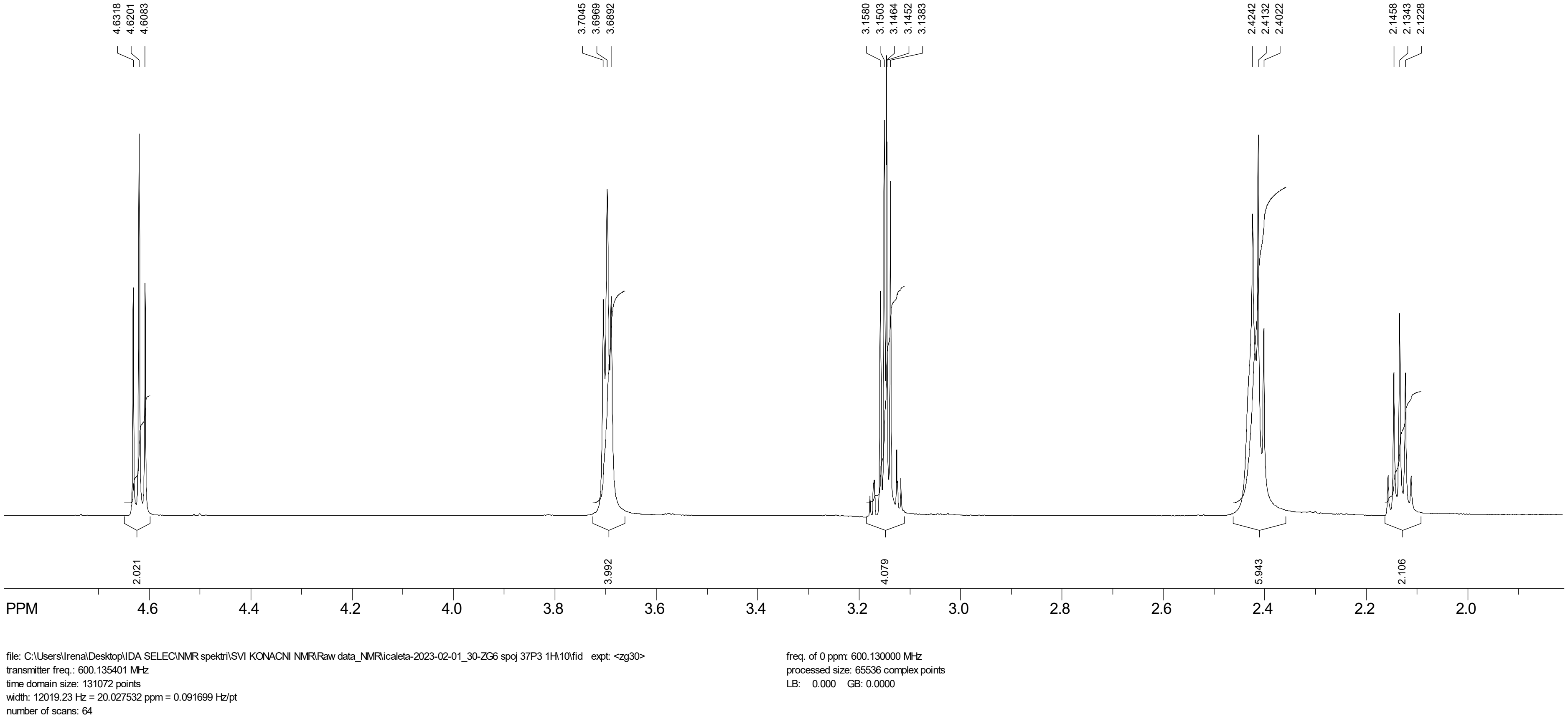

Figure S57. Aliphatic part of the ^{1}H NMR spectrum ($CDCl_3$) of compound **18**.

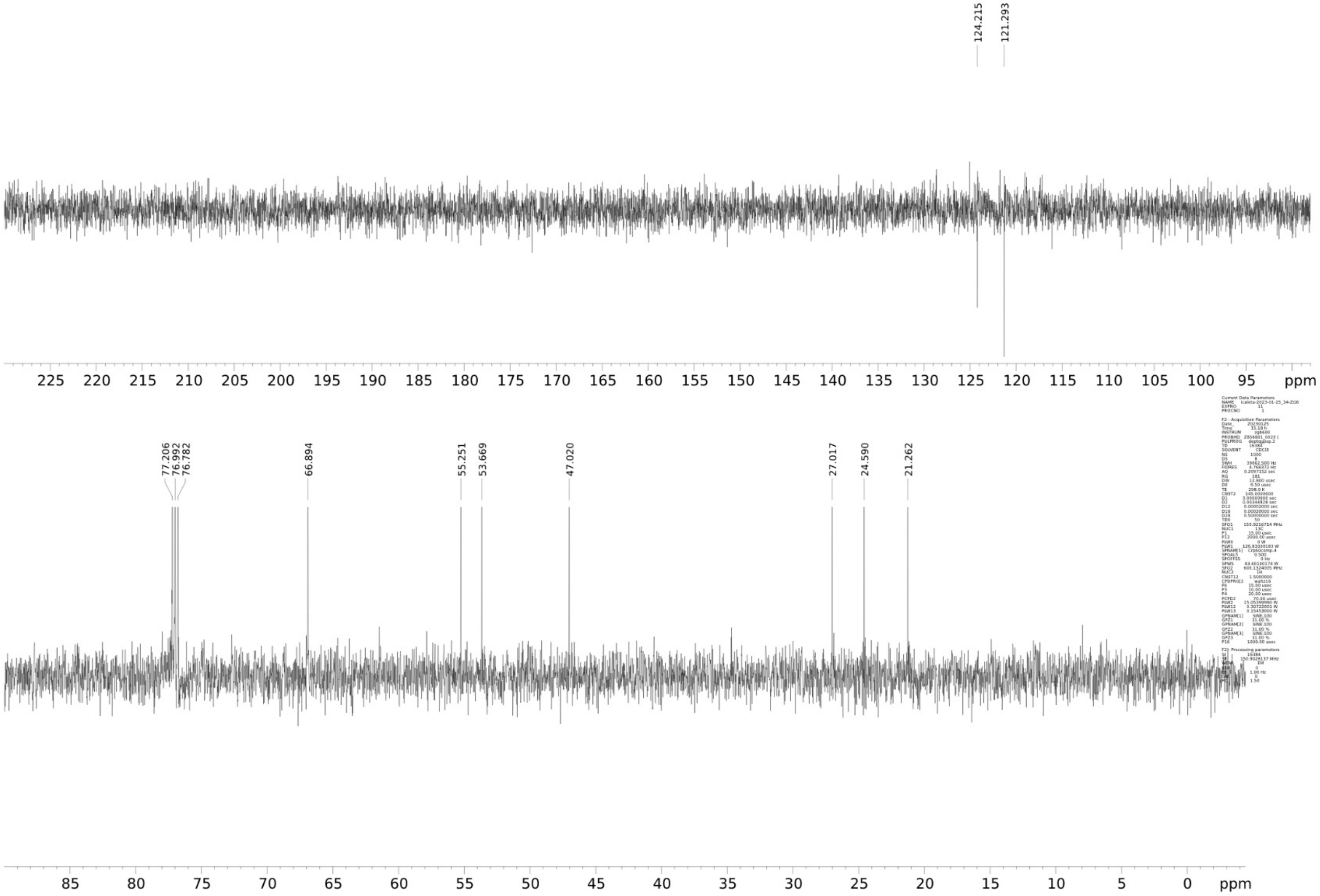

Figure S58. ^{13}C NMR spectrum ($CDCl_3$) of compound **18**.

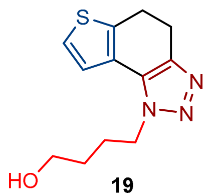

19

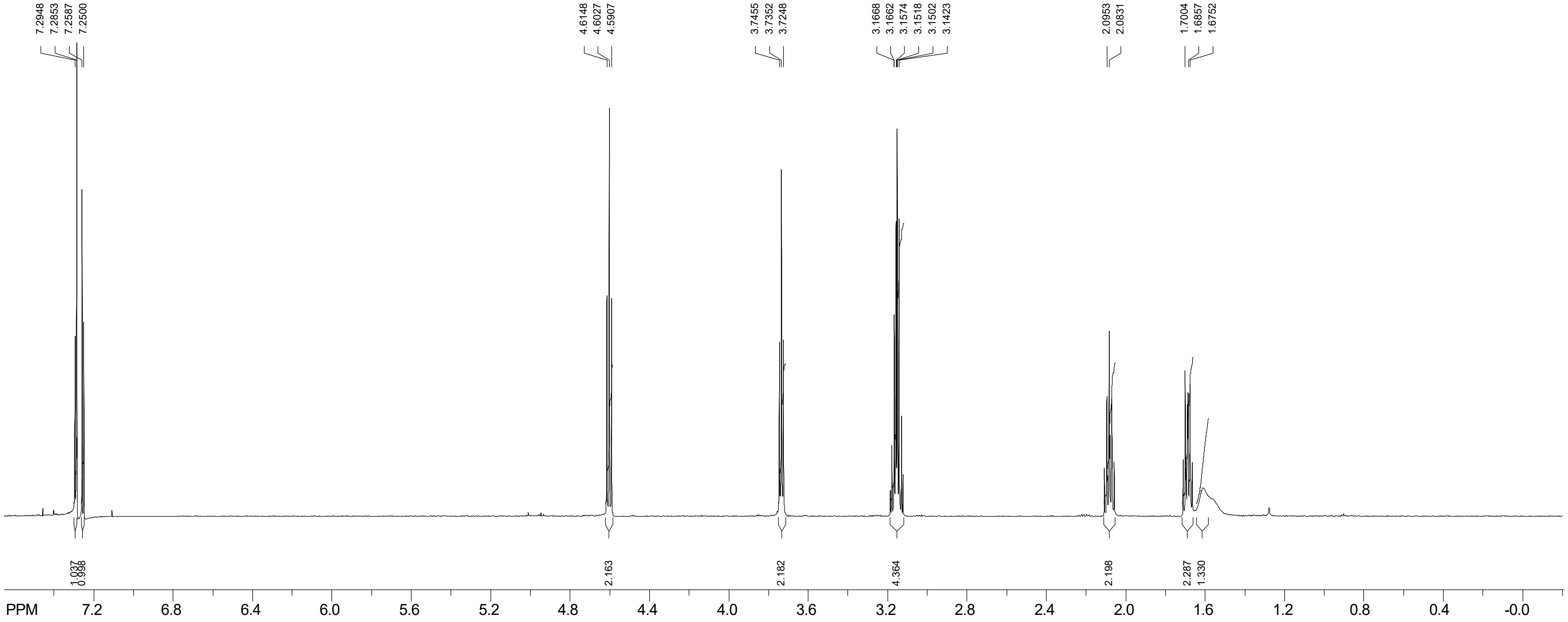

file: C:\Users\Irena\Desktop\IJMS II 2023\IDA SELEC\NMR spektri\SVI KONACNI NMR\NMR KONACNO ZA KORISTITI!!!\Raw Data_NMR\FID2172-031\10\fid expt: <zg30>
transmitter freq.: 600.135401 MHz
time domain size: 131072 points
width: 12019.23 Hz = 20.027532 ppm = 0.091699 Hz/pt
number of scans: 64

freq. of 0 ppm: 600.130000 MHz
processed size: 65536 complex points
LB: 0.000 GB: 0.0000

Figure S59. ^{1}H NMR spectrum ($CDCl_3$) of compound **19**.

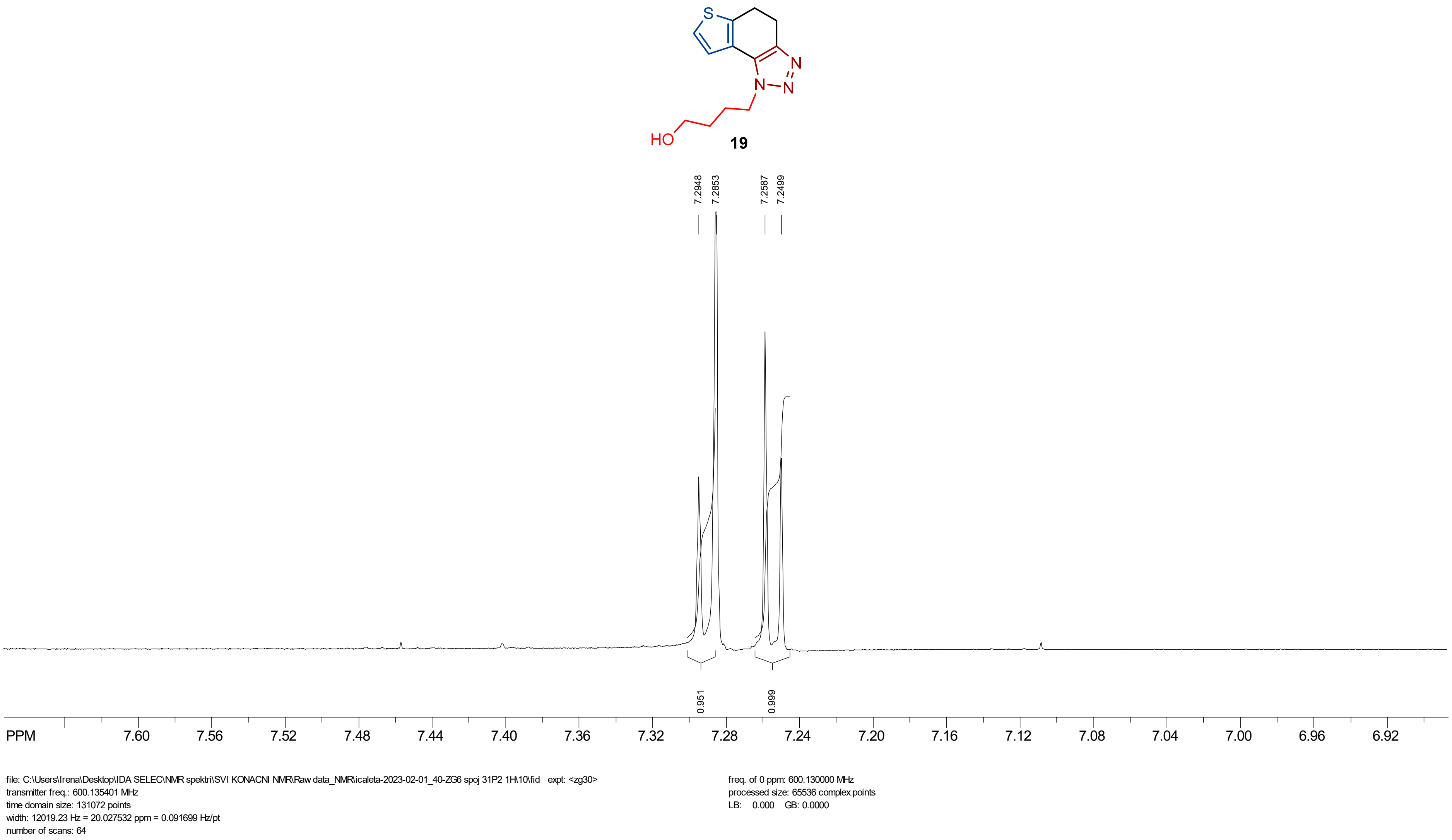

Figure S60. Aromatic part of the ^{1}H NMR spectrum ($CDCl_3$) of compound **19**.

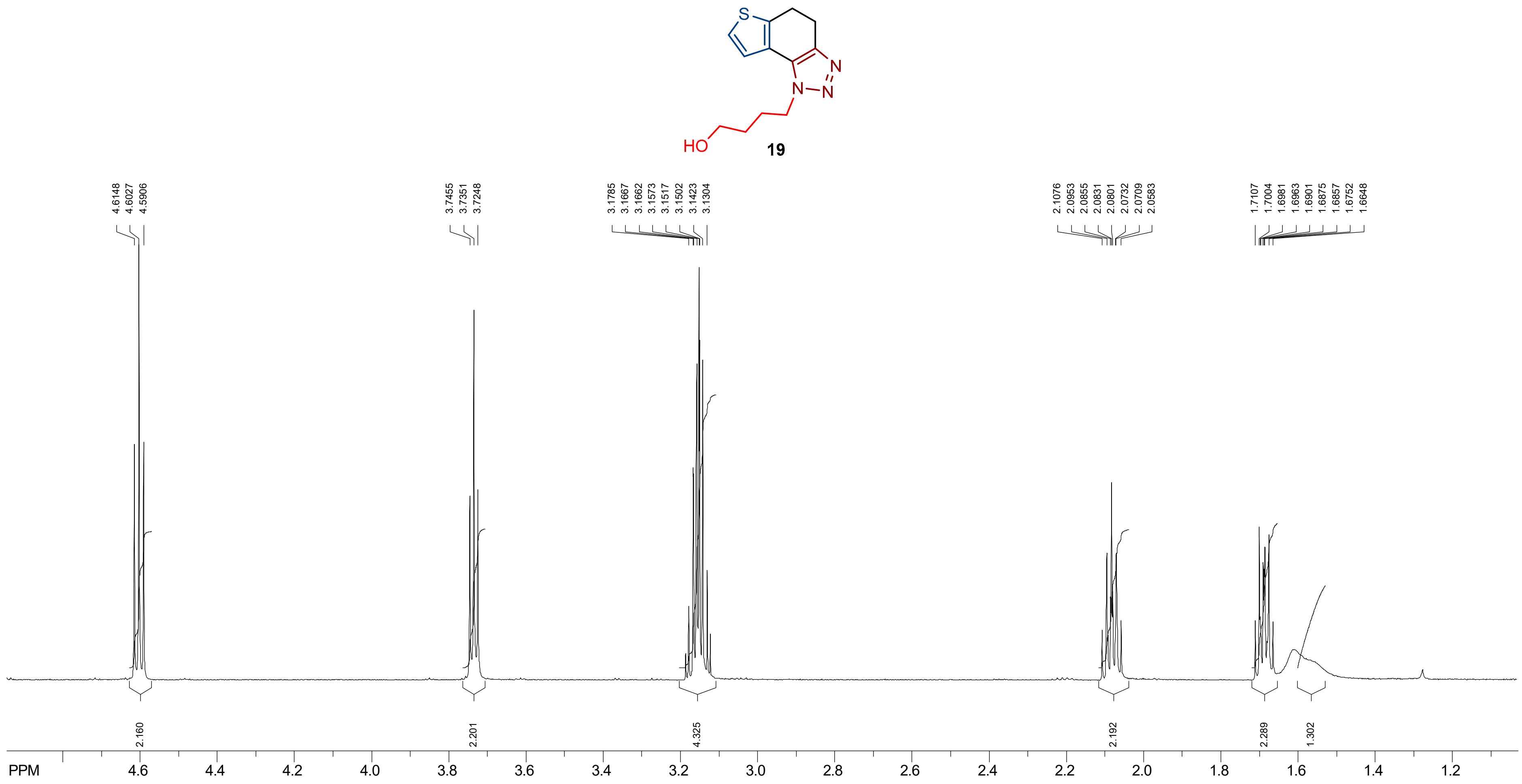

file: C:\Users\Irena\Desktop\IDA SELEC\NMR spektri\SVI KONACNI NMR\Raw data_NMR\icaleta-2023-02-01_40-ZG6 spoj 31P2 1H\10\fid expt: <zg30>
transmitter freq.: 600.135401 MHz
time domain size: 131072 points
width: 12019.23 Hz = 20.027532 ppm = 0.091699 Hz/pt
number of scans: 64

freq. of 0 ppm: 600.130000 MHz
processed size: 65536 complex points
LB: 0.000 GB: 0.0000

Figure S61. Aliphatic part of the ^{1}H NMR spectrum ($CDCl_3$) of compound **19**.

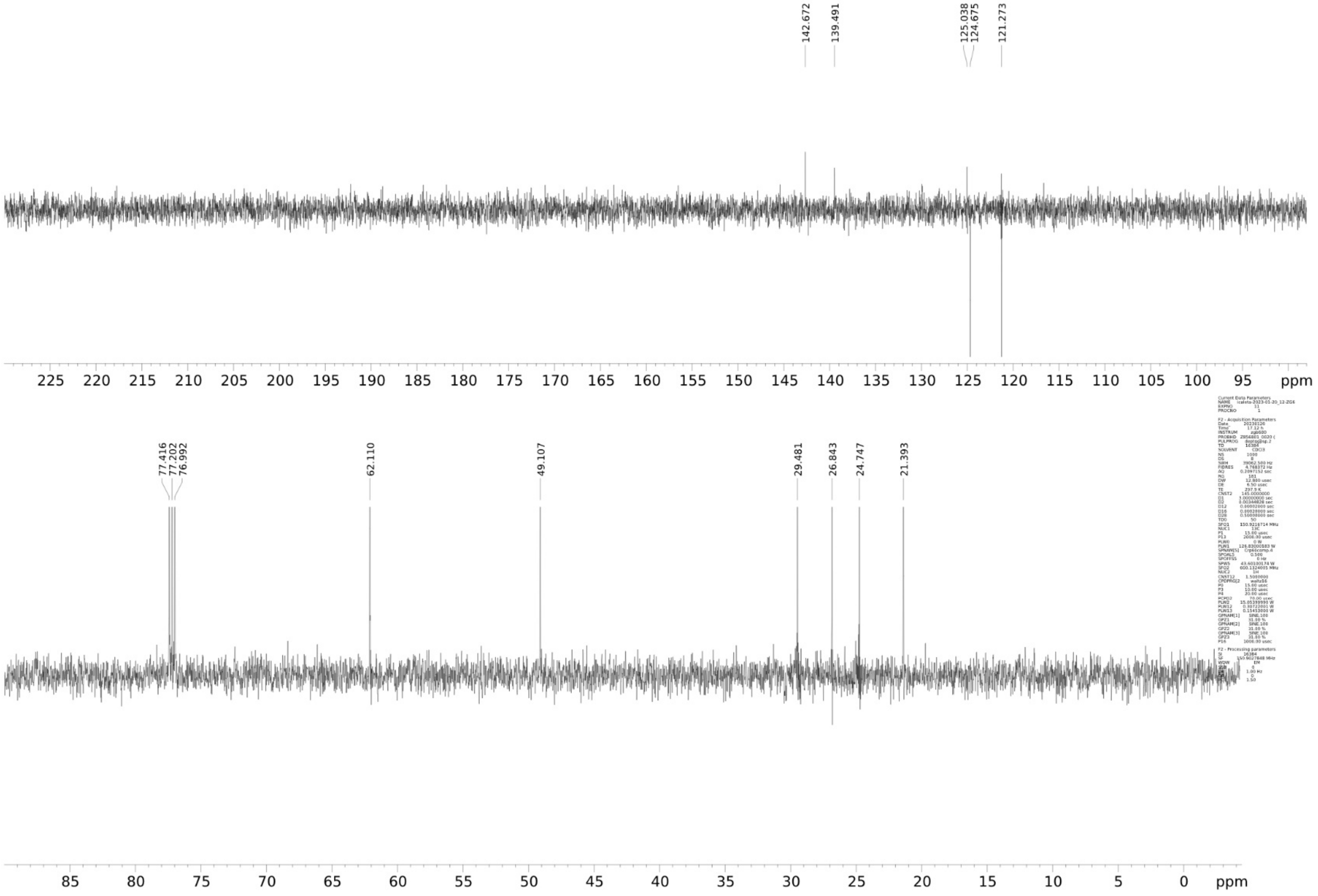

Figure S62. ^{13}C NMR spectrum ($CDCl_3$) of compound **19**.

2. MS spectra and HRMS analyses of synthesized compounds

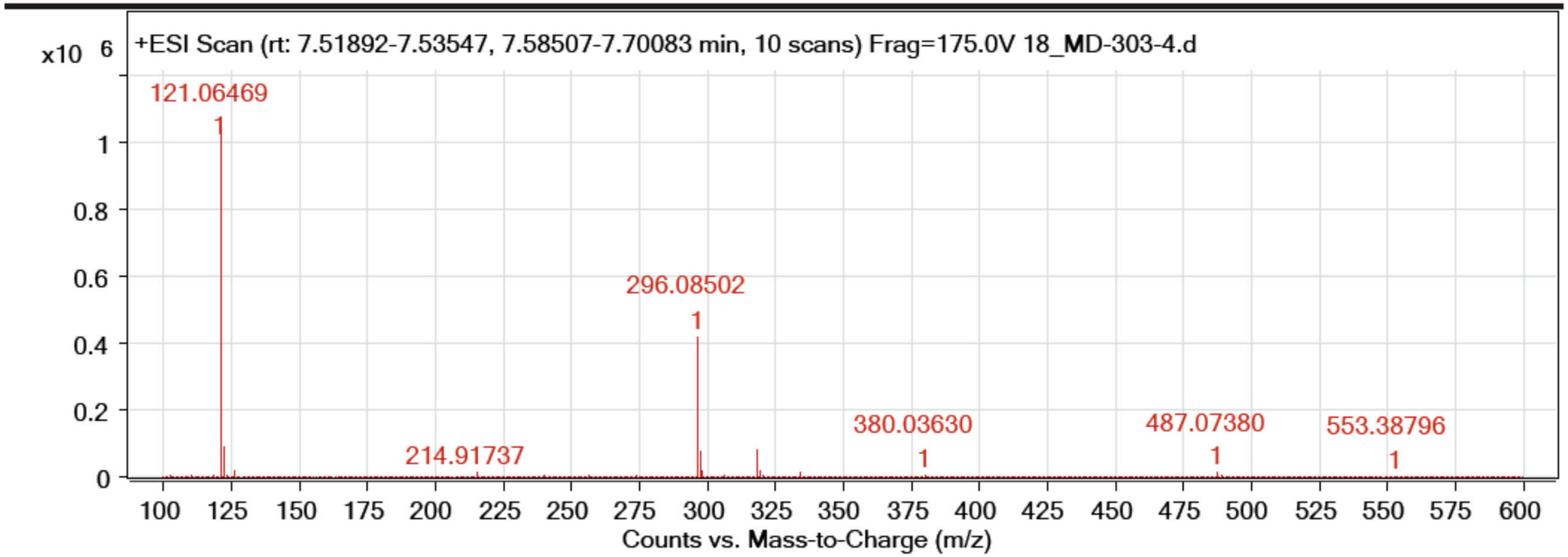

Formula Calculator Results

Formula	Best	Mass	Tgt Mass	Diff (ppm)	Ion Species	Score
C16 H13 N3 O S	True	295.07774	295.07793	0.66	C16 H14 N3 O S	98.78

Figure S63. Mass spectrum and the result of the HRMS analysis for compound **3**.

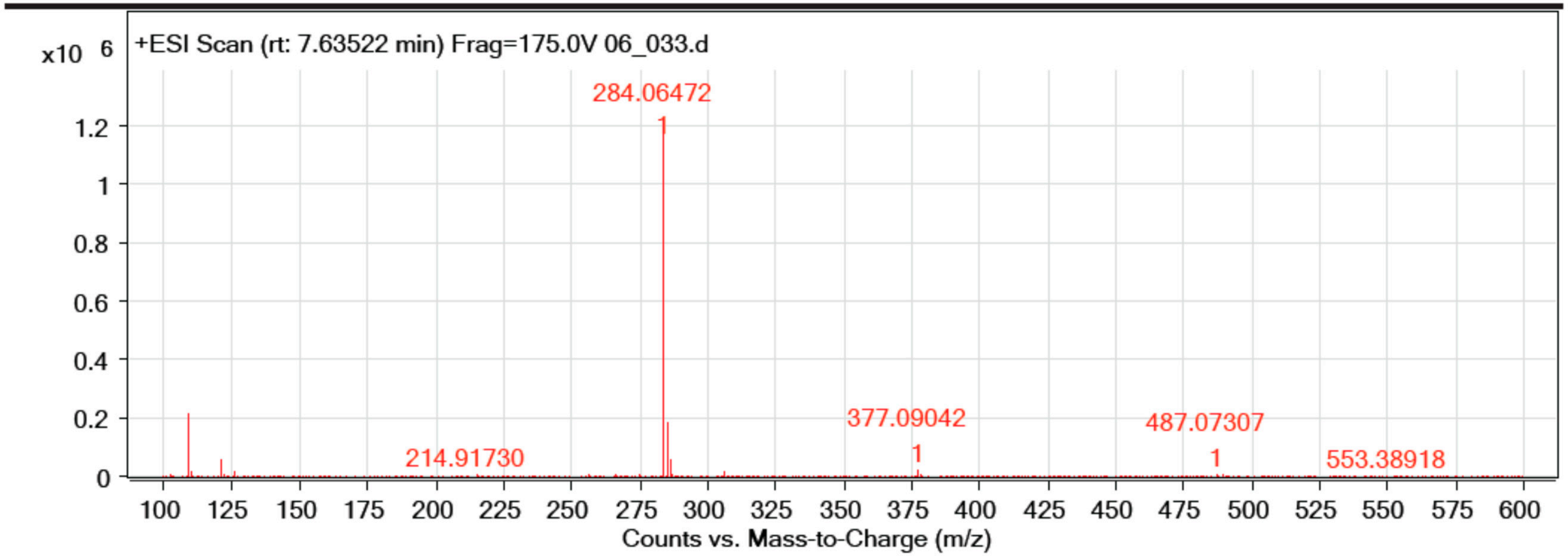

Formula Calculator Results

Formula	Best	Mass	Tgt Mass	Diff (ppm)	Ion Species	Score
C15 H10 F N3 S	True	283.05749	283.05795	1.6	C15 H11 F N3 S	97.27

Figure S64. Mass spectrum and the result of the HRMS analysis for compound **4**.

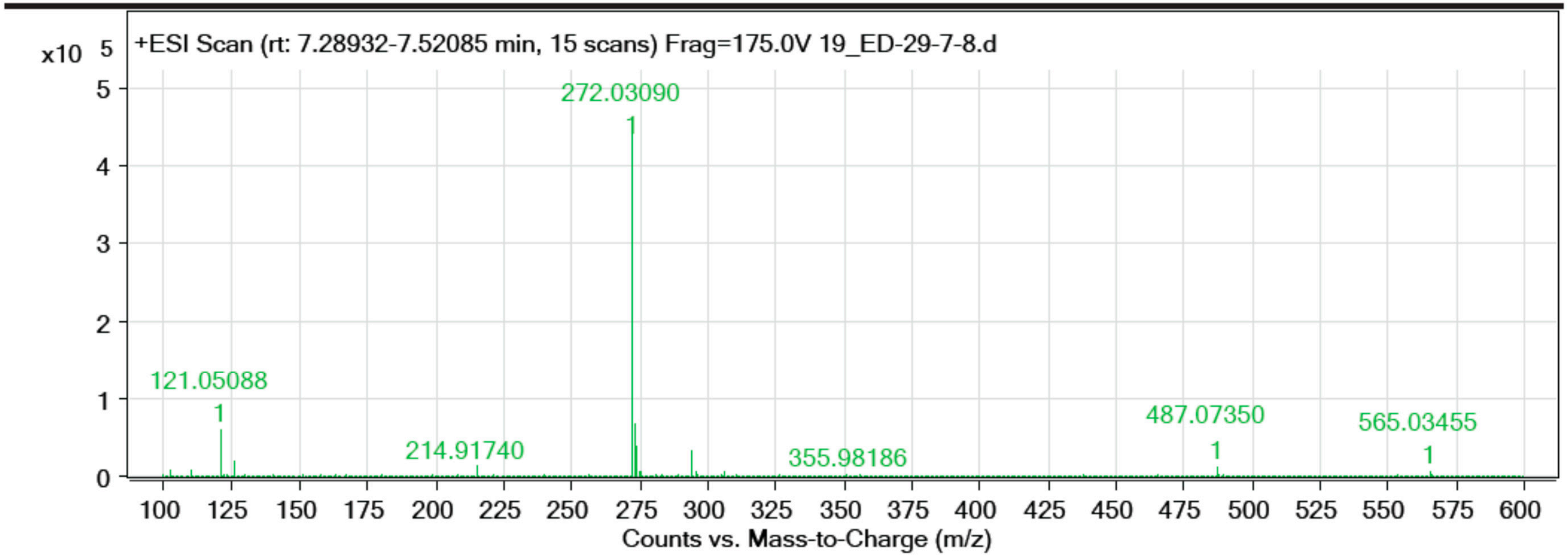

Formula Calculator Results

Formula	Best	Mass	Tgt Mass	Diff (ppm)	Ion Species	Score
C13 H9 N3 S2	True	271.02361	271.02379	0.66	C13 H10 N3 S2	97.99

Figure S65. Mass spectrum and the result of the HRMS analysis for compound **5**.

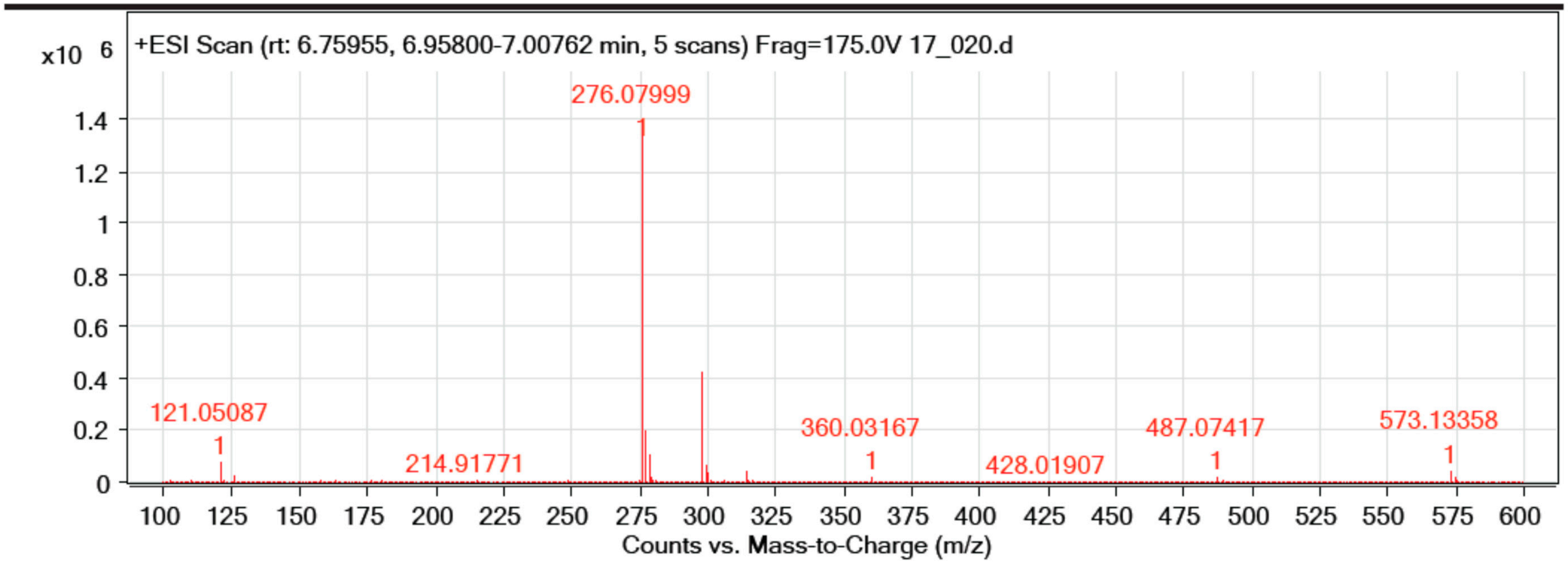

Formula Calculator Results

Formula	Best	Mass	Tgt Mass	Diff (ppm)	Ion Species	Score
C13 H13 N3 O2 S	True	275.07275	275.07285	0.35	C13 H14 N3 O2 S	92.17

Figure S66. Mass spectrum and the result of the HRMS analysis for compound **6**.

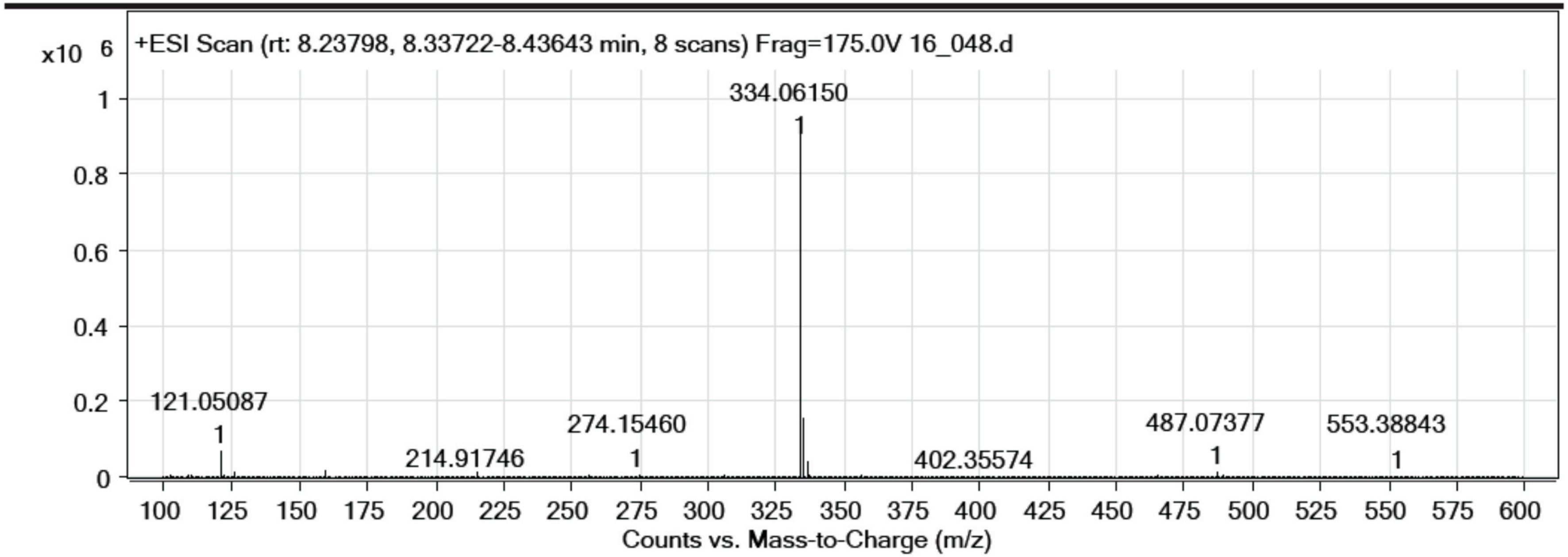

Formula Calculator Results

Formula	Best	Mass	Tgt Mass	Diff (ppm)	Ion Species	Score
C16 H10 F3 N3 S	True	333.05423	333.05475	1.57	C16 H11 F3 N3 S	96.49

Figure S67. Mass spectrum and the result of the HRMS analysis for compound **7**.

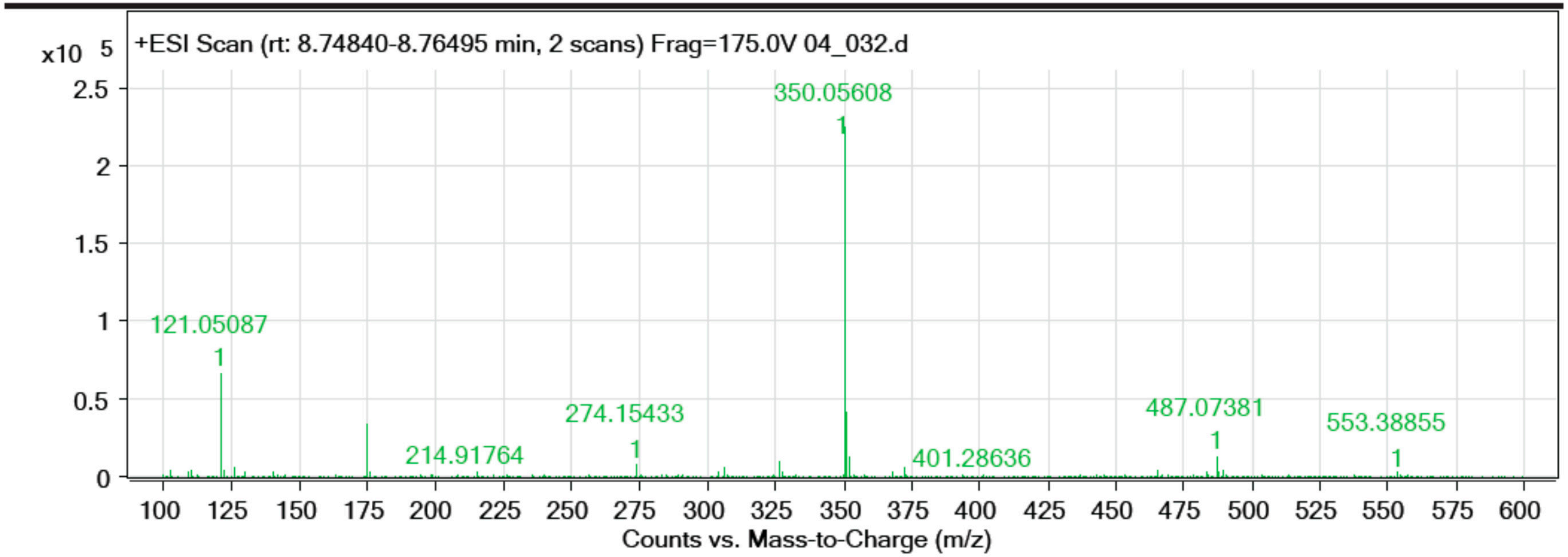

Formula Calculator Results

Formula	Best	Mass	Tgt Mass	Diff (ppm)	Ion Species	Score
C16 H10 F3 N3 O S	True	349.04885	349.04967	2.33	C16 H11 F3 N3 O S	97.62

Figure S68. Mass spectrum and the result of the HRMS analysis for compound **8**.

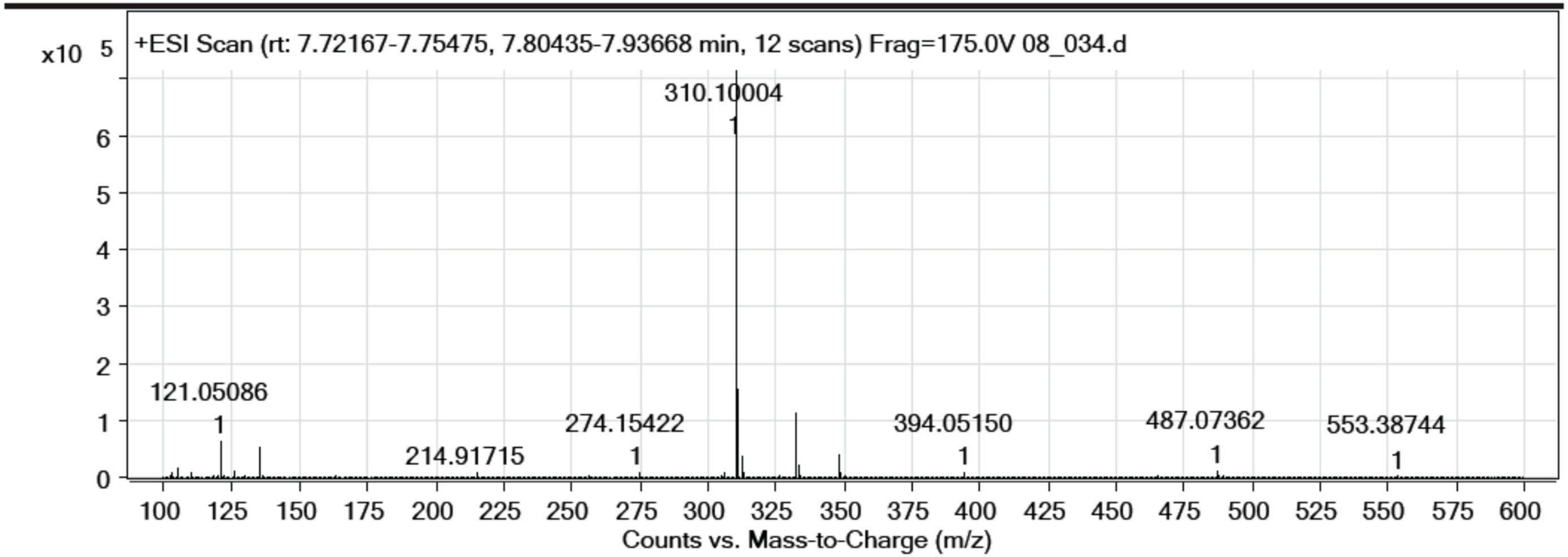

Formula Calculator Results

Formula	Best	Mass	Tgt Mass	Diff (ppm)	Ion Species	Score
C17 H15 N3 O S	True	309.0928	309.09358	2.53	C17 H16 N3 O S	95.06

Figure S69. Mass spectrum and the result of the HRMS analysis for compound **9**.

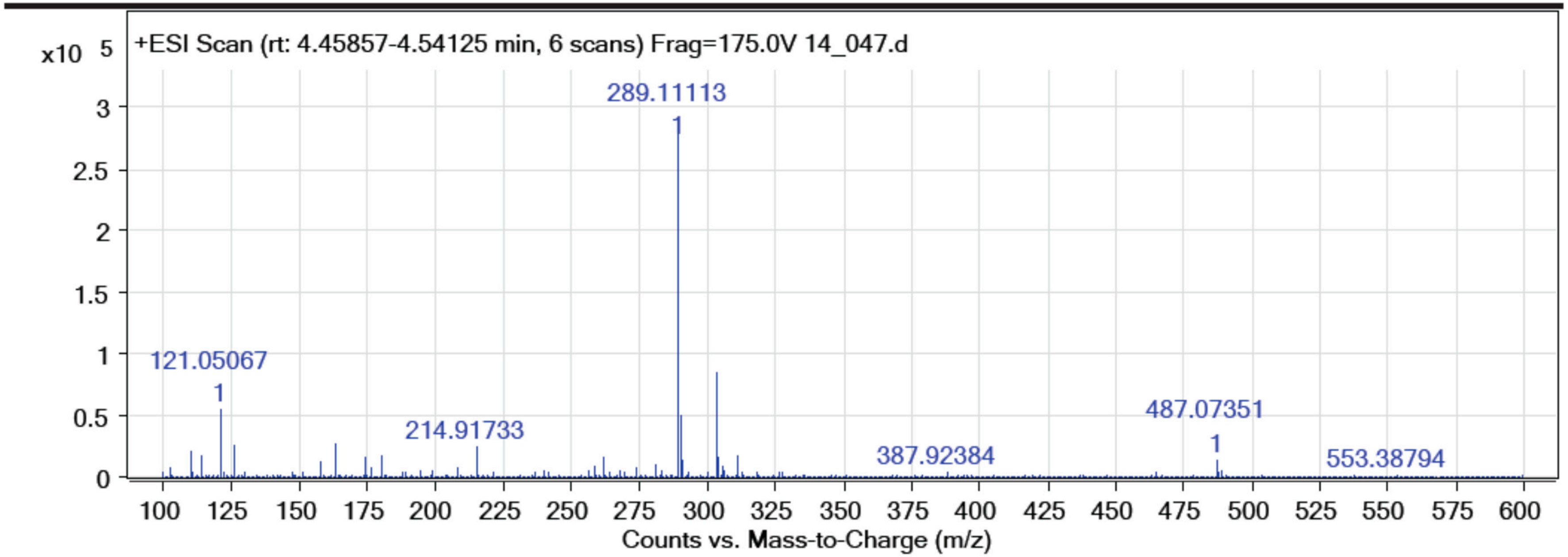

Formula Calculator Results

Formula	Best	Mass	Tgt Mass	Diff (ppm)	Ion Species	Score
C14 H16 N4 O S	True	288.10402	288.10448	1.62	C14 H17 N4 O S	96.71

Figure S70. Mass spectrum and the result of the HRMS analysis for compound **10**.

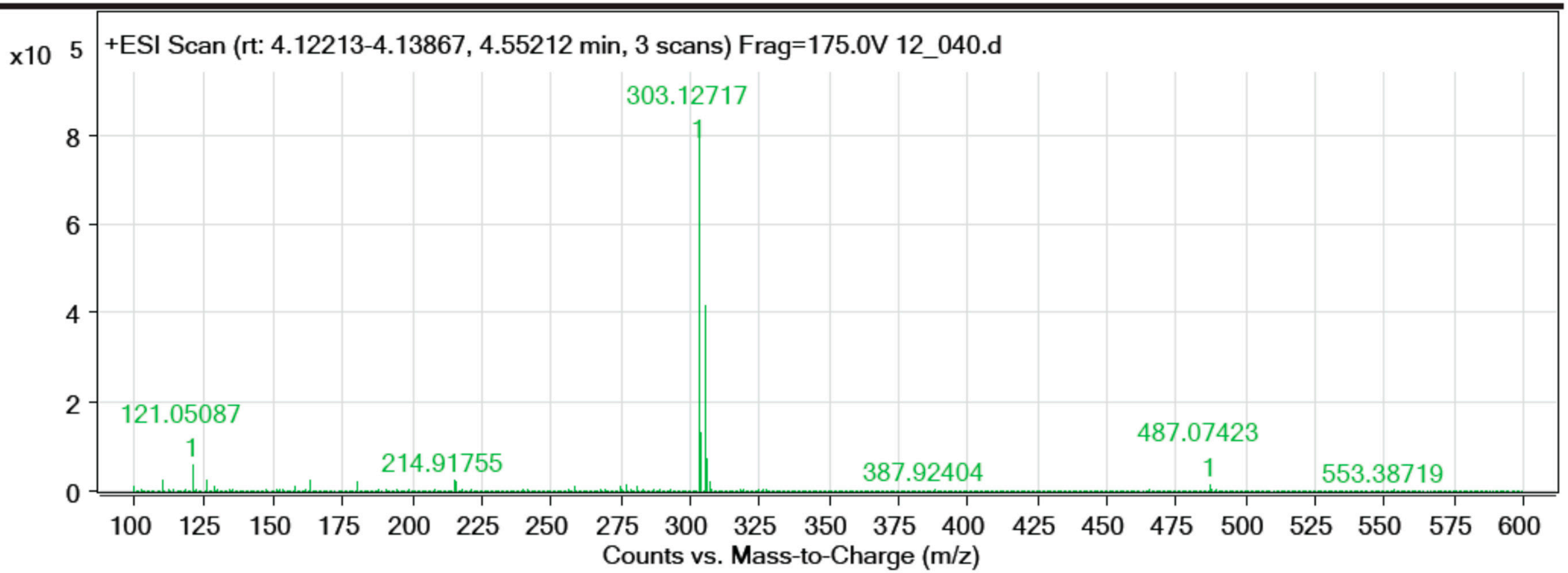

Formula Calculator Results

Formula	Best	Mass	Tgt Mass	Diff (ppm)	Ion Species	Score
C15 H18 N4 O S	True	302.11994	302.12013	0.62	C15 H19 N4 O S	95.53

Figure S71. Mass spectrum and the result of the HRMS analysis for compound **11**.

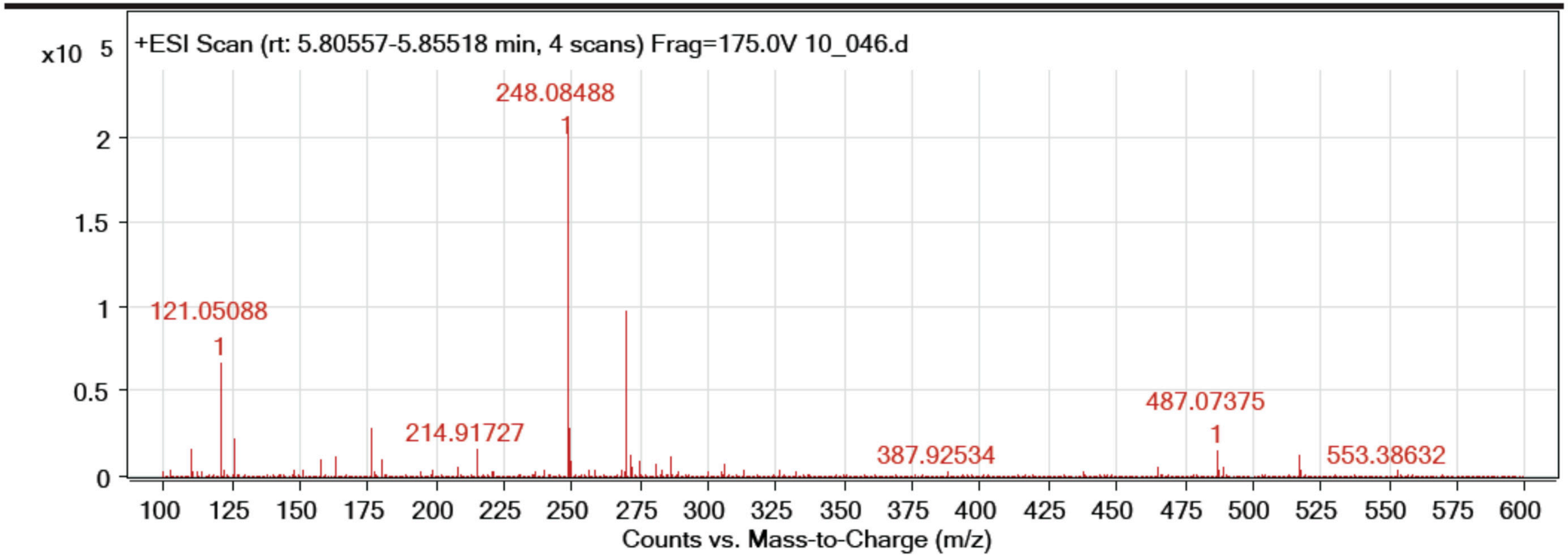

Formula Calculator Results

Formula	Best	Mass	Tgt Mass	Diff (ppm)	Ion Species	Score
C12 H13 N3 O S	True	247.07759	247.07793	1.38	C12 H14 N3 O S	98.63

Figure S72. Mass spectrum and the result of the HRMS analysis for compound **12**.

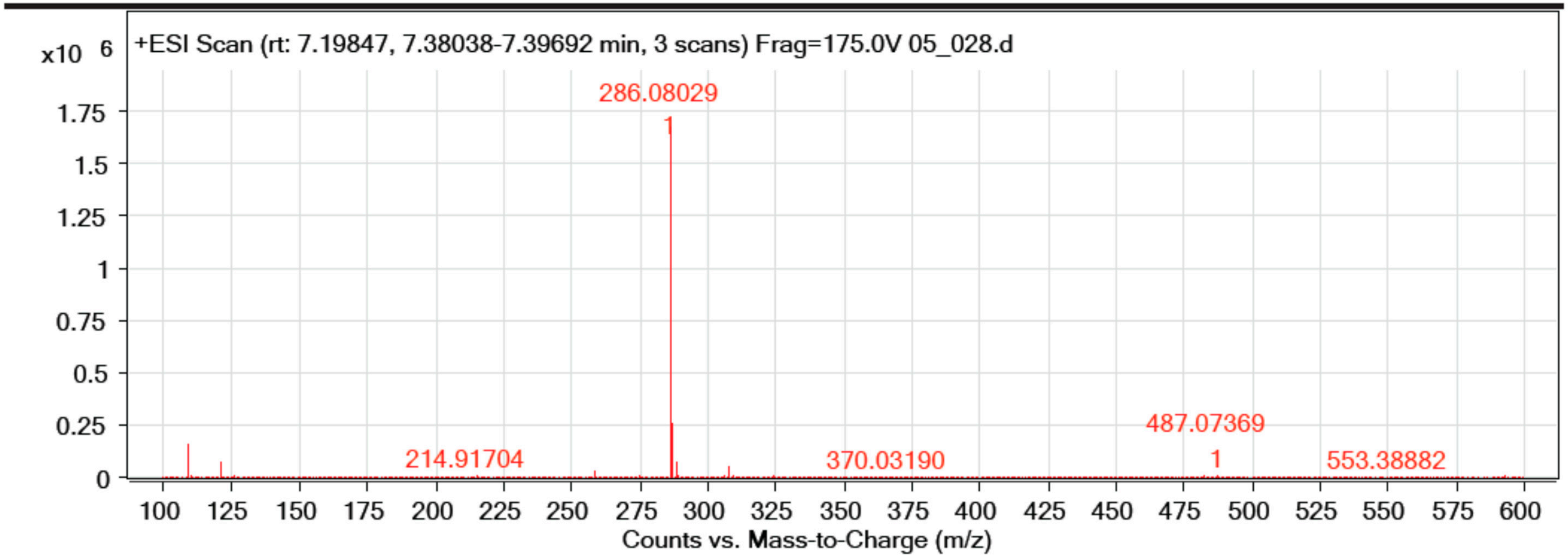

Formula Calculator Results

Formula	Best	Mass	Tgt Mass	Diff (ppm)	Ion Species	Score
C15 H12 F N3 S	True	285.07306	285.0736	1.88	C15 H13 F N3 S	96.38

Figure S73. Mass spectrum and the result of the HRMS analysis for compound **13**.

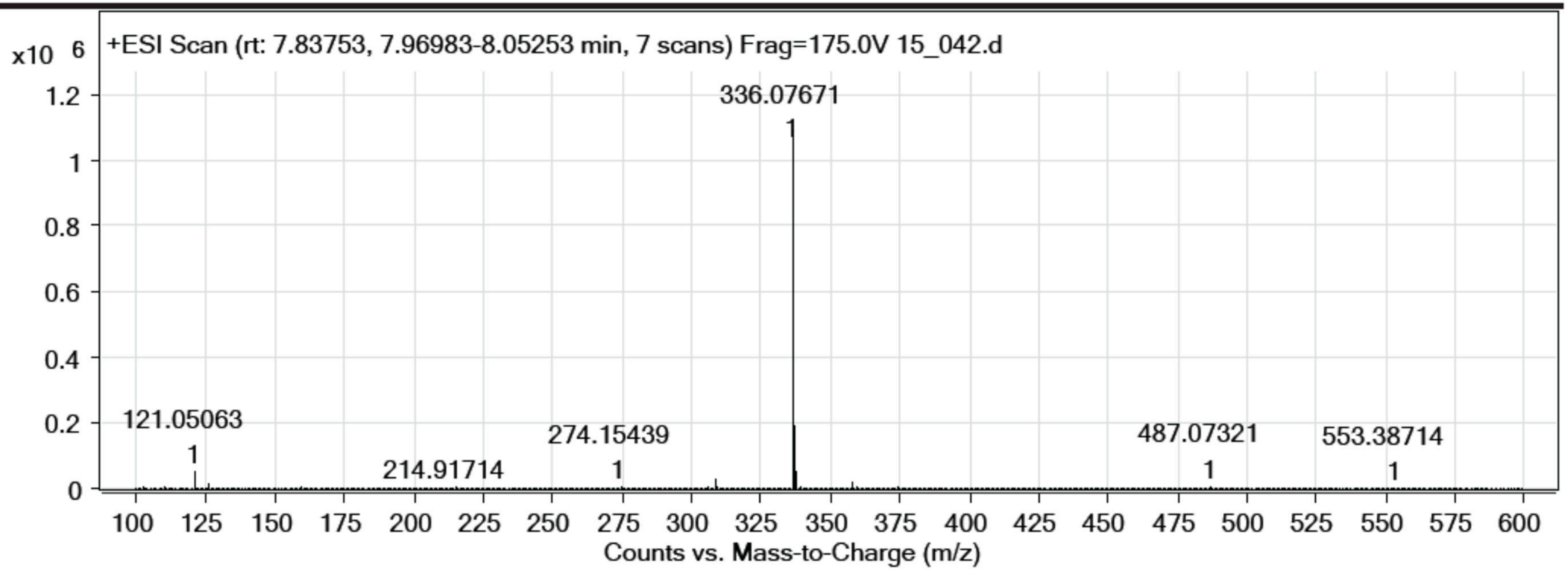

Formula Calculator Results

Formula	Best	Mass	Tgt Mass	Diff (ppm)	Ion Species	Score
C16 H12 F3 N3 S	True	335.06947	335.0704	2.78	C16 H13 F3 N3 S	95.91

Figure S74. Mass spectrum and the result of the HRMS analysis for compound **14**.

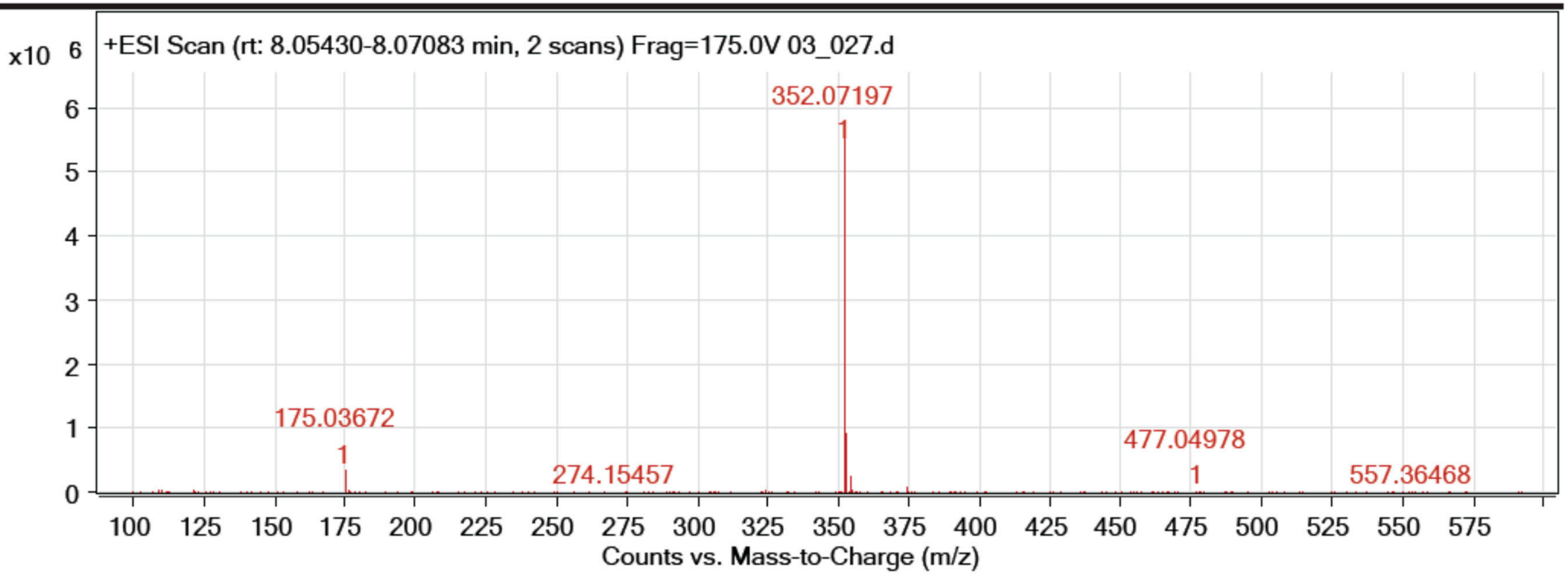

Formula Calculator Results

Formula	Best	Mass	Tgt Mass	Diff (ppm)	Ion Species	Score
C16 H11 F3 N3 O S	True	350.05555	350.05749	5.54	C16 H12 F3 N3 O S	37.49
C16 H12 F3 N3 O S	True	351.06471	351.06532	1.73	C16 H13 F3 N3 O S	95.48

Figure S75. Mass spectrum and the result of the HRMS analysis for compound **15**.

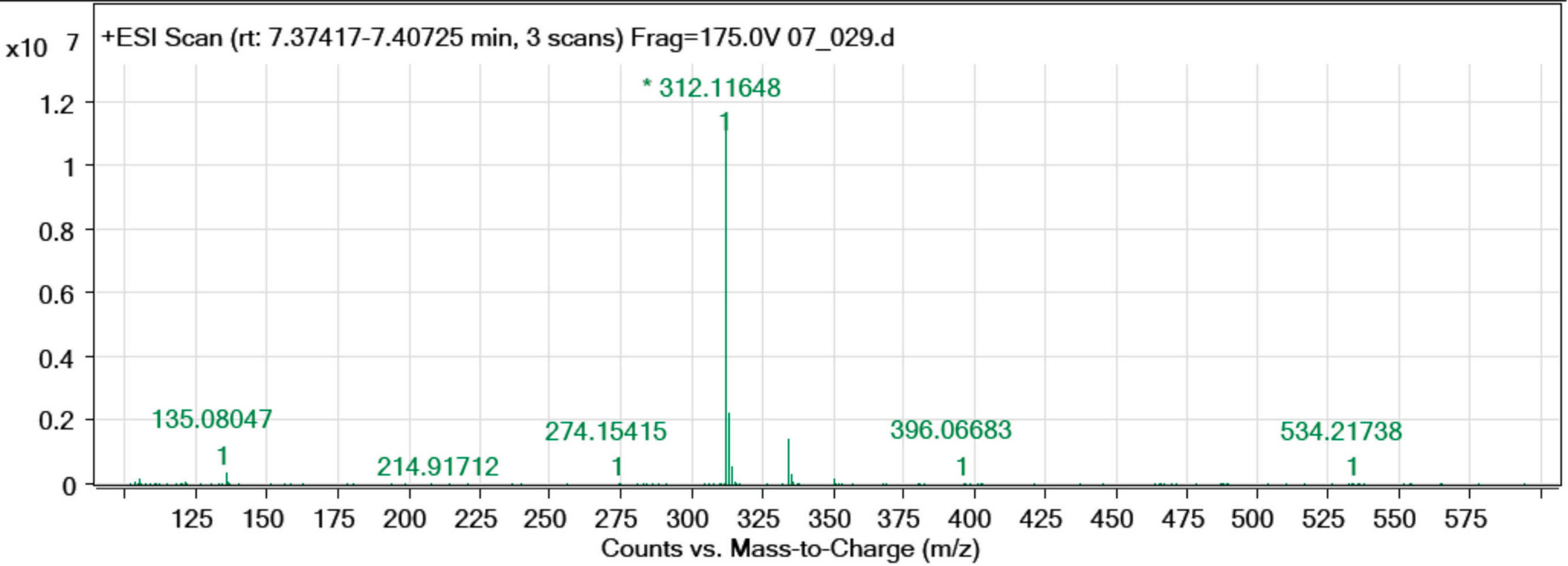

Formula Calculator Results

Formula	Best	Mass	Tgt Mass	Diff (ppm)	Ion Species	Score
C17 H17 N3 O S	True	311.10905	311.10923	0.6	C17 H18 N3 O S	97.23

Figure S76. Mass spectrum and the result of the HRMS analysis for compound **16**.

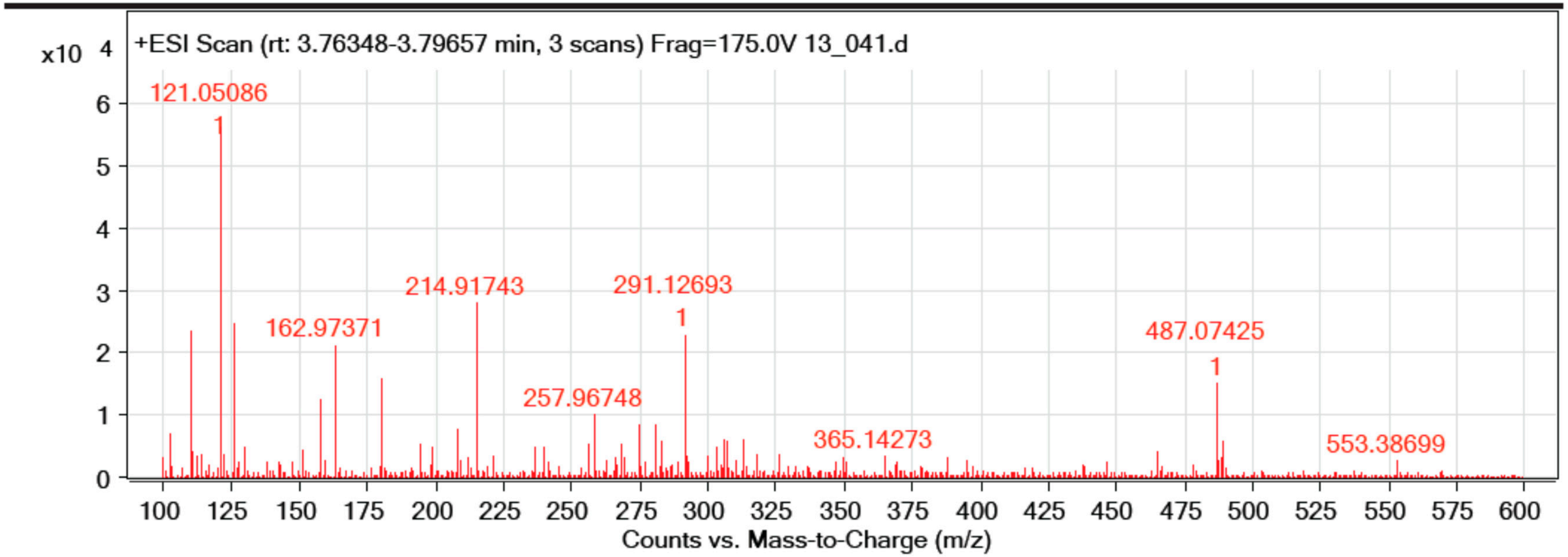

Formula Calculator Results

Formula	Best	Mass	Tgt Mass	Diff (ppm)	Ion Species	Score
C14 H18 N4 O S	True	290.11972	290.12013	1.4	C14 H19 N4 O S	95.24

Figure S77. Mass spectrum and the result of the HRMS analysis for compound **17**.

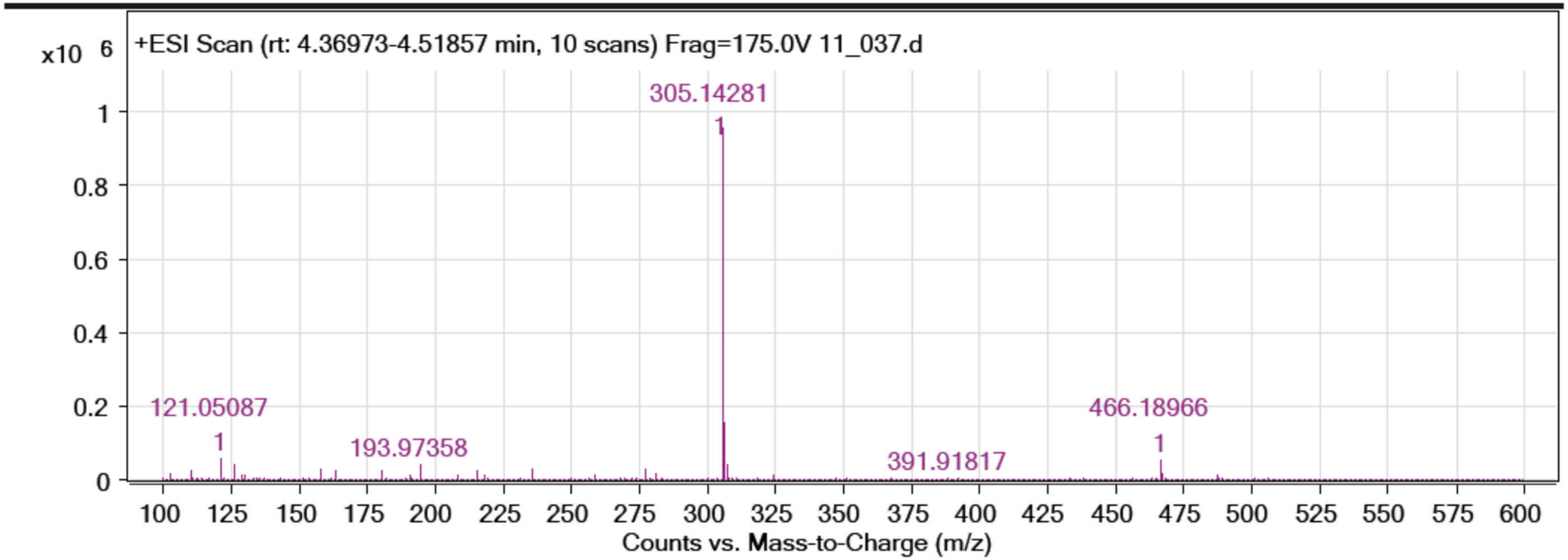

Formula Calculator Results

Formula	Best	Mass	Tgt Mass	Diff (ppm)	Ion Species	Score
C15 H20 N4 O S	True	304.13556	304.13578	0.72	C15 H21 N4 O S	97.77

Figure S78. Mass spectrum and the result of the HRMS analysis for compound **18**.

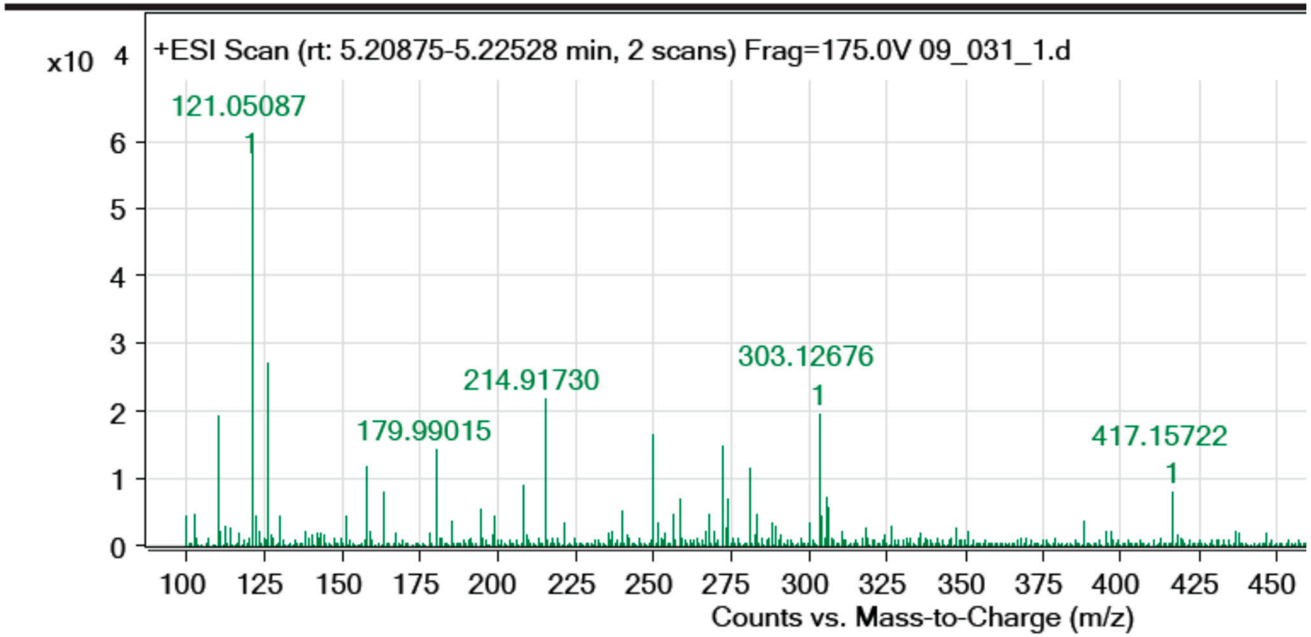

Formula Calculator Results

Formula	Best	Mass	Tgt Mass	Diff (ppm)	Ion Species	Score
C12 H15 N3 O S	True	249.09357	249.09358	0.04	C12 H16 N3 O S	95.5

Figure S79. Mass spectrum and the result of the HRMS analysis for compound **19**.

3. Cartesian coordinates of ligands docked into AChE and BChE

Molecule 1 docked into AChE

N	0.578	28.912	35.616
C	1.905	28.793	35.904
C	2.488	28.580	34.656
N	1.507	28.569	33.706
N	0.378	28.761	34.294
C	2.683	28.851	37.107
C	4.089	28.691	36.924
C	4.659	28.481	35.619
H	5.752	28.364	35.525
C	3.891	28.422	34.499
H	4.340	28.258	33.505
C	4.925	28.740	38.061
H	6.014	28.620	37.932
C	4.419	28.933	39.324
H	5.091	28.970	40.198
C	3.031	29.082	39.482
C	2.171	29.044	38.409
H	1.086	29.164	38.565
Cl	2.388	29.317	41.085
C	-0.572	29.066	36.515
H	-0.217	28.923	37.562
C	-1.609	27.986	36.211
H	-2.483	28.103	36.894
H	-1.174	26.960	36.257
H	-1.912	27.984	35.138
C	-1.157	30.469	36.399
H	-2.031	30.586	37.082
H	-1.418	30.723	35.345
H	-0.386	31.255	36.574

Molecule 4 docked into AChE

C	-2.482	28.098	35.517
H	-3.478	27.893	35.944
C	-2.134	27.555	34.279
C	-0.872	27.818	33.747
H	-0.592	27.387	32.771
C	0.038	28.617	34.431
H	1.034	28.829	34.007
C	-0.337	29.141	35.659
C	-1.583	28.895	36.216
H	-1.855	29.324	37.195
F	0.535	29.905	36.331
C	-3.121	26.710	33.499
H	-3.334	25.753	34.029
H	-4.140	27.161	33.524
N	-2.696	26.465	32.138
N	-2.517	25.195	31.706
N	-2.103	25.213	30.492
C	-1.992	26.522	30.090
C	-1.588	27.057	28.845
H	-1.298	26.397	28.010
C	-2.365	27.345	31.156
C	-1.567	28.422	28.707
H	-1.263	28.877	27.749
C	-1.938	29.247	29.802
C	-2.342	28.755	31.054
S	-1.952	30.986	29.796
C	-2.647	29.802	31.988
H	-2.977	29.625	33.026
C	-2.482	31.033	31.445
H	-2.666	31.970	31.997

Molecule 17 docked into AChE

S	3.274	28.938	39.542
C	2.961	28.674	37.870
C	1.712	29.137	37.525
C	1.008	29.724	38.624

H	-0.002	30.160	38.547
C	1.733	29.680	39.777
H	1.378	30.072	40.745
C	3.884	27.947	36.923
H	3.734	26.843	36.973
H	4.943	27.993	37.270
C	3.739	28.472	35.479
H	4.259	27.821	34.738
H	4.325	29.405	35.308
C	2.287	28.647	35.159
C	1.361	28.978	36.124
N	1.660	28.568	33.964
N	0.397	28.824	34.137
N	0.197	29.071	35.444
C	-1.128	29.392	35.954
H	-1.225	29.126	37.032
H	-1.902	28.707	35.536
C	-1.486	30.856	35.706
H	-2.589	31.017	35.682
H	-1.251	31.172	34.663
N	-0.833	31.705	36.692
C	-0.370	32.965	36.115
H	-1.194	33.524	35.613
H	0.282	32.804	35.225
C	0.318	33.799	37.186
H	0.743	34.740	36.764
H	1.266	33.324	37.531
O	-0.555	34.060	38.264
C	-1.006	32.851	38.838
H	-1.638	33.034	39.738
H	-0.174	32.292	39.326
C	-1.729	31.996	37.809
H	-2.154	31.068	38.258
H	-2.683	32.463	37.471

Molecule 1 docked into BChE

N	131.042	112.533	41.066
C	131.022	113.642	41.858
C	130.332	113.215	42.992
N	129.969	111.910	42.823
N	130.392	111.521	41.672
C	131.530	114.977	41.735
C	131.288	115.821	42.859
C	130.578	115.345	44.018
H	130.416	116.032	44.865
C	130.102	114.075	44.100
H	129.558	113.725	44.993
C	131.762	117.150	42.816
H	131.584	117.811	43.681
C	132.438	117.643	41.727
H	132.800	118.685	41.716
C	132.660	116.797	40.627
C	132.222	115.493	40.617
H	132.411	114.853	39.739
Cl	133.509	117.424	39.241
C	131.552	112.358	39.701
H	131.912	113.348	39.335
C	130.420	111.895	38.786
H	130.807	111.762	37.749
H	129.543	112.582	38.822
H	129.919	110.975	39.169
C	132.729	111.390	39.690
H	133.116	111.257	38.653
H	132.468	110.415	40.163
H	133.533	111.707	40.395

Molecule 4 docked into BChE

C	132.970	115.489	37.287
H	132.973	114.391	37.388
C	133.288	116.286	38.388
C	133.283	117.674	38.251
H	133.531	118.307	39.119
C	132.970	118.270	37.034

H	132.971	119.368	36.926
C	132.657	117.450	35.960
C	132.650	116.067	36.064
H	132.396	115.439	35.194
F	132.346	118.014	34.784
C	133.666	115.659	39.714
H	134.743	115.833	39.946
H	133.674	114.546	39.642
N	132.822	116.102	40.804
N	132.905	117.378	41.246
N	132.105	117.538	42.236
C	131.468	116.345	42.478
C	130.494	116.017	43.449
H	130.130	116.772	44.166
C	131.935	115.401	41.559
C	130.015	114.733	43.473
H	129.251	114.441	44.213
C	130.506	113.780	42.541
C	131.473	114.064	41.563
S	130.015	112.114	42.447
C	131.803	112.921	40.761
H	132.543	112.935	39.943
C	131.098	111.822	41.126
H	131.203	110.841	40.632

Molecule 9 docked into BChE

C	131.765	114.410	41.555
H	132.310	114.534	40.604
C	131.414	115.540	42.288
C	130.727	115.354	43.494
H	130.440	116.233	44.095
C	130.401	114.086	43.946
H	129.858	113.963	44.898
C	130.760	112.961	43.195
C	131.446	113.124	41.992
H	131.734	112.245	41.392
C	131.806	116.922	41.826

H	131.848	117.651	42.669
H	131.000	117.411	41.231
C	133.125	116.851	41.059
H	133.430	115.793	40.882
H	133.978	117.179	41.697
N	133.086	117.601	39.811
C	133.043	117.118	38.543
C	133.025	115.819	37.979
C	133.015	118.266	37.748
C	133.043	114.507	38.563
H	133.078	114.316	39.649
C	132.978	115.787	36.577
C	133.012	113.530	37.623
H	133.020	112.454	37.864
S	132.959	114.145	36.004
C	132.951	116.947	35.758
H	132.916	116.848	34.660
C	132.969	118.191	36.337
H	132.948	119.105	35.720
N	133.042	119.362	38.574
N	133.083	118.951	39.792
O	130.394	111.762	43.717
C	130.707	111.520	45.071
H	130.406	110.536	45.500
H	130.287	112.340	45.699
H	131.800	111.674	45.230

4. Estimated free energies of binding obtained by molecular docking

Table S1. Free energies of binding, ΔG_{bind} obtained by molecular docking of listed molecules into the active site of BChE, along with the number of conformational clusters and distribution of conformations.

Compound	ΔG_{bind}/kcal mol^{-1}		Number of distinctive conformational clusters	Distribution of conformations within clusters with $n > 1$ (n = cluster population)
	lowest	highest		
**1**	−5.92	−5.71	1	25
**3**	−6.13	−5.85	4	7, 11, 6
**4**	−5.96	−5.61	5	12, 8, 2, 2
**5**	−5.91	−5.52	5	2, 15, 5, 2
**6**	−5.85	−4.82	10	5, 4, 6, 2, 3
**9**	−6.37	−5.45	10	4, 3, 5, 4, 3, 2, 3
**13**	−6.11	−5.70	6	5, 8, 9
Galantamine	−6.26	−6.17	1	25

5. Crystallographic details and crystal structure of compound 5

Table S2. Crystallographic, data collection and refinement data.

Compound	**5**
Empirical formula	$C_{26}H_{18}N_6S_4$
Formula wt. / g mol^{-1}	542.70

Colour	yellow
Crystal dimensions / mm	0.22 x 0.09 x 0.02
Space group	$P\,2_1/n$
a / Å	9.98930(10)
b / Å	13.47420(10)
c / Å	18.3819(3)
α / °	90
β / °	97.2340(10)
γ / °	90
Z	2
V / Å^3	2454.47(5)
D_{calc} / g cm^{-3}	1.469
λ / Å	1.54179 (Cu$K\alpha$)
μ / mm^{-1}	3.792
Θ range / °	4.08 – 79.73
T / K	293(2)
Diffractometer type	Synergy S
Range of h, k, l	$-11 < h < 12$; $-17 < k < 17$; $-23 < l < 23$
Reflections collected	38715
Independent reflections	5301
Observed reflections ($I \geq 2\sigma$)	4621
Absorption correction	Multi-scan

T_{min}, T_{max}	0.5661; 1.0000
R_{int}	0.0658
R (F)	0.0519
R_w (F^2)	0.1647
Goodness of fit	0.810
H atom treatment	Constrained
No. of parameters	325
No. of restraints	0
$\Delta\rho_{max}$, $\Delta\rho_{min}$ (eÅ^{-3})	0.454; -0.495

6. Reorganized experimental results on the inhibitory activity of 1-19 (Table S2)

Table S3. Calculated IC_{50} values for compounds **1-19**.

Compound	Structure	IC_{50} (μM) AChE	IC_{50} (μM) BChE	Compound	Structure	IC_{50} (μM) AChE	IC_{50} (μM) BChE
**1**	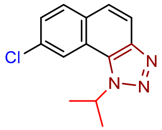	59.9	39.6	**2**	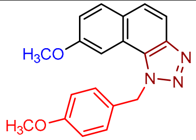	-	-
**3**	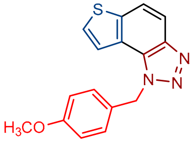	116.0	25.5	**5**	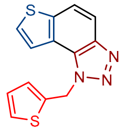	118.6	23.4
**4**	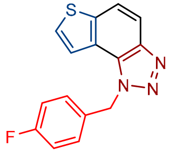	97.0	37.5	**13**	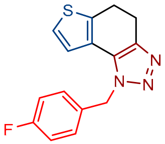	-	58.0
**6**	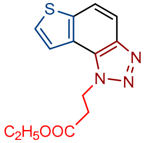	-	39.6	**Galantamine**		0.15	7.9
**7**	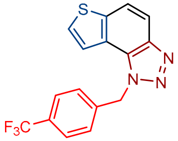	139.6	> 150	**14**	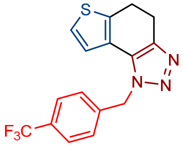	117.6	> 150

**8**	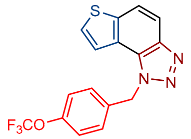	68.0	213.5
**9**	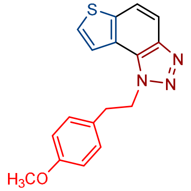	-	18.6
**10**	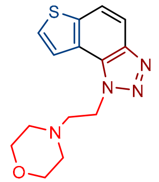	-	-
**11**	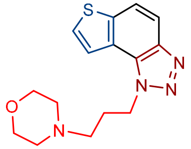	-	-
**12**	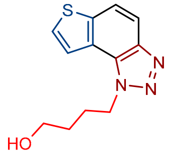	-	106.7
**15**	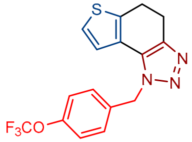	88.7	-
**16**	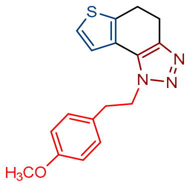	145.3	119.4
**17**	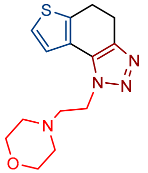	45.6	-
**18**	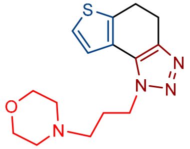	-	-
**19**	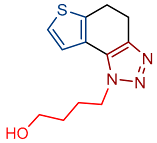	-	74.1
